# Structure–Property Relationships of Lignin-Derived Semiaromatic Poly(ether ester)s

**DOI:** 10.1021/acs.macromol.5c00380

**Published:** 2025-07-11

**Authors:** Ryan K. Maynard, Kush G. Patel, Huiming Wu, Frida C. Knudsen, Imrie C. Ross, DeMichael D. Winfield, Jason J. Locklin

**Affiliations:** † Department of Chemistry, Franklin College of Arts and Sciences, 1355 University of Georgia, 140 Cedar Street, Athens, Georgia 30602, United States; ‡ School of Chemical, Materials, and Biomedical Engineering, College of Engineering, 1355 University of Georgia, 597 D.W. Brooks Drive, Athens, Georgia 30602, United States; § New Materials Institute, 1355 University of Georgia, 220 Riverbend Road, Athens, Georgia 30602, United States

## Abstract

Semiaromatic polyesters derived from petroleum are an important class of polymers that encompass a wide variety of thermal and mechanical properties. Unfortunately, replacing the aromatic component with cost-competitive bioderived monomers is an ongoing challenge. This work describes the synthesis of nine different polyesters made from AB monomers that can be derived from lignin, starting with phloretic, coumaric, and ferulic acids and similar derivatives. The polyesters were synthesized at >50 g scale, and full characterization of the thermal, mechanical, and rheological properties is included. The polymers exhibit excellent thermal stability, a *T*_g_ range of 16–65 °C, tensile moduli ranging from 4.6 to 1200 MPa, and elongation at break ranging from 7.5% to greater than 3800%. To examine the effects of structural permutations among the polymer series, TTS master curves were constructed for seven polyesters using melt rheology. Properties such as packing length and characteristic ratio are described and compared among the series. Finally, it is demonstrated that these polyesters are easily chemically recycled to monomer in high yield.

## Graphical abstract

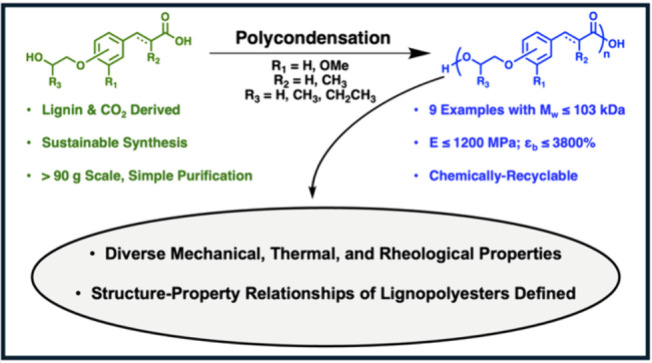

## Introduction

The global demand for plastics continues to increase, making the search for alternative feedstocks more critical than ever.^1–3^ Traditionally derived from petroleum, plastics have seen an increasing shift toward production using renewable resources like fermentation products, vegetable oils, sugars, terpenes, lignocellulosics, and various waste products.^4–6^ Although a number of biobased aliphatic polyesters such as poly­(hydroxyalkanoates),^7^ poly­(lactic acid)^8^ and poly­(butylene dicarboxylate)­s^9^ are commercially available, there are few commercially available polyesters containing biobased aromatic moieties. Two polymers of industrial interest are poly­(butylene adipate-*co*-butylene terephthalate) (PBAT)^10^ and poly­(ethylene furanoate) (PEF).^11^ In the case of PBAT, the adipic acid and butylene glycol portions can be derived from fermentation, albeit at a higher cost than the oil-derived monomer at current scales.^12–14^ However, the production of biobased terephthalic acid poses significant challenges, and production is mainly limited to the laboratory or pilot scale.^15–17^ Currently, biobased terephthalic acid is not used to produce PBAT. Biobased 2,5-furandicarboxylic acid (FDCA) can be produced through fermentation and other processes, enabling the production of fully biobased PEF, which is touted as a drop-in replacement for poly­(ethylene terephthalate).^18^ However, widespread adoption of PEF has not yet occurred.

Another feedstock of particular interest is lignin—the most abundant source of natural aromatics.^19^ Currently, lignin is a byproduct of the paper industry that is burned for energy recovery, with only about 0.2% being used for other purposes.^20–22^ Lignin is a macromolecule found in the secondary cell wall of woody biomass, with a high concentration of aromatics with varying substitutions of pendant methoxy groups.^23^ The use of lignin in polymeric materials is appealing due to its high strength, thermal stability, and its ability to provide a source of aromatic feedstock with diverse chemical architectures.^20,24–32^ Extensive research has already been conducted to incorporate lignin directly in polymer blends, functionalize lignin for compatibility with polymer matrices, and directly polymerize oligomers of lignin.^33–35^

However, the biological origin of lignin directly influences its molecular structure, which can lead to performance inconsistencies in composites and blends that use it as a direct component.^36^ For this reason, significant efforts have been undertaken to efficiently crack lignin into various “monolignols” and other small molecule byproducts via a number of depolymerization strategies.^37–39^ These monolignols, once isolated, can be chemically modified through traditional methods and then used as precursors for small molecule and polymer synthesis.^40–45^ Previous work has demonstrated that polymers possessing a broad range of thermal and mechanical properties can be synthesized from lignin-derived monomers.^37–39^ Slight changes in the monomer structure have a dramatic influence on the overall properties of the resulting polymer.

This work focuses on modifications and variations of common monolignols: coumaric, phloretic, and ferulic acids. Alkylation of the phenolic moiety was accomplished via the ring-opening of cyclic carbonates, which can be synthesized from biobased sources with carbon dioxide or through transcarbonation reactions.^38,46^ These reactions are carried out under neat conditions, offering an advantage over traditional etherification reactions that require haloalcohols and organic solvents. This synthetic strategy closely adheres to the principles of green chemistry by maximizing the atom economy of reactions and minimizing waste,^47^ while simultaneously accessing fully biobased monomers at 50–100 g scale.

In previous work, we demonstrated that amorphous phloretate- and ferulate-based polymers have a broad range of properties.^38^ From this, structural analogues were envisioned that could provide direct comparisons in terms of their thermal, mechanical, and rheological properties. In this study, the synthesis and comprehensive characterization of nine polymers are presented, each exhibiting various structural permutations designed to elucidate the effects of tacticity, substituents, structural isomerism, and regioisomerism on their properties. Furthermore, it is demonstrated that these polymers can be chemically recycled via base hydrolysis in rapid timeframes.

## Materials and Methods

### Materials

Phloretic acid, ferulic acid, (*E*)-*p*-coumaric acid, propylene carbonate, (*R*)-(+)-propylene carbonate, potassium carbonate, chloroform-*d*, and DMSO-*d6* were purchased from Oakwood Chemical. Propionic anhydride was purchased from ThermoFisher Scientific. Sodium propionate and ethylene carbonate were purchased from Fisher Scientific. α-Butylene carbonate was purchased from Ambeed, Inc. 10% palladium on carbon was purchased from Sigma-Aldrich. All other reagents and solvents were purchased from commercial suppliers and used as received without further purification.

### Synthesis of Monomers and Polymers

Starting hydroxycarboxylic acids were either purchased from commercial suppliers or synthesized. Subsequent protection via Fischer esterification and alkylation yielded nine monomers of suitable purity that were polymerized in good to excellent yields by polycondensation. Detailed synthetic procedures and characterization for each compound are included in the Supporting Information.

Polyester synthesis was conducted in a 3-neck flask, with the volume being either 100 or 200 mL depending on the amount of monomer used. Sb_2_O_3_ was added to the flask at 1 mol % relative to monomer, and 1000 ppm of 4-methoxyphenol was added to reduce undesired oxidation and darkening of the polymer. After the addition of the monomer, catalyst, and antioxidant, the flask was fitted with an internal thermocouple, a mechanical stirrer with an SP Bel-Art Safe-Lab bearing, and a short-path distillation head. The entire contents were then evacuated to <100 mTorr and backfilled three times with dry nitrogen. Under a nitrogen atmosphere, the flask was heated from 160 to 220 °C at a rate of 10 °C/h with a heating mantle while stirring at 250 rpm. Once the final temperature was reached, vacuum was slowly applied to the flask over 15 min until the pressure reached <200 mTorr. The stirring was reduced to 150 rpm, and the vacuum was held constant until the flask contents were too viscous to have efficient mixing. After this, the polymers were removed from the flask under nitrogen onto silicone release paper and collected in quantitative yields.

### Molecular Weight and Structural Characterization

^1^H NMR spectra were recorded at room temperature with a Bruker Avance Neo 600 MHz instrument (USA) at a concentration of 10 mg/mL. Peak shifts in parts per million (ppm) were reported relative to the signal of chloroform-*d* at 7.26 ppm or DMSO-*d*6 at 2.50 ppm. ^13^C NMR spectra were recorded at room temperature with a Bruker Avance II 400 MHz instrument (USA) at a concentration of 10 mg/mL. Peak shifts in parts per million (ppm) were reported relative to the signal of chloroform-*d* at 77.16 ppm or DMSO-*d*6 at 39.52 ppm.

Molecular weight and dispersity (*Đ*) analyses were conducted on a Malvern OMNISEC RESOLVE gel permeation chromatography system (USA) with a refractometer detector. Samples were dissolved in HPLC-grade chloroform at a concentration of 1 mg/mL and filtered through a 0.2 μm PTFE filter before injection. Viscotek T-Series Columns (T3000, T4000, and T6000) columns were regulated at 35 °C and were eluted with HPLC-grade chloroform at a rate of 1 mL/min. The resulting molecular weight data are reported relative to polystyrene standards.

### Thermal Characterization

Differential scanning calorimetry (DSC) analysis was conducted on a Discovery DSC 250 (TA Instruments, USA) differential scanning calorimeter equipped with an RCS 90 cooling system (TA Instruments, USA) under a 50 mL/min nitrogen purge. The samples (4 and 7 mg) were enclosed in aluminum T-zero pans. Samples were first heated from −20 to 200 °C at 10 °C/min to erase thermal history. Samples were subsequently cooled to −20 °C at 10 °C/min, followed by a second heating step to 200 °C at 10 °C/min. DSC data was reported from the second heating curve unless otherwise noted.

Thermal stability was quantified using thermogravimetric analysis (TGA) on a Discovery TGA (TA Instruments, USA). Approximately 7–12 mg of sample was heated from room temperature to 600 °C at 10 °C/min under a nitrogen atmosphere.

### Injection Molding and Mechanical Characterization

Melt extrusion was conducted using a Thermo-Fischer Haake Minilab II (USA) twin screw extruder, and samples were injection molded using a Thermo-Fischer Haake Minijet Pro (USA) injection molding system. The polyesters were extruded at *T*_g_ + 100 °C and injection molded following optimized conditions (Table S1) into ASTM D638 Type V tensile specimens with magnesium stearate as a mold release agent.

Tensile performance of polyesters was evaluated using a Shimadzu AGS-X Universal Testing Machine (USA). Samples were tested at 25.3 ± 0.3 °C with a rate of 20 mm/min, and data reported are the statistical average of 3 specimens. Poly­(α-butylene phloretate) specimens were tested at 100 mm/min due to the high % elongation at break. All other polymers were tested at 20 mm/min using ASTM D638.^48^ Note that not all samples broke within the required 5 min period.

### Gel Content Characterization

The gel content of the polyesters was determined by using Soxhlet extraction. Thimbles were stored in a room with 50% humidity before being weighed. Polymer was added to the thimble, and the final mass was recorded before subjecting to continuous extraction over 24 h in chloroform. The thimble was then dried for 24 h at 60 °C under vacuum. The thimble was allowed to equilibrate for 24 h in the same room with a relative humidity of 50% before the final mass was analyzed. The insoluble catalyst was assumed to be uniformly distributed throughout the polymer, and the theoretical catalyst mass was subtracted from the final calculation.

### Rheological Characterization

Rheological experiments were conducted on a TA Instruments Discovery Hybrid Rheometer (USA) using a 25 mm parallel plate geometry with a 2100 μm trim gap and a 2000 μm running gap. A series of frequency sweeps at 1% oscillatory strain were conducted at temperature ranges from 120–25 °C with oscillation frequencies from 600–0.1 rads/s to construct corresponding time–temperature superposition (TTS) master curves. These master curves were used to investigate the entanglement molecular weight (*M*_e_), packing length (*p*), and characteristic ratio (*C*_∞_) of each polyester. PEMC and PiPC were excluded from analysis due to the absence of a crossover point.

Melt densities of the polymers were measured using a Tinius Olsen MP1200, with a load weight of 2.16 kg, a travel distance of 6.35 cm, and a total of 3 captures for each polymer. The analysis temperature was optimized according to the polymer viscosity of the melt. The melt densities of PiPP, (*R*)-PiPP, and PiPHF were measured at 90 °C; *o*-PiPP and PEMP were measured at 80 °C; PiPMP and PαBP were measured at 60 °C.

### Chemical Recycling to Monomer Study

The polymer recyclability was demonstrated by using (*R*)-PiPP. 75.0 g of (*R*)-PiPP (*M*_w_ = 29.0 kDa, *Đ* = 1.4) was added to 750 mL of 2 M KOH in a solution of 2:1 water:ethanol in a 2 L steel pressure vessel (Series 4350; Parr Instrument Company, USA). The mixture was heated to 150 °C for 60 min with stirring before being cooled to room temperature. The resulting solution was filtered, cooled to 5 °C, and then acidified with 6 M HCl to pH = 2 resulting in a white precipitate of (*R*)-isopropyl phloretate ((*R*)-iPP). The precipitate was filtered and dried in a vacuum oven at 60 °C for 24 h to yield 70.1 g of tan powder (80%). After this, 55 g of the recycled (*R*)-iPP was added to a 100 mL 3-neck flask and the previously described synthesis was followed to yield the polymer (*M*_w_ = 42.2 kDa, Đ: 1.5).

## Results and Discussion

### Monomer Synthesis

Each of the starting hydroxycarboxylic acids in this study were derived wholly from lignin derived compounds or synthesized from precursors that can be extracted from lignin. In the case of phloretic acid and (*E*)-*p*-coumaric acid, the starting materials were available from commercial suppliers. The remaining four hydroxycarboxylic acids were accessed through high-yielding, scalable transformations, such as the Perkins condensation, Fischer esterification, or palladium-catalyzed hydrogenations, which were conducted on a greater than 100 g scale. Technoeconomic assessments of lignin-derived compounds, including *p*-coumaric and ferulic acid, indicate that recovery is challenging but feasible with appropriate process optimization and feedstock selection.^49,50^ A summary of the commercially available and synthesized starting carboxylic acids is shown in Figure 1.

**Figure 1:**
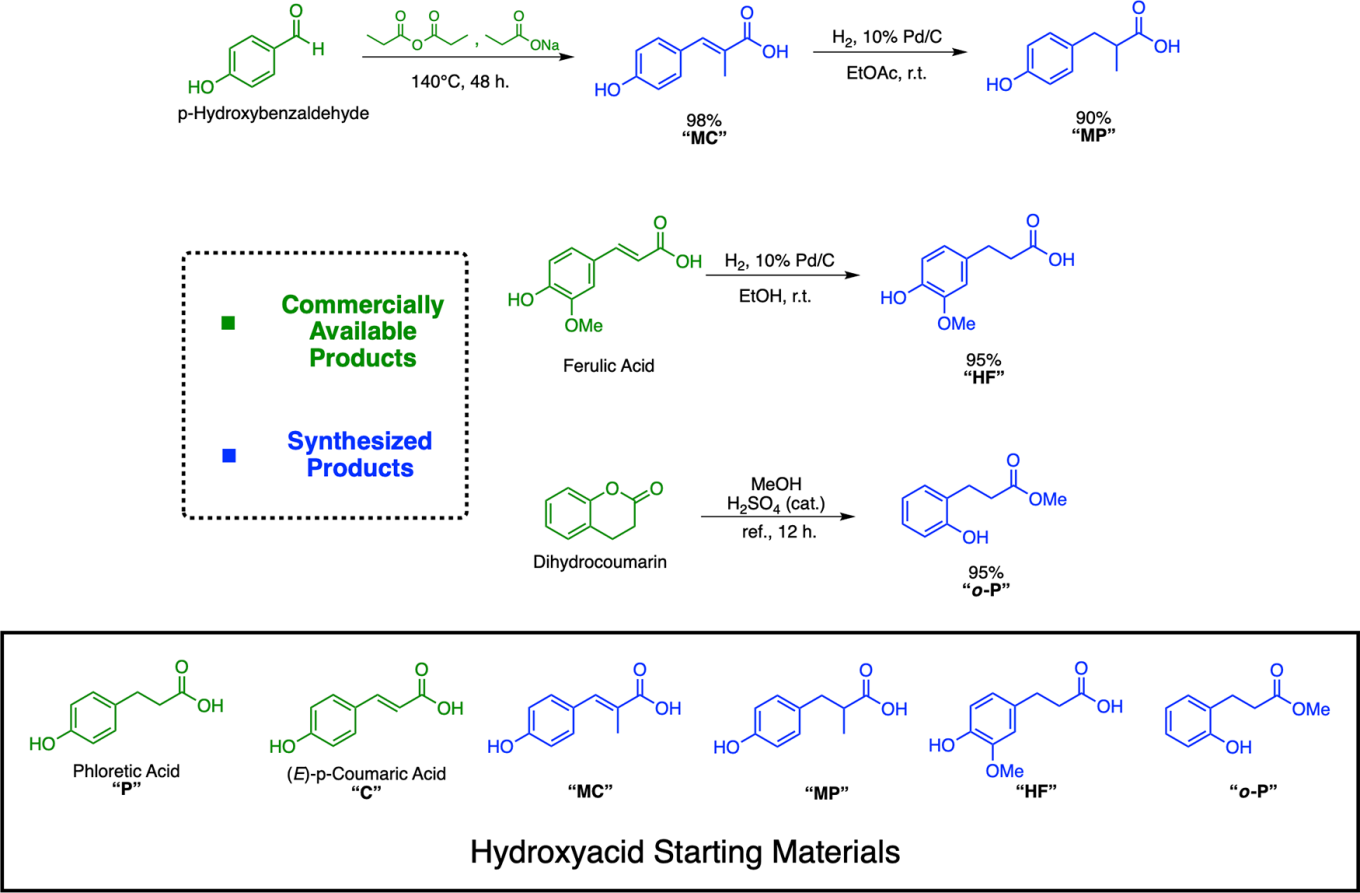
Commercially available (green) and synthesized (blue) hydroxycarboxylic acids used in this study.

Hydroxycarboxylic acids were protected via Fischer esterification in excellent to quantitative yields before being alkylated through the ring opening of cyclic carbonates (Scheme 1). Selected cyclic carbonates include biobased ethylene, propylene, and (*R*)-(+)-propylene carbonates, alongside α-butylene carbonate, which is currently petroleum-derived. This synthetic strategy closely adheres to the principles of green chemistry, being highly atom-efficient while simultaneously avoiding the use of hazardous solvents and alkyl halides that are commonly used in phenolic alkylations.

**Scheme 1:**
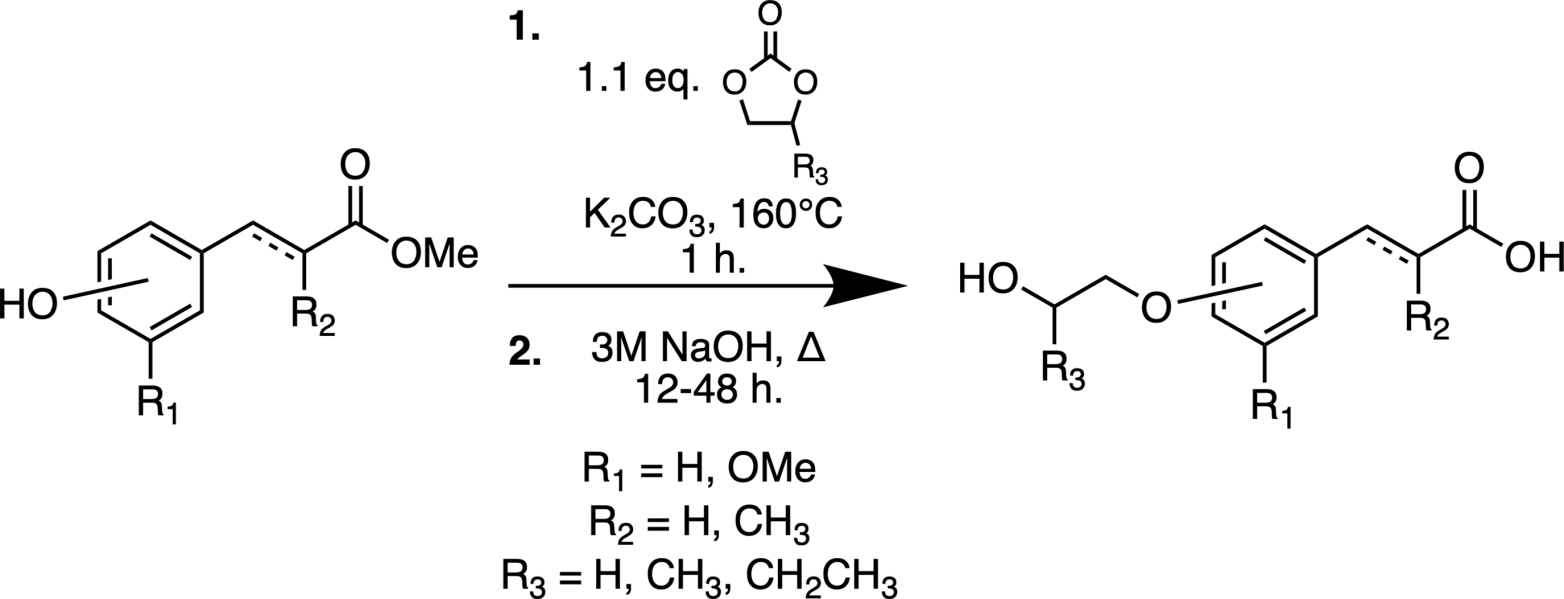
Synthesis of Lignin-Derived Monomers Using Cyclic Carbonates

### Polymer Synthesis

Polymerization was conducted using melt-polycondensation with antimony trioxide (Sb_2_O_3_) as a catalyst. Sb_2_O_3_ is resistant to poisoning from water evolved over the course of the reaction and has sufficient catalytic activity to produce polyesters with molecular weights high enough to perform mechanical and rheological analysis.^51^ High temperatures and prolonged reaction times can cause darkening of all polymers; therefore, 1000 ppm of MEHQ was added to each reaction as an antioxidant.

Significant variations in reaction times were observed in the polymerization of the different monomers. It is well-known that steric hindrance of substrates at or near the point of esterification will reduce the rate at which the reaction occurs. Consequently, the reaction durations required to produce high-viscosity polymer melts were extended up to 15 h of *in vacuo* polycondensation. This was specifically observed for poly­(isopropyl methylphloretate) (PiPMC), which has sterically bulky groups on both the alcohol and α-position of the carboxylic acid (Scheme 2).

**Scheme 2:**
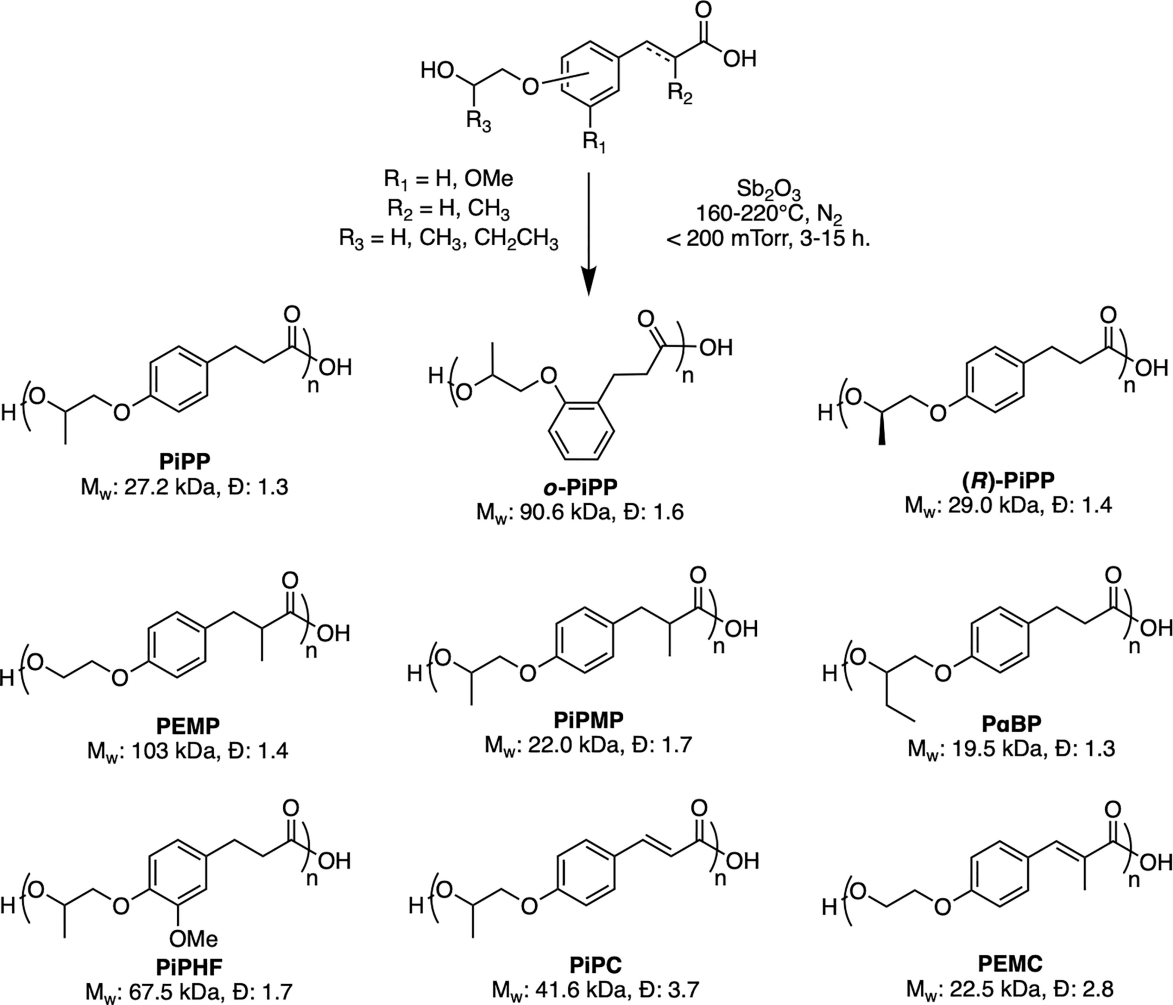
General Synthesis of the Polyesters by Antimony-Catalyzed Melt Polycondensation

### Thermal Analysis

Differential scanning calorimetry (DSC) was used to investigate the thermal transitions of all polyesters (Table 1), which were found to be completely amorphous. Even in the case of isotactic (*R*)-PiPP, polymer melting transitions were never observed via DSC even after multiple attempts at annealing at different temperatures and inducing strain in the sample. The amorphous nature is likely due to the sterically bulky isopropyl, α-butyl, and/or α-methylated groups of the polyesters, which appears to agree with the studies of other lignin-derived polymers.^38,39^ The glass transition temperatures (*T*_g_) ranged from 16–29 °C among the polyesters that had saturated α-β-esters. The incorporation of unsaturated α-β-esters increased the *T*_g_ to 55 °C (PEMC) and 65 °C (PiPC) due to rotational restrictions in the polymeric backbone.

**Table 1:** Summary of Thermal and Mechanical Data Obtained for the Polyesters in This Study, Including Glass Transition Temperature (*T*_g_), Onset of Degradation (*T*_d95%_), Young’s Modulus (E), Elongation at Break (ε_b_), and Yield Stress (σ_*y*_)­^a^

**Polymer**	***T***_**g**_ **(°C)**	***T***_**d95%**_ **(°C)**	**E (MPa)**	**ε**_**b**_ **(%)**	**σ**_**y**_ **(MPa)**
PiPP	25	360	436 ± 32	843 ± 71	1.11 ± 0.07
*o*-PiPP	27	360	864 ± 302	400 ± 45	7.18 ± 0.78
(*R*)-PiPP	25	328	644 ± 230	355 ± 36	1.80 ± 0.63
PEMP	21	415	4.6 ± 0.9	357 ± 37	n.d.
PiPMP	26	371	672 ± 80	886 ± 140	0.63 ± 0.17
PαBP	16	378	7.3 ± 0.3	>3800*	0.18 ± 0.12
PiPHF	29	372	1056 ± 114	188 ± 3.1	6.49 ± 0.06
PEMC	55	374	1200 ± 98	7.5 ± 2.5	10.6 ± 3.6
PiPC	65	363	n.d.	n.d.	n.d.

a “*” – indicates no sample rupture was observed; “n.d.” – indicates that properties could not be determined.

The thermal stability of the polymers was investigated by using thermogravimetric analysis (TGA). The polymers exhibited good thermal stability, with the lowest onset of degradation (5% mass loss) being 328 °C ((*R*)-PiPP) and the highest being 415 °C (PEMP). This high thermal stability aligns well with other reported poly­(ether ester)­s, especially those derived from lignin.^37,38^ Overall, the TGA data indicated that the thermal stability of these materials was sufficient to be processed across a broad range of temperatures through melt extrusion and injection molding.

### Mechanical Characterization

The mechanical performance of polyesters was evaluated through tensile testing of injected molded ASTM-D638 Type V dog bones. The polyesters exhibit significant variation in mechanical properties, ranging from being brittle to extremely ductile. Due to the *T*_g_ of several polyesters being close to room temperature, the environmental conditions were carefully controlled to 25.3 ± 0.3 °C to avoid significant variation in properties. Figure 2 displays the wide array of mechanical properties observed in this study. The highest *T*_g_ polymer, PiPC, was too brittle to test and shattered immediately when attempts were made to secure the sample specimen to the testing machine.

**Figure 2:**
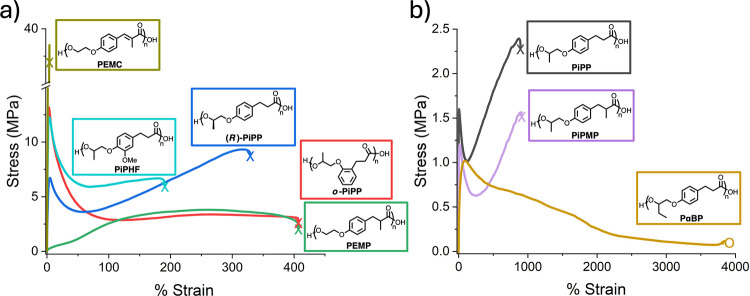
Mechanical properties of polyesters in this study. Representative data is shown for (a) polymers with less than 500% ε_b_ and (b) polymers with greater than 500% ε_b_.

The mechanical performance correlated with the *T*_g_ values of the polyesters. PEMC, with a *T*_g_ of 55 °C, was a stiff polymer with a Young’s modulus (E) of 1200 MPa and 7.5% elongation at break (ε_b_). As the *T*_g_ of the polyesters decreases, the ductility generally improves. PiPHF, with a *T*_g_ of 29 °C, exhibits an E of 1056 MPa and ε_b_ of 188%, making it the highest-modulus polymer among those with α,β-saturated carboxylate groups. This is likely due to the increased rigidity imparted by the methoxy substituent. This trend continues with the decreasing *T*_g_, with a decrease in E and increase in % ε for (*R*)-PiPP, *o*-PiPP, PEMP, PiPP, PiPMP, and PαBP (Table 1 and Figure 2a and 2b). Remarkably, PαBP exhibited a relatively low modulus of 7.3 MPa but had an ε_b_ over 3800%, reaching the travel limit of the tensile tester without sample fracture.

### Gel Content and Rheological Characterization

Melt rheology studies were conducted to explore how the monomer structure influences the inherent properties of the polyesters. Time–temperature superposition (TTS) master curves were constructed for each polyester and properties related to chain bulkiness and stiffness were compared, such as packing length and characteristic ratio. Prior to rheological characterization, the gel content of each polyester was determined via a Soxhlet extraction. Among the synthesized polyesters, only PEMC and PiPC had gel contents of ∼6% and ∼36%, respectively. This was likely the result of cross-linking between α,β-unsaturated carboxylic acids, which is reported in derivatives of coumaric acid and cinnamic acid (Figure 3).^52^

**Figure 3:**
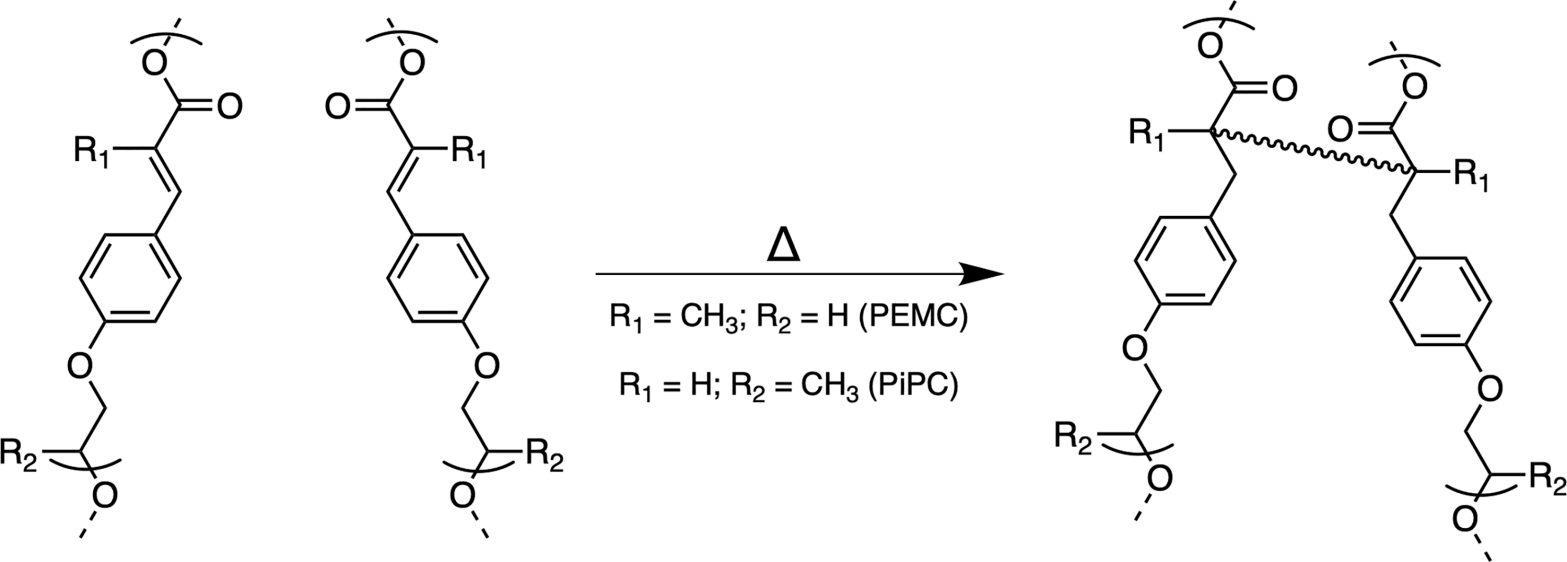
Proposed thermal cross-linking reaction in PEMC and PiPC.

Due to the cross-linked nature of the polymers, the master curves for PEMC and PiPC were challenging to construct and were excluded from further rheological analysis. In contrast, the remaining seven polyesters exhibit crossover points in the TTS master curves (Figures 4 and S3–S8). From the crossover modulus, the plateau modulus (*G* ^0^_N_), entanglement molecular weight (*M*_e_), packing length (*p*), and characteristic ratio (*C*_∞_) can be estimated by using established relationships. The plateau modulus can be estimated by finding the frequency of the loss modulus minimum and the “plateau” that occurs at this point. The storage modulus at the same frequency as the loss modulus minimum is described as the plateau modulus (*G* ^0^_N_ = *G*′(ω)_*G*″◇min_). However, the polymers in this study exhibited little to no minimum loss modulus (Figures 4 and S3–S8), likely due to their relatively low molecular weights.

**Figure 4:**
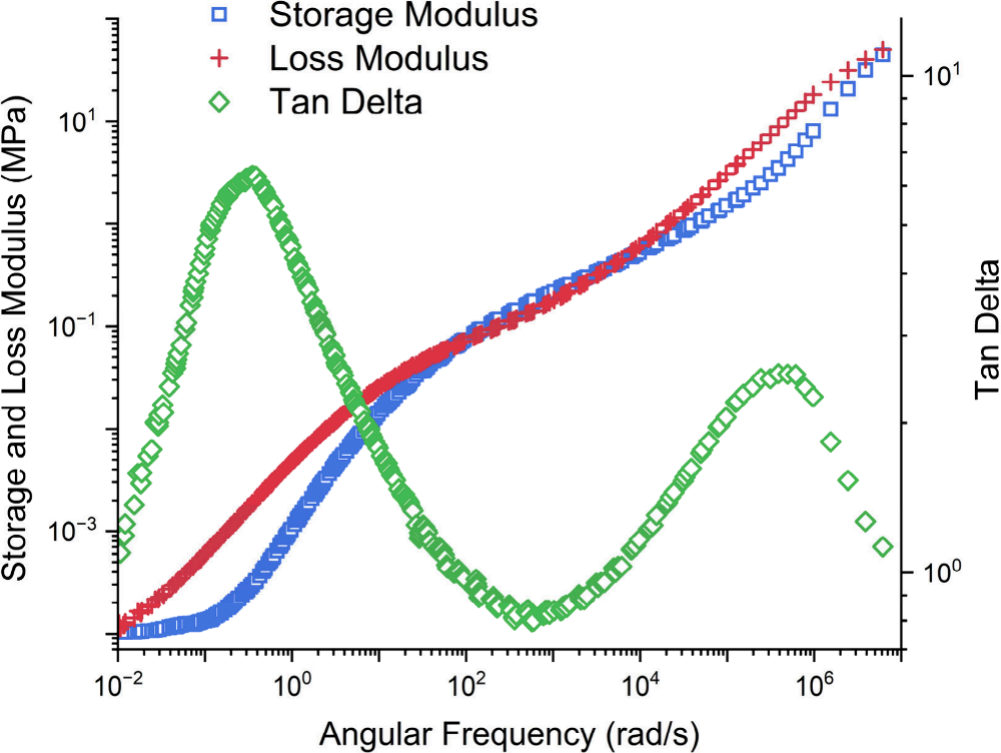
TTS master curve of PiPP.

Instead, the plateau modulus was estimated by using two methods. First, the minimum of the tan­(δ) and the storage modulus at the same frequency (*G* ^0^_N tan(δ)_ = *G*′(ω)_tan(δ)◇min_) was used.^53^ As an additional comparison, the crossover modulus (*G*′ = *G*″ = *G*_C_) was used to solve for the plateau modulus according to the method proposed by Wu (eq 1).^54^ This method relates the crossover modulus and *Đ* to solve for *G* ^0^_N_, which will be referred to as *G* ^0^_N,Wu_.

1 $$\log\left(\frac{G_{N,Wu}^{0}}{G_{C}}\right)=0.380+\frac{2.63\cdot \log\left(Đ\right)}{1+2.45\cdot \log\left(Đ\right)}$$

In 1994, Fetters et al.^55,56^ presented the following relationship between the plateau modulus *G* ^0^_N_ and entanglement molecular weight, *M*_e_^G^ (eq 2):

2 $$G_{N}^{0}=\frac{4\rho RT}{5M_{e}^{G}}$$

From *M*_e_^G^ and the experimentally determined melt density (ρ), the packing length (*p*) can also be determined (eq 3):

3 $$\frac{M_{e}^{G}}{\rho }=218p^{3}$$

The packing length is a measure of chain bulkiness and flexibility and describes how tightly or loosely a polymer can arrange itself and the amount of space (in Å) required by a polymer chain in the melt between other chains. A higher packing length often indicates greater free volume around the polymer chain and is typically associated with a more flexible polymer backbone.

Finally, with the values above, it was possible to calculate the characteristic ratio (*C*_∞_) of the polymers in this study using the following relationship (eq 4):^57^

4 $$C_{\infty }=\frac{10\cdot \rho^{-2/3}\cdot M_{e}^{-1/3}\cdot M_{b}}{L^{2}}$$

Where ρ is the polymer melt density (determined using melt flow index), *M*_b_ is the relative molecular weight per backbone bond, and *L* is the length of each monomer unit (calculated using Chem 3D software).

The *C*_∞_ describes the ratio between the experimental end-to-end length of a polymer and that of the freely joined chain model. A higher *C*_∞_ indicates a stiffer polymer with greater rotational and/or steric hindrances relative to the idealized model. From the equations above, values for *G* ^0^_N_ using Wu’s method (*G* ^0^_N, Wu_) were calculated, as well as values for packing length (*p*) and characteristic ratio (*C*_∞_) (Table 2). Comparisons are made within this polymer class for tacticity, different substituents, structural isomerism, and regioisomerism.

**Table 2:** Summary of Rheological Characteristics Derived and Calculated from the TTS Master Curves

**Name**	***Đ***	**ρ (g/cm** ^**3**^ **)**	***G***_**c**_, **(MPa)**	***G*** ^**0**^_**N, Wu**_ **(MPa)**	***T***_**ref**_ **(°C)**	***M***_**e, Wu**_ **(kDa)**	***p***_**Wu**_ **(Å)**	***C***_**∞, Wu**_
**PiPP**	1.3	1.12	0.065	0.267	80	9.92	3.43	4.30
***o*** **-PiPP**	1.6	1.14	0.038	0.209	80	12.79	3.72	5.28
**(** ***R*** **)-PiPP**	1.4	1.13	0.219	1.007	90	2.71	2.22	6.61
**PEMP**	1.4	1.13	0.085	0.391	80	6.81	3.02	5.61
**PiPMP**	1.7	1.11	0.597	3.493	60	0.71	1.43	11.12
**PαBP**	1.3	1.15	0.182	0.747	60	3.41	2.39	6.43
**PIPHF**	1.7	1.14	0.184	1.074	90	2.57	2.18	7.57

#### Tacticity Effects

Tacticity plays a significant role in the chain flexibility of the polyesters. Previous work by Randall et al. investigated the tacticity effects of PLAs using melt rheology.^57^ Poly­(*meso*-lactide), an atactic PLA that is completely amorphous, had the highest chain flexibility with *C*_∞_ = 5.9, *p* = 3.0 Å. This contrasts with isotactic poly­(*L*-lactide), which exhibited stiffer chains, with *C*_∞_ = 7.5, *p* = 2.3 Å. A similar trend was observed in the case of atactic PiPP (*C*_∞_ = 4.3, *p* = 3.43 Å) compared to isotactic (*R*)-PiPP (*C*_∞_ = 6.6, *p* = 2.22 Å). The decrease in chain flexibility observed from atactic PiPP to isotactic (*R*)-PiPP may correlate with significant differences in the polymer tensile properties. Despite PiPP and (*R*)-PiPP having similar *M*_w_ and *T*_g_, PiPP exhibits more than double the ε_b_ of (*R*)-PiPP.

#### Substituent Effects

The effects of varying pendant groups were also investigated. In a computational study of poly­(propylene), poly­(*n*-butene), poly­(*n*-hexene), and poly­(*n*-octene), based on the rotational isomeric state model, it was predicted that the chain stiffness increases as the side chain length of the polymer increases.^58^ The same trend was observed for PiPP (*C*_∞_ = 4.3, *p* = 3.43 Å) and PαBP (*C*_∞_ = 6.4, *p* = 2.39 Å). However, despite the stiffer chain, PαBP exhibited significantly greater ε_b_ in this study than PiPP, at >3800% compared to 843%. This is likely due to the differences in *T*_g_ between the two polymers. Introducing a bulkier pendant group on the aromatic moiety of the polymer backbone increases chain stiffness, as observed when comparing PiPP (*C*_∞_ = 4.3, *p* = 3.43 Å) to PiPHF (*C*_∞_ = 7.6, *p* = 2.2 Å). This structural change increases the *T*_g_ from 25 °C (PiPP) to 29 °C (PiPHF). The resulting decrease in ε_b_, from 843% (PiPP) to 188% (PiPHF), stems from a combination of higher *T*_g_ and increased chain stiffness.

#### Structural Isomeric Effects

In the case of PEMP and PiPP, the impact of the methyl group position was investigated. The location of the methyl group at the α-position of the carbonyl has a greater effect on the chain stiffness than adjacent to the alcohol, as observed when comparing PEMP (*C*_∞_ = 5.6, *p* = 3.02 Å) and PiPP (*C*_∞_ = 4.3, *p* = 3.43 Å). Although the *T*_g_ of PEMP (21 °C) is lower than that of PiPP (25 °C), it still exhibited reduced ε_b_ at 350% (PEMP) relative to 843% (PiPP), possibly due to the increased chain stiffness of the polyester. Additionally, structural isomers PiPMP and PαBP were analyzed. The presence of two methyl groups in PiPMP (C_∞_ = 11.1, *p* = 1.43 Å) appear to have a more significant impact on the stiffness of the polymer chain than the ethyl pendant group of PαBP (*C*_∞_ = 6.4, *p* = 2.39 Å). The increase in chain stiffness may correspond to the increased *T*_g_ of PiPMP (26 °C) and subsequent reduction in ε_b_ (886%) when compared to PαBP (*T*_g_: 16 °C, ε_b_: > 3800%).

#### Regioisomeric Effects

It was observed that regioisomerism has the least significant effect when comparing the rheological properties of this polymer series. The difference in chain stiffness between *o*-PiPP (*C*_∞_ = 5.3, *p* = 3.72 Å) and PiPP (*C*_∞_ = 4.3, *p* = 3.43 Å) is present, but to a lesser degree than previous examples. However, even with the slight increase in chain stiffness, the *T*_g_ of *o*-PiPP is increased to 27 °C and likely contributes to the decrease in ε_b_ from 843% (PiPP) to 400% (*o*-PiPP).

### Chemical Recycling

Previously, we demonstrated that PiPP and other amorphous polyesters can degrade under industrial composting conditions.^38^ We have also observed in unreported work that these polyesters are chemically recyclable via base hydrolysis. In contrast to semiaromatic, AABB-type polyesters such as PET, PBT, and PEF, these lignin-derived AB-type polyesters do not contain water-soluble and volatile glycols in the polymeric backbone. Because of this, chemically recycling the polymers in aqueous conditions is straightforward and can be accomplished in high yields.

For this study, (*R*)-PiPP was chosen as a model polyester, although this strategy may be applied to other polyesters in this series. The polymer (Figure 5b, *M*_w_: 27.0 kDa, *Đ* = 1.4) was degraded rapidly at 150 °C by using a sealed system in 2 M KOH in ethanol and water. The recycled monomer (Figure 5c) exhibited excellent purity via ^1^H NMR with a comparable spectrum to the starting monomer (Figure 5a). Matching the reaction times between the two batches resulted in a slightly higher molecular weight of the second batch, with *M*_w_ = 42.3 kDa; *Đ*: 1.5 (Figure 5d). Although the sealed pressure vessel is not a necessity, it enables higher temperatures that significantly accelerate depolymerization, resulting in 80% monomer yield after just 60 min. As a qualitative point of comparison, both PiPP and *o*-PiPP have been chemically recycled under ambient pressure, requiring approximately 12 h to achieve comparable monomer recovery. These results demonstrate that chemical recycling to monomer is a viable end-of-life strategy for this polymer series.

**Figure 5:**
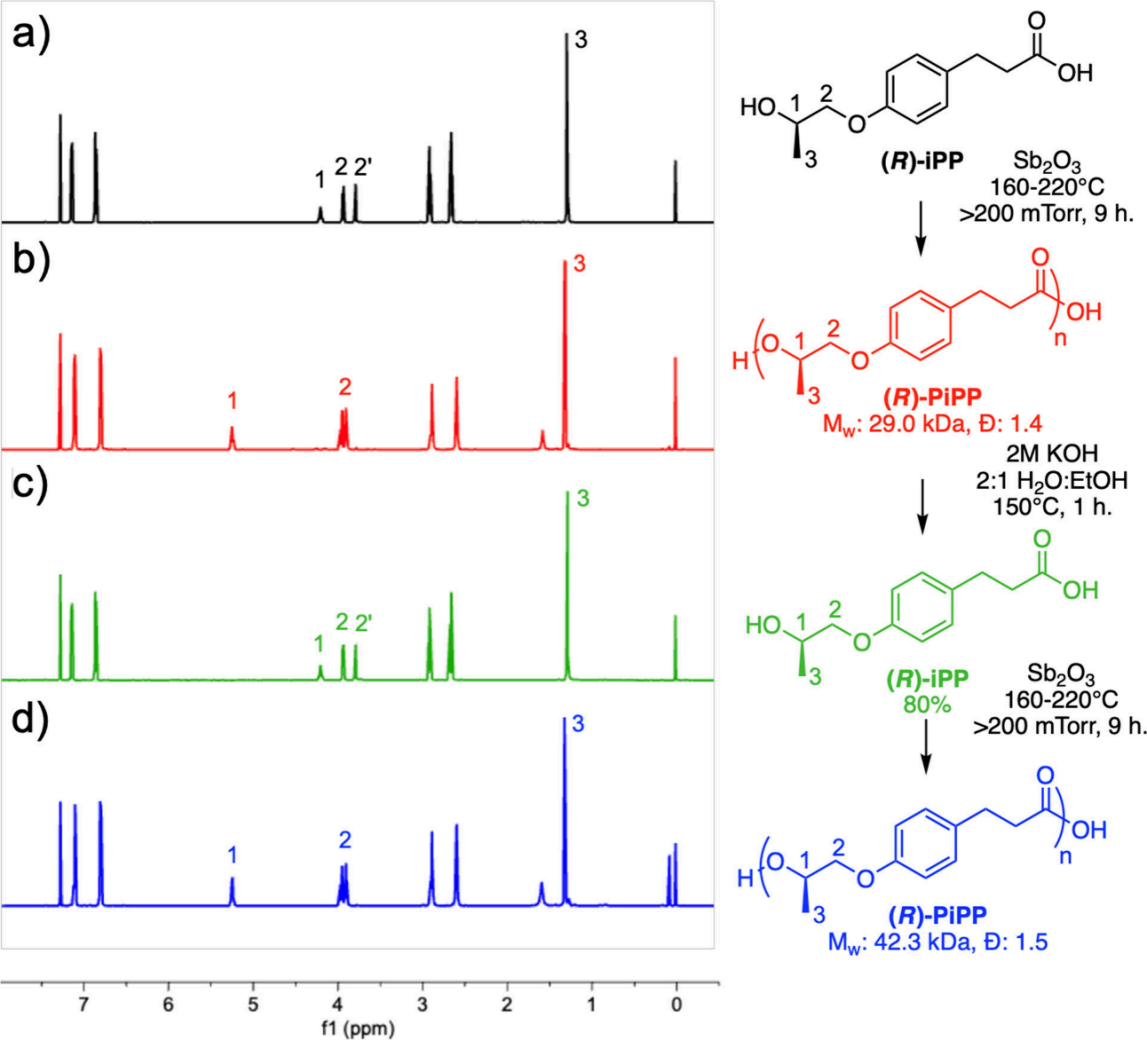
^1^H NMR spectra of (*R*)-iPP monomer (a), (*R*)-PiPP polymer (b), recycled (*R*)-iPP monomer (c), and repolymerized (*R*)-PiPP (d) in chloroform-*d*.

## Conclusions

In this study, nine potentially lignin-derived monomers were synthesized using sustainable, high-yield reactions that were easily scalable to produce polymers at greater than 50 g scale. Comprehensive characterization of thermal and mechanical properties revealed excellent thermal stability, with *T*_g_ ranging from 16 to 63 °C. Mechanical properties ranged from polymers that were too brittle to test to those with strain at break exceeding 3800%. Through iterative monomer design, rheological properties, of subsequent polymers were evaluated using TTS master curves, demonstrating a diverse range of polymer properties, with packing lengths varying from 1.43 to 3.43 Å and *C*_∞_ ranging from 4.30 to 11.12. Finally, using (*R*)-PiPP as a case study, it was demonstrated that these polyesters can be efficiently depolymerized to a monomer via base hydrolysis, achieving rapid conversion with high yield. Subsequent repolymerization resulted in polymers with equal or greater molecular weights compared to the initial polyester.

## Supplementary Material



## Notes

Synthetic procedures for monomers and polyesters, TGA curves, DSC curves, processing conditions, TTS master curves, ^1^H NMR data, ^13^C NMR data, and GPC data (PDF)

## References

[1] MacLeod M., Arp H. P. H., Tekman M. B., Jahnke A. (2021). The global threat from
plastic pollution. Science.

[2] Geyer R., Jambeck J. R., Law K. L. (2017). Production,
use, and fate of all
plastics ever made. Science Advances.

[3] Zhang Q., Song M., Xu Y., Wang W., Wang Z., Zhang L. (2021). Bio-based polyesters:
Recent progress and future prospects. Prog.
Polym. Sci..

[4] Cywar R. M., Rorrer N. A., Hoyt C. B., Beckham G. T., Chen E. Y. X. (2022). Bio-based
polymers with performance-advantaged properties. Nature Reviews Materials.

[5] Vidal F., van der Marel E. R., Kerr R. W. F., McElroy C., Schroeder N., Mitchell C., Rosetto G., Chen T. T. D., Bailey R. M., Hepburn C. (2024). Designing a circular carbon and plastics economy for
a sustainable future. Nature.

[6] Miller S. A. (2013). Sustainable
Polymers: Opportunities for the Next Decade. ACS Macro Lett..

[7] Reddy C.S.K, Ghai R, Rashmi, Kalia V.C (2003). Polyhydroxyalkanoates: an overview. Bioresource technology.

[8] Swetha T. A., Bora A., Mohanrasu K., Balaji P., Raja R., Ponnuchamy K., Muthusamy G., Arun A. (2023). A comprehensive review
on polylactic acid (PLA) - Synthesis, processing and application in
food packaging. Int. J. Biol. Macromol..

[9] Aliotta L., Seggiani M., Lazzeri A., Gigante V., Cinelli P. (2022). A Brief Review
of Poly (Butylene Succinate) (PBS) and Its Main Copolymers: Synthesis,
Blends, Composites, Biodegradability, and Applications. Polymers (Basel).

[10] Jian J., Xiangbin Z., Xianbo H. (2020). An overview on synthesis, properties
and applications of poly­(butylene-adipate-co-terephthalate)–PBAT. Advanced Industrial and Engineering Polymer Research.

[11] Loos K., Zhang R., Pereira I., Agostinho B., Hu H., Maniar D., Sbirrazzuoli N., Silvestre A. J., Guigo N., Sousa A. F. (2020). A perspective on PEF synthesis, properties,
and end-life. Frontiers in chemistry.

[12] Lund F., Gorwa-Grauslund M. (2024). Production
of bio-based adipic acid using a combination
of engineered Pseudomonas putida strains. Sustainable
Chemistry for the Environment.

[13] Toray Invents 100% Bio-Based Adipic Acid from Sugars Derived from Inedible Biomass, Scaling Up for Application to Eco-Friendly Nylon 66. Toray Industries, 2022 https://www.plastics.toray/zh/news/article.html?contentId=k3kq0scj (Accessed 4.11.2024)

[14] Burk, M. J. ; Van Dien, S. J. ; Burgard, A. P. ; Niu, W. Compositions and methods for the biosynthesis of 1,4-butanediol and its precursors. U.S. Patent US8,067,214B2 2011.

[15] He Y., Luo Y., Yang M., Zhang Y., Zhu L., Fan M., Li Q. (2022). Selective catalytic synthesis of bio-based terephthalic
acid from
lignocellulose biomass. Applied Catalysis A:
General.

[16] Tachibana Y., Kimura S., Kasuya K.-i. (2015). Synthesis and Verification of Biobased
Terephthalic Acid from Furfural. Sci. Rep..

[17] Volanti M., Cespi D., Passarini F., Neri E., Cavani F., Mizsey P., Fozer D. (2019). Terephthalic
acid from renewable
sources: early-stage sustainability analysis of a bio-PET precursor. Green Chem..

[18] Delidovich I., Hausoul P. J. C., Deng L., Pfützenreuter R., Rose M., Palkovits R. (2016). Alternative Monomers Based on Lignocellulose
and Their Use for Polymer Production. Chem.
Rev..

[19] Vermaas J. V., Crowley M. F., Beckham G. T. (2020). Molecular Lignin
Solubility and Structure
in Organic Solvents. ACS Sus. Chem. Eng..

[20] Sun Z., Fridrich B., De Santi A., Elangovan S., Barta K. (2018). Bright side of lignin
depolymerization: toward new platform chemicals. Chem. Rev..

[21] Pollegioni L., Tonin F., Rosini E. (2015). Lignin-degrading enzymes. Febs j.

[22] Fang, Z. ; Smith, J. R. L. Production of Biofuels and Chemicals from Lignin, 1st ed.; Springer: Singapore; Imprint: Springer, 2016.

[23] Zhong R., Cui D., Ye Z.-H. (2019). Secondary cell wall biosynthesis. New Phytologist.

[24] Martínková L., Grulich M., Pátek M., Křístková B., Winkler M. (2023). Bio-Based Valorization of Lignin-Derived Phenolic Compounds:
A Review. Biomolecules.

[25] Qiang H., Wang J., Liu H., Zhu Y. (2023). From vanillin to biobased
aromatic polymers. Polym. Chem..

[26] Libretti C., Santos Correa L., Meier M. A. R. (2024). From waste to resource: advancements
in sustainable lignin modification. Green Chem..

[27] Andriani F., Lawoko M. (2024). Oxidative Carboxylation
of Lignin: Exploring Reactivity
of Different Lignin Types. Biomacromolecules.

[28] Subbotina E., Souza L. R., Zimmerman J., Anastas P. (2024). Room temperature catalytic
upgrading of unpurified lignin depolymerization oil into bisphenols
and butene-2. Nat. Commun..

[29] Hafezisefat P., Qi L., Brown R. C. (2023). Lignin
Depolymerization and Esterification with Carboxylic
Acids to Produce Phenyl Esters. ACS Sus. Chem.
Eng..

[30] Upton B. M., Kasko A. M. (2016). Strategies for the Conversion of
Lignin to High-Value
Polymeric Materials: Review and Perspective. Chem. Rev..

[31] Tana T., Zhang Z., Beltramini J., Zhu H., Ostrikov K. K., Bartley J., Doherty W. (2019). Valorization of native
sugarcane
bagasse lignin to bio-aromatic esters/monomers via a one pot oxidation–hydrogenation
process. Green Chem..

[32] Kim S., Chung H. (2024). Biodegradable Polymer:
From Synthesis Methods to Applications of
Lignin-graft-Polyester. Green Chem..

[33] Duval A., Benali W., Avérous L. (2024). Turning lignin
into a recyclable
bioresource: transesterification vitrimers from lignins modified with
ethylene carbonate. Green Chem..

[34] Bilal M., Qamar S. A., Qamar M., Yadav V., Taherzadeh M. J., Lam S. S., Iqbal H. M. N. (2024). Bioprospecting
lignin biomass into
environmentally friendly polymers—Applied perspective to reconcile
sustainable circular bioeconomy. Biomass Conversion
and Biorefinery.

[35] Huang J., Wang H., Liu W., Huang J., Yang D., Qiu X., Zhao L., Hu F., Feng Y. (2023). Solvent-free synthesis
of high-performance polyurethane elastomer based on low-molecular-weight
alkali lignin. Int. J. Biol. Macromol..

[36] Ana, L. ; Helena, P. Compositional Variability of Lignin in Biomass. In Lignin; Matheus, P. , Ed.; IntechOpen, 2017; p Ch. 3.

[37] Mialon L., Vanderhenst R., Pemba A. G., Miller S. A. (2011). Polyalkylenehydroxybenzoates
(PAHBs): Biorenewable aromatic/aliphatic polyesters from lignin. Macromol. Rapid Commun..

[38] Winfield D., Ring J., Horn J., White E. M., Locklin J. (2021). Semi-aromatic
biobased polyesters derived from lignin and cyclic carbonates. Green Chem..

[39] Xanthopoulou E., Terzopoulou Z., Zamboulis A., Koltsakidis S., Tzetzis D., Peponaki K., Vlassopoulos D., Guigo N., Bikiaris D. N., Papageorgiou G. Z. (2023). Poly­(hexylene
vanillate): Synthetic Pathway and Remarkable Properties of a Novel
Alipharomatic Lignin-Based Polyester. ACS Sus.
Chem. Eng..

[40] Grossman A., Vermerris W. (2019). Lignin-based polymers and nanomaterials. Curr. Opin. Biotechnol..

[41] Dessbesell L., Paleologou M., Leitch M., Pulkki R., Xu C. C. (2020). Global
lignin supply overview and kraft lignin potential as an alternative
for petroleum-based polymers. Renewable and
Sustainable Energy Reviews.

[42] Alinejad M., Henry C., Nikafshar S., Gondaliya A., Bagheri S., Chen N., Singh S. K., Hodge D. B., Nejad M. (2019). Lignin-based polyurethanes: Opportunities
for bio-based foams, elastomers,
coatings and adhesives. Polymers.

[43] Laurichesse S., Avérous L. (2014). Chemical modification
of lignins: Towards biobased
polymers. Prog. Polym. Sci..

[44] Llevot A., Grau E., Carlotti S., Grelier S., Cramail H. (2016). From lignin-derived
aromatic compounds to novel biobased polymers. Macromol. Rapid Commun..

[45] Duval A., Lawoko M. (2014). A review on lignin-based
polymeric, micro-and nano-structured
materials. React. Funct. Polym..

[46] Pescarmona P. P. (2021). Cyclic
carbonates synthesised from CO2: Applications, challenges and recent
research trends. Current Opinion in Green and
Sustainable Chemistry.

[47] Anastas, P. T. ; Warner, J. C. Green chemistry: theory and practice; Oxford University Press, 2000.

[48] Standard Test Method for Tensile Properties of Plastics; ASTM D638-10; American Society for Testing and Materials, 2015.

[49] Karlen S., Fasahati P., Mazaheri M., Serate J., Smith R., Sirobhushanam S., Chen M., Tymokhin V., Cass C., Liu S. (2020). Assessing the Viability of Recovery of Hydroxycinnamic
Acids from Lignocellulosic Biorefinery Alkaline Pretreatment Waste
Streams. ChemSusChem.

[50] Khayrani A. C., Hidayatullah I. M., Satyawan I. L., Riyadi F. A., Sambudi N. S., Manas N. H. A. (2024). Techno-Economic
Assessment of Ferulic Acid Bioproduction
from Agro-industrial Waste Using Aspergillus niger. Waste and Biomass Valorization.

[51] Papadopoulos L., Zamboulis A., Kasmi N., Wahbi M., Nannou C., Lambropoulou D. A., Kostoglou M., Papageorgiou G. Z., Bikiaris D. N. (2021). Investigation of
the catalytic activity and reaction
kinetic modeling of two antimony catalysts in the synthesis of poly­(ethylene
furanoate). Green Chem..

[52] Sung S.-J., Cho K.-Y., Hah H., Lee J., Shim H.-K., Park J.-K. (2006). Two different reaction mechanisms
of cinnamate side
groups attached to the various polymer backbones. Polymer.

[53] Liu C., He J., Ruymbeke E. v., Keunings R., Bailly C. (2006). Evaluation
of different
methods for the determination of the plateau modulus and the entanglement
molecular weight. Polymer.

[54] Wu S. (1989). Chain structure
and entanglement. J. Polym. Sci., Part B: Polym.
Phys..

[55] Larson R. G., Sridhar T., Leal L. G., McKinley G. H., Likhtman A. E., McLeish T. C. B. (2003). Definitions of entanglement spacing
and time constants
in the tube model. J. Rheol..

[56] Fetters L. J., Lohse D. J., Richter D., Witten T. A., Zirkel A. (1994). Connection
between Polymer Molecular Weight, Density, Chain Dimensions, and Melt
Viscoelastic Properties. Macromolecules.

[57] Randall J., Flodquist M., Schroeder J., Valentine J. R., Owusu O., McCarthy K., Weed J., Hennen J., Heuzey M. C., Carreau P. J. (2024). Comparing the Structural, Thermal,
and Rheological Properties of Poly­(meso-lactide) to Poly­(l-lactide)
and Poly­(rac-lactide). Macromolecules.

[58] Madkour T. M., Goderis B., Mathot V. B., Reynaers H. (2002). Influence of chain
microstructure on the conformational behavior of ethylene-1-olefin
copolymers. Impact of the comonomeric mole content and the catalytic
inversion ratio. Polymer.

