# Supplementary material for: Structure–Property Relationships of Lignin-Derived Semiaromatic Poly(ether ester)s
Source: Macromolecules. 2025 Jul 11;58(14):7457–66. doi: 10.1021/acs.macromol.5c00380 (PMC12288064; doi:10.1021/acs.macromol.5c00380)
Supplement: Supplementary file 1 [file ma5c00380_si_001.pdf]

Supporting Information: Structure-Property Relationships of Lignin-Derived Semi-Aromatic Poly(ether ester)s.

Authors: Ryan K. Maynard[†,§], Kush G. Patel[‡,§], Huiming Wu[†,§], Frida C. Knudsen[†,§], Imrie C. Ross[†,§], DeMichael D. Winfield[†,§] and Jason J. Locklin[†,‡,§,*]

† Department of Chemistry, Franklin College of Arts and Sciences, University of Georgia, 140 Cedar Street, Athens, Georgia, 30602, United States.

‡ School of Chemical, Materials, and Biomedical Engineering, College of Engineering, University of Georgia, 597 D.W. Brooks Drive, Athens, Georgia, 30602, United States.

§ New Materials Institute, University of Georgia, 220 Riverbend Road, Athens, Georgia, 30602, United States.

* Corresponding Author – email: jlocklin@uga.edu

Table of Contents:

Synthesis of Hydroxyacids, Hydroxyesters, and Monomers: 4

Synthesis of methyl 3-(2-hydroxyphenyl)propanoate: 4

Synthesis of 3-(4-hydroxyphenyl)-2-methylacrylic acid: 4

Synthesis of methyl 3-(4-hydroxyphenyl)-2-methylacrylate: 5

Synthesis of 3-(4-hydroxyphenyl)-2-methylpropanoic acid: 6

Synthesis of methyl 3-(4-hydroxyphenyl)-2-methylpropanoate: 7

General synthesis for alkylation of phenols from ring-opening of cyclic carbonates: 8

Synthesis of 3-(2-(2-hydroxypropoxy)phenyl)propanoic acid: 9

Synthesis of (R)-3-(4-(2-hydroxypropoxy)phenyl)propanoic acid: 9

Synthesis of 3-(4-(2-hydroxyethoxy)phenyl)-2-methylpropanoic acid: 10

Synthesis of 3-(4-(2-hydroxypropoxy)phenyl)-2-methylpropanoic acid: 11

Synthesis of 3-(4-(2-hydroxybutoxy)phenyl)propanoic acid: 11

Synthesis of 3-(4-(2-hydroxypropoxy)-3-methoxyphenyl)propanoic acid: 12

Synthesis of (E)-3-(4-(2-hydroxyethoxy)phenyl)-2-methylacrylic acid: 13

Synthesis of 3-(4-(2-hydroxypropoxy)phenyl)acrylic acid: 13

Polyester Synthesis: 15

Synthesis of poly-isopropyl phloretate (PiPP): 15

Synthesis of poly-ortho-isopropyl phloretate (o-PiPP): 15

Synthesis of (R)-poly-isopropyl phloretate ((R)-PiPP): 16

Synthesis of poly-ethylene methyl phloretate (PEMP): 16

Synthesis of poly-isopropyl methyl phloretate (PiPMP): 17

Synthesis of poly-α-butylene phloretate (PαBP): 17

Synthesis of poly-isopropyl dihydroferulate (PiPHF): 18

Synthesis of poly-ethylene methyl cinnamate (PEMC): 19

Synthesis of poly-isopropyl cinnamate (PiPC): 19

Thermal Characterization of Polymers 21

Figure S1. TGA overlay 21

Figure S2. DSC overlay 21

Injection Molding of Polymers 22

Table S1. Summary of extrusion and injection molding conditions 22

Rheological Characterization of Polyesters: 23

Figure S3. TTS master curve of o-PiPP. 23

Figure S4. TTS master curve of (R)-PiPP. 23

Figure S5. TTS master curve of PEMP. 24
Figure S6. TTS master curve of PiPMP. 24
Figure S7. TTS master curve of PαBP. 25
Figure S8. TTS master curve of PiPHF 25
^{1}H NMR Spectra of Synthesized Precursors, Monomers, and Polymers 26
^{13}C NMR Spectra of Monomers and Polymers 46
GPC Data 62

Synthesis of Hydroxyacids, Hydroxyesters, and Monomers:

Synthesis of methyl 3-(2-hydroxyphenyl)propanoate:

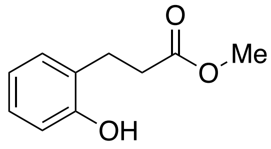

100 g of dihydrocoumarin (665 mmol), 1 L of methanol, and 1 mL of concentrated sulfuric acid were added to a 2 L, single neck flask. The solution was heated to reflux for 12 h. before being cooled to room temperature. The solvent was removed under reduced pressure and the residue was taken up into ethyl acetate. The organic layer was washed with water, a saturated solution of sodium bicarbonate, then brine, followed by drying over magnesium sulfate. After filtration, the solvent was removed under reduced pressure to afford 116 g of a slowly crystallizing white solid (95%). The resulting ^{1}H NMR spectrum agreed with previously published results.[1] ^{1}H NMR (600 MHz, $CDCl_3$): δ 7.11 (m, 2H, H_{Ar}), 7.01 (s, 1H, OH), 6.87 (t, 2H, H_{Ar}), 3.69 (s, 3H, OCH_3), 2.91 (t, 2H, CH_2), 2.73 (t, 2H, CH_2).

Synthesis of 3-(4-hydroxyphenyl)-2-methylacrylic acid:

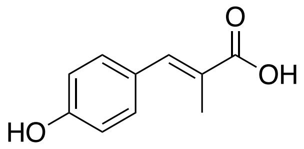

Adapted from published procedure.[2] 260 g of 4-hydroxybenzaldehyde (2.13 mol) and 435 g of sodium propionate (4.5 mol) were added to a 5 L 3-neck flask. The flask was purged with full

vacuum and backfilled with dry nitrogen three times. 790 mL of propionic anhydride (6 mol) were added under positive nitrogen flow and the mixture was heated to 140 °C, becoming a homogenous, light brown solution. The solution was kept at this temperature for 48 h, during which it became extremely dark in color. After this, the solution was cooled to room temperature and 3 L of deionized water were added. The heterogenous mixture was filtered, and the cake was washed with water. The collected solid was dissolved in 3 L of 2 M NaOH solution and filtered once again. The small amount of collected solid was discarded, and the filtrate was acidified to pH = 1. A final filtration was conducted, and the collected solid was once again washed very thoroughly with water. The solid was dried under vacuum at 60 °C for 24 h. to yield 370 g of a light tan solid (98%). Spectra agreed with the previously published result. ^{1}H NMR (600 MHz, DMSO-d_6): δ 12.27 (s, 1H, COO*H*), 9.89 (s, 1H, O*H*), 7.50 (s, 1H, =C*H*), 7.34 (d, 2H, H_{Ar}), 6.83 (d, 2H, H_{Ar}), 2.02 (s, 3H, CH_3).

Synthesis of methyl 3-(4-hydroxyphenyl)-2-methylacrylate:

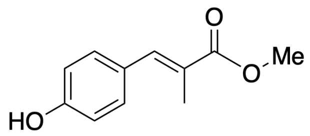

120 g (0.67 mol) of 3-(4-hydroxyphenyl)-2-methylacrylic acid, 1 L methanol, and 1 mL concentrated H_2SO_4 were added to a 2 L single neck flask and refluxed for 14 hours. After completion of the reaction, the solvent was removed under reduced pressure and the residue was taken up in ethyl acetate. The organic layer was washed once with water, three times with saturated aqueous sodium bicarbonate solution, and once with brine. The organic layer was dried over magnesium sulfate and then filtered into a flask. Excess solvent was removed under reduced

pressure to afford 127 g of product as a light tan solid (98%). ^{1}H NMR (600 MHz, DMSO-d_6): δ 9.86 (s, 1H, O*H*), 7.53 (s, 1H, =C*H*), 7.36 (d, 2H, H_{Ar}), 6.82 (d, 2H, H_{Ar}), 3.72 (s, 3H, OCH_3), 2.06, (s, 3H, CH_3).

Synthesis of 3-(4-hydroxyphenyl)-2-methylpropanoic acid:

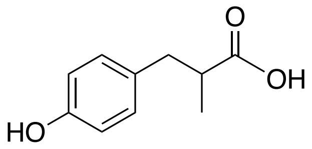

134 g of 3-(4-hydroxyphenyl)-2-methylacrylic acid (752 mmol), 7.98 g of 10 % pd/C (0.01 eq.), and 1.5 L of ethyl acetate were added to a 3 L three-necked flask under a nitrogen atmosphere. The atmosphere of the flask was gently evacuated and backfilled 5 times with hydrogen. The mixture was kept under a hydrogen atmosphere for 48 h and monitored by ^{1}H NMR spectroscopy. After this time, complete conversion was not observed. The atmosphere was flushed with nitrogen and a second equivalent of catalyst (7.98 g) was added. Once again, the atmosphere was gently evacuated and backfilled 5 times with hydrogen. The mixture was stirred in a hydrogen atmosphere for another 48 hours until ^{1}H NMR spectroscopy indicated complete conversion. The solid catalyst was removed via filtration, and the filtrate was further purified by passing through a short column of celite. The solvent was removed under reduced pressure to yield 120 g of a slowly crystallizing yellow solid (90%). ^{1}H NMR (600 MHz, DMSO-d_6): δ 12.07 (s, 1H, COO*H*), 9.16 (s, 1H, s, O*H*), 6.96 (d, 2H, H_{Ar}), 6.66 (d, 2H, H_{Ar}), 2.79 (q, 1H, CH*H*), 2.54 (m, 1H, CH*H*), 2.47 (m, 1H, C*H*), 1.01 (d, 3H, CH_3).

Synthesis of methyl 3-(4-hydroxyphenyl)-2-methylpropanoate:

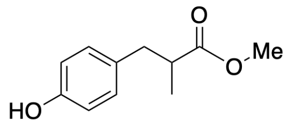

120 g (0.67 mol) of 3-(4-hydroxyphenyl)-2-methylpropanoic acid, 1 L methanol, and 1 mL concentrated H_2SO_4 were added to a 2 L single neck flask and refluxed under air for 16 hours. After completion of the reaction, the solvent was removed under reduced pressure and the residue was taken up in ethyl acetate. The organic layer was washed once with water, three times with saturated aqueous sodium bicarbonate solution, and once with brine. The organic layer was dried over magnesium sulfate and then filtered into a flask. Excess solvent was removed under reduced pressure to afford 124 g of product as a brown, slowly-crystallizing solid (95%). ^{1}H NMR (600 MHz, DMSO-d_6): δ 9.18 (s, 1H, O*H*), 6.94 (d, 2H, H_{Ar}), 6.65 (d, 2H, H_{Ar}), 3.54 (s, 3H, OCH_3), 2.76 (q, 1H, C*H*), 2.64 (m, 1H, C*H*), 2.53 (q, 1H, C*H*), 1.02 (d, 3H, CH_3).

General synthesis for alkylation of phenols from ring-opening of cyclic carbonates:

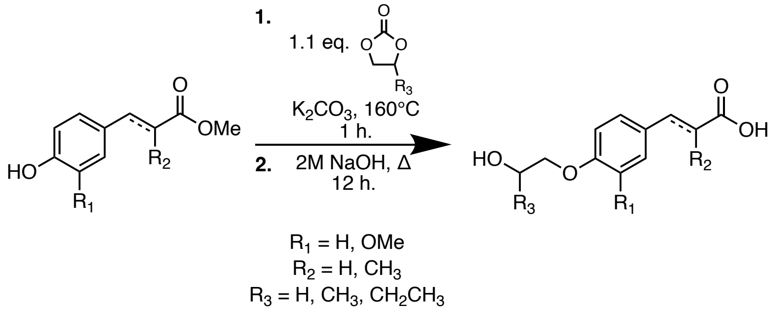

The procedure outlined in our previous work was followed. 55-90 g of starting phenol were added to a 1 L 3-neck flask. 0.05 eq. of anhydrous potassium carbonate was added to the flask, which was purged under full vacuum and backfilled with nitrogen three times. 1.1 eq. of the appropriate cyclic carbonate was added under positive nitrogen pressure, and the entire flask was heated to 160 °C while stirring for 90 minutes. During this time, the mixture increased in viscosity and significant quantities of carbon dioxide evolved. After the reaction was complete (as evidenced in a lack of carbon dioxide evolution), the mixture was cooled to roughly 40 °C upon which 500 mL of 3 M NaOH were added. The solution was heated to reflux until the entire solution became homogenous, usually between 12-48 h. After the solution became homogenous, it was cooled to room temperature and acidified carefully with 6M HCl until the pH = 2. The precipitate was filtered, and the filter cake was washed thoroughly with acidified water (pH = 2) and dried in a vacuum oven at 60°C for 24 h. to afford the products in good to excellent yields.

Synthesis of 3-(2-(2-hydroxypropoxy)phenyl)propanoic acid:

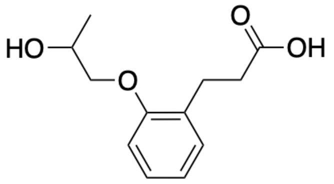

90 g of methyl 3-(2-hydroxyphenyl)propanoate (0.5 mol) were added to a 1 L 3-neck flask with 56.1 g of propylene carbonate (1.1 eq.). The general synthesis procedure outlined above was followed and after alkylation the mixture was refluxed in 500 mL 3 M NaOH for 48 h. An undissolved precipitate was removed by filtration. Acidification and filtration as described above yielded 87.9 g of a white solid (78%). ^{1}H NMR (600 MHz, $CDCl_3$): δ 7.17 (m, 2H, H_{Ar}), 6.90 (t, 1H, H_{Ar}), 6.81 (d, 1H, H_{Ar}), 4.24 (m, 1H, C*H*), 3.96 (dd, 1H, CH*H*), 3.81 (dd, 1H, CH*H*), 3.00 (m, 2H, CH_2), 2.63 (m, 2H, CH_2), 1.30 (d, 3H, CH_3). ^{13}C NMR (400 MHz, DMSO-*d6*): 174.01, 156.41, 129.50, 128.81, 127.39, 120.16, 111.42, 73.05, 64.59, 33.66, 25.41, 20.23.

Synthesis of (R)-3-(4-(2-hydroxypropoxy)phenyl)propanoic acid:

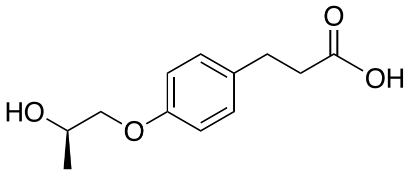

90 g of methyl 3-(4-hydroxyphenyl)propanoate (0.5 mol) was synthesized as previously described[3] and added to a 1 L 3-neck flask with 56.1 g of (R)-propylene carbonate (1.1 eq.). The general synthesis procedure outlined above was followed and after alkylation the mixture was refluxed in 500 mL 3 M NaOH for 48 h. Acidification and filtration as described above yielded an impure solid that was crystallized from water to yield 98.0 g of a white powder (87%). ^{1}H NMR (600 MHz, $CDCl_3$): δ 7.11 (d, 2H, H_{Ar}), 6.84 (d, 2H, H_{Ar}), 4.18 (m, 1H, C*H*), 3.91 (dd, 1H,

C*H*H), 3.77 (dd, 1H, CH*H*), 2.90 (t, 2H, CH_2), 2.65 (t, 2H, CH_2), 1.28 (d, 3H, CH_3). ^{13}C NMR (400 MHz, DMSO-*d6*): 173.74, 156.99, 132.70, 129.13, 115.52, 114.32, 73.18, 64.49, 35.55, 29.47, 20.13.

Synthesis of 3-(4-(2-hydroxyethoxy)phenyl)-2-methylpropanoic acid:

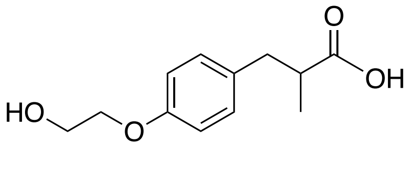

60 g of methyl 3-(4-hydroxyphenyl)-2-methylpropanoate (0.31 mol) were added to a 1 L 3-neck flask with 29.9 g of ethylene carbonate (1.1 eq.). The general synthesis procedure outlined above was followed and after alkylation the mixture was refluxed in 500 mL 3 M NaOH for 24 h. The aqueous solution was added to a 2 L separatory funnel and slowly acidified with 2 M HCl until pH = 2. The aqueous layer was extracted three times with ethyl acetate, and the combined organic fractions were washed with brine, dried over magnesium sulfate, filtered into a flask, and the solvent removed with rotary evaporation. The color of the product was unexpectedly dark, so it was further purified by dissolving in 500 mL dichloromethane and spinning over activated charcoal for 2 h. The charcoal was removed via filtration over a short pad of celite and the solvent was removed via rotary evaporation. 58.9 g of a slowly crystallizing yellow solid were collected (85%). ^{1}H NMR (600 MHz, $CDCl_3$): δ 7.09 (d, 2H, H_{Ar}), 6.83 (d, 2H, H_{Ar}), 4.05 (t, 2H, CH_2), 3.95 (t, 2H, CH_2), 2.98 (q, 1H, CH*H*), 2.71 (m, 1H, C*H*), 2.64 (q, 1H, CH*H*), 1.17 (d, 3H, CH_3). ^{13}C NMR (400 MHz, DMSO-*d6*): 176.82, 157.05, 131.33, 129.80, 114.16, 69.37, 59.58, 40.76, 38.07, 16.55.

Synthesis of 3-(4-(2-hydroxypropoxy)phenyl)-2-methylpropanoic acid:

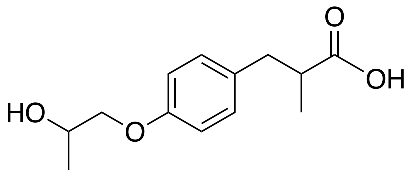

60 g of methyl 3-(4-hydroxyphenyl)-2-methylpropanoate (0.31 mol) were added to a 1 L 3-neck flask with 34.7 g of propylene carbonate (1.1 eq.). The general synthesis procedure outlined above was followed and after alkylation the mixture was refluxed in 500 mL 3M NaOH for 24 h. The aqueous solution was added to a 2 L separatory funnel and acidified with 2 M HCl until pH = 2. The aqueous layer was extracted three times with ethyl acetate, and the combined organic fractions were washed with brine, dried over magnesium sulfate, filtered into a flask, and the solvent removed with rotary evaporation to isolate 68.0 g of a light brown, viscous liquid (92%). ^{1}H NMR (600 MHz, $CDCl_3$): δ 7.10 (d, 2H, H_{Ar}), 6.83 (d, 2H, H_{Ar}), 5.30 (s, 1H, O*H*), 4.19 (m, 1H, C*H*), 3.92 (dd, 1H, CH*H*), 3.77 (t, 1H, CH*H*), 2.99 (q, 1H, CH*H*), 2.75 (m, 1H, C*H*), 2.64 (q, 1H, CH*H*), 1.28 (d, 3H, CH_3), 1.17 (d, 3H, CH_3). ^{13}C NMR (400 MHz, DMSO-*d6*): 176.81, 157.07, 131.35, 129.79, 114.19, 73.15, 64.50, 40.77, 38.07, 20.13, 16.55

Synthesis of 3-(4-(2-hydroxybutoxy)phenyl)propanoic acid:

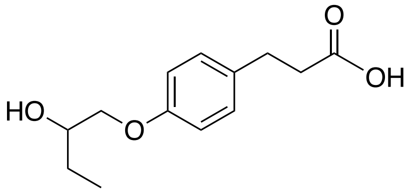

90 g of methyl 3-(4-hydroxyphenyl)propanoate (0.5 mol) was synthesized as previously described[3] and added to a 1 L 3-neck flask with 63.9 g of α-butylene carbonate (1.1 eq.). The general synthesis procedure outlined above was followed and after alkylation the mixture was

refluxed in 500 mL 3M NaOH for 48 h. Acidification and filtration as described above yielded a crude solid that was recrystallized in toluene to yield 103 g of a white powder (86%). ^{1}H NMR (600 MHz, $CDCl_3$): δ 7.12 (d, 2H, H_{Ar}), 6.85 (d, 2H, H_{Ar}), 3.97 (dd, 1H, CH*H*), 3.82 (dd, 1H, CH*H*), 2.90 (t, 2H, CH_2), 2.65 (t, 2H, CH_2), 2.06 (p, 2H, CH_2), 1.03 (t, 3H, CH_3). ^{13}C NMR (400 MHz, DMSO-*d6*): 173.74, 157.04, 132.67, 129.12, 114.32, 71.82, 69.64, 35.53, 29.46, 26.45, 9.77.

Synthesis of 3-(4-(2-hydroxypropoxy)-3-methoxyphenyl)propanoic acid:

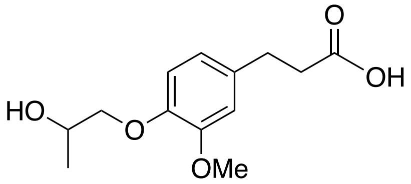

55 g of methyl 3-(4-hydroxy-3-methoxyphenyl)propanoate (0.26 mol) was synthesized as previously described[3] and added to a 1 L 3-neck flask with 29.5 g of propylene carbonate (1.1 eq.). The general synthesis procedure outlined above was followed and after alkylation the mixture was refluxed in 500 mL 3M NaOH for 16 h. Acidification and filtration as described above yielded 56.2 g of a white powder (85%). ^{1}H NMR (600 MHz, DMSO-d_6): δ 12.09 (s, 1H, COO*H*), 6.83 (dd, 2H, H_{Ar}), 6.69 (dd, 1H, H_{Ar}), 4.79 (d, 1H, O*H*), 3.91 (m, 1H, C*H*), 3.78 (q, 1H, CH*H*), 3.73 (s, 3H, OCH_3), 3.67 (q, 1H, CH*H*), 2.74 (t, 2H, CH_2), 2.50 (t, 2H, CH_2), 1.13 (d, 3H, CH_3). ^{13}C NMR (400 MHz, DMSO-*d6*): 174.43, 149.59, 147.16, 134.27, 120.65, 114.33, 113.30, 74.90, 65.18, 56.15, 36.12, 30.59, 20.91.

Synthesis of (E)-3-(4-(2-hydroxyethoxy)phenyl)-2-methylacrylic acid:

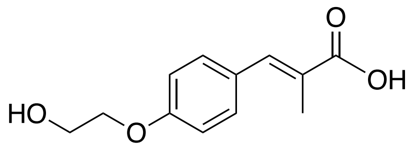

60 g of methyl 3-(4-hydroxyphenyl)-2-methylacrylate (0.34 mol) was added to a 1 L 3-neck flask with 30.2 g of ethylene carbonate (1.1 eq.). The general synthesis procedure outlined above was followed and after alkylation the mixture was refluxed in 500 mL 3M NaOH for 38 h. Acidification and filtration as described above yielded 74.1 g of a tan powder (98%). ^{1}H NMR (600 MHz, DMSO-d_6): δ 12.35 (s, 1H, COO*H*), 7.55 (s, 1H, =C*H*), 7.44 (d, 2H, H_{Ar}), 7.00 (d, 2H, H_{Ar}), 4.88 (s, 1H, O*H*), 4.02 (t, 2H, CH_2), 3.72 (m, 2H, CH_2), 2.03 (s, 3H, CH_3). ^{13}C NMR (400 MHz, DMSO-*d6*): 169.54, 158.84, 137.46, 131.40, 127.87, 126.10, 114.50, 69.58, 59.48, 13.90.

Synthesis of 3-(4-(2-hydroxypropoxy)phenyl)acrylic acid:

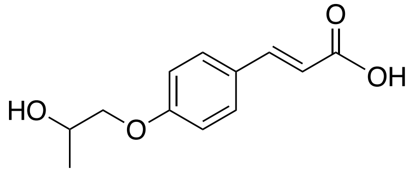

60 g of methyl (3-(4-hydroxyphenyl)acrylate (0.34 mol) were added to a 1 L 3-neck flask with 37.8 g of propylene carbonate (1.1 eq.). The general synthesis procedure outlined above was followed and after alkylation the mixture was refluxed in 500 mL 3M NaOH for 48 h, after which an insoluble mass was removed by filtration. Acidification and filtration as described above yielded a mixture of monomers and dimers as 61.4 g (81%) of a brown solid which was used in the following step without further purification. ^{1}H NMR (600 MHz, DMSO-d_6): δ 12.20

(s, 1H, COO*H*), 7.62 (d, 2H, H_{Ar}), 7.53 (d, 1H, =C*H*), 6.96 (d, 2H, H_{Ar}) 6.36 (d, 1H, =C*H*), 4.89 (d, 1H, O*H*), 3.95 (m, 1H, C*H*), 3.84 (m, 2H, CH_2), 1.15 (d, 3H, CH_3). ^{13}C NMR (400 MHz, DMSO-*d6*): 167.76, 160.40, 143.70, 129.88, 126.73, 116.42, 114.86, 73.29, 64.41, 20.02.

Polyester Synthesis:

Polyester synthesis was conducted according to the procedure outlined in the main body of this text. The polyesters are named according first to their parent glycol (ethylene, iso-propylene, and α-butylene) and then their parent carboxylic acid (phloretic acid, methyl-phloretic acid, hydroferulic acid, coumaric acid, methyl-coumaric acid).

Synthesis of poly-isopropyl phloretate (PiPP):

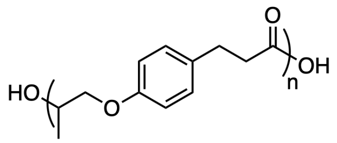

PiPP

3-(4-(2-hydroxypropoxy)phenyl)propanoic acid was synthesized as previously reported and added to a 200 mL 3-neck flask. After following the general procedure, vacuum was applied to the system for 6 h before reaction completion. The resulting brown polymer was collected in quantitative yield on silicon release paper. Spectra agreed with our previously published results.[3]

Synthesis of poly-ortho-isopropyl phloretate (o-PiPP):

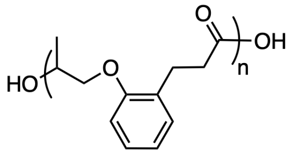

o-PiPP

Following the addition of 60 g of monomer, 1 mol % Sb_2O_3, and 1000 ppm MEHQ to a 200 mL 3-neck flask, the previously described heating ramp from 160-220°C at 10°C/h. was followed. After completion of the heating ramp, vacuum was maintained for 5 h until reaction completion and the resultant light grey polymer was collected in quantitative yield on silicon

release paper. ^{1}H NMR (600 MHz, $CDCl_3$): δ 7.11 (m, 2H, H_{Ar}), 6.81 (t, 1H, H_{Ar}), 6.74 (d, 1H, H_{Ar}), 5.24 (m, 1H, C*H*), 3.94 (m, 1H, CH*H*), 3.85 (m, 1H, CH*H*), 2.89 (m, 2H, CH_2), 2.59 (m, 2H, CH_2), 1.30 (d, 3H, CH_3). ^{13}C NMR (400 MHz, $CDCl_3$): δ 172.72, 156.49, 130.29, 129.06, 120.93, 111.28, 69.96, 68.69, 34.35, 26.32, 17.02.

Synthesis of (*R*)-poly-isopropyl phloretate ((*R*)-PiPP):

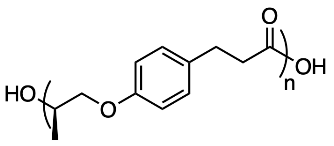

(R)-PiPP

Following the addition of 180 g of monomer, 1 mol % Sb_2O_3, and 1000 ppm MEHQ to a 500 mL 3-neck flask, the previously described heating ramp from 160-220°C at 10°C/h. was followed. After completion of the heating ramp, vacuum was maintained for 9 h until reaction completion and the resultant grey polymer was collected in quantitative yield on silicon release paper. ^{1}H NMR (600 MHz, $CDCl_3$): δ 7.10 (d, 2H, H_{Ar}), 6.79 (d, 2H, H_{Ar}), 5.23 (m, 1H, C*H*), 3.92 (dd, 2H, CH*H*), 2.87 (t, 2H, CH_2), 2.58 (t, 2H, CH_2), 1.31 (d, 3H, CH_3). ^{13}C NMR (400 MHz, $CDCl_3$): δ 172.53, 157.29, 133.17, 129.44, 114.83, 70.27, 69.02, 36.45, 30.27, 16.84.

Synthesis of poly-ethylene methyl phloretate (PEMP):

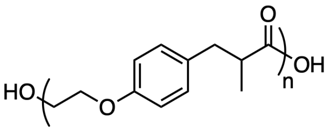

PEMP

Following the addition of 50 g of monomer, 1 mol % Sb_2O_3, and 1000 ppm MEHQ to a 100 mL 3-neck flask, the previously described heating ramp from 160-220°C at 10°C/h. was followed. After completion of the heating ramp, vacuum was maintained for 10 h until reaction completion and the resultant dark brown polymer was collected in quantitative yield on silicon release paper. ^{1}H NMR (600 MHz, $CDCl_3$): δ 7.07 (d, 2H, H_{Ar}), 6.76 (d, 2H, H_{Ar}), 4.35 (m, 2H, CH_2), 4.04 (m, 2H, CH_2), 2.95 (q, 1H, CH*H*), 3.73 (m, 1H, C*H*), 2.63 (q, 1H, CH*H*), 1.14 (d, 3H, CH_3). ^{13}C NMR (400 MHz, $CDCl_3$): δ 176.11, 157.25, 131.97, 130.18, 114.67, 66.06, 62.85, 41.69, 38.90, 16.81.

Synthesis of poly-isopropyl methyl phloretate (PiPMP):

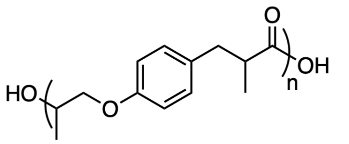

PiPMP

Following the addition of 60 g of monomer, 1 mol % Sb_2O_3, and 1000 ppm MEHQ to a 200 mL 3-neck flask, the previously described heating ramp from 160-220°C at 10°C/h. was followed. After completion of the heating ramp, vacuum was maintained for 15 h until reaction completion and the resultant brown polymer was collected in quantitative yield on silicon release paper. ^{1}H NMR (600 MHz, $CDCl_3$): δ 7.10 (m, 2H, H_{Ar}), 6.79 (m, 2H, H_{Ar}), 5.22 (m, 1H, C*H*), 3.91 (m, 2H, CH_2), 2.95 (m, 1H, CH*H*), 2.70 (p, 1H, C*H*), 2.62 (m, 1H, CH*H*), 1.29 (m, 3H, CH_3), 1.14 (m, 3H, CH_3). ^{13}C NMR (400 MHz, $CDCl_3$): δ 175.68, 157.36, 132.01, 130.14, 114.65, 70.19, 68.77, 41.92, 41.74, 38.99, 38.86, 16.84.

Synthesis of poly-α-butylene phloretate (PαBP):

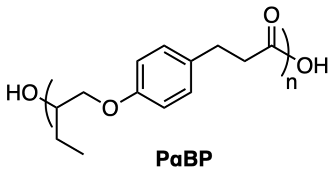

PaBP

Following the addition of 60 g of monomer, 1 mol % Sb_2O_3, and 1000 ppm MEHQ to a 200 mL 3-neck flask, the previously described heating ramp from 160-220°C at 10°C/h. was followed. After completion of the heating ramp, vacuum was maintained for 8.3 h until reaction completion and the resultant light grey polymer was collected in quantitative yield on silicon release paper. ^{1}H NMR (600 MHz, $CDCl_3$): δ 7.10 (d, 2H, H_{Ar}), 6.78 (d, 2H, H_{Ar}), 5.11 (m, 1H, CH*H*), 3.93 (m, 2H, CH_2), 2.88 (m, 2H, CH_2), 2.60 (t, 2H, CH_2), 1.70 (m, 2H, CH_2), 0.90 (t, 3H, CH_3). ^{13}C NMR (400 MHz, $CDCl_3$): δ 172.75, 157.35, 133.13, 129.40, 114.83, 73.53, 68.69, 36.39, 30.32, 24.04, 9.67.

Synthesis of poly-isopropyl dihydroferulate (PiPHF):

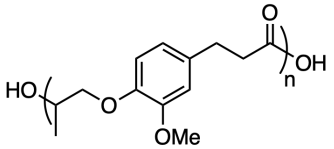

PiPHF

Following the addition of 50 g of monomer, 1 mol % Sb_2O_3, and 1000 ppm MEHQ to a 100 mL 3-neck flask, the previously described heating ramp from 160-220°C at 10°C/h. was followed. After completion of the heating ramp, vacuum was maintained for 8.5 h until reaction completion and the resultant light tan polymer was collected in quantitative yield on silicon

release paper. ^{1}H NMR (600 MHz, $CDCl_3$): δ 6.79(d, 1H, H_{Ar}), 6.71 (m, 2H, H_{Ar}), 5.25 (m, 1H, CH), 4.00 (m, 2H, CH_2), 3.80 (s, 3H, OCH_3), 2.88 (t, 2H, CH_2), 2.60 (t, 2H, CH_2), 1.31 (d, 3H, CH_3). ^{13}C NMR (400 MHz, $CDCl_3$): δ 172.54, 150.09, 146.85, 134.68, 120.51, 115.30, 112.92, 72.05, 69.24, 56.17, 36.43, 30.76, 16.88.

Synthesis of poly-ethylene methyl cinnamate (PEMC):

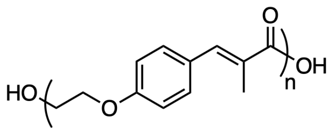

PEMC

Following the addition of 50 g of monomer, 1 mol % Sb_2O_3, and 1000 ppm MEHQ to a 100 mL 3-neck flask, the previously described heating ramp from 160-220°C at 10°C/h. was followed. After completion of the heating ramp, vacuum was maintained for 10 h until reaction completion and the resultant brown, brittle polymer was collected in quantitative yield on silicon release paper. ^{1}H NMR (600 MHz, $CDCl_3$): δ 7.65 (s, 1H, =CH), 7.37 (d, 2H, H_{Ar}), 6.96 (d, 2H, H_{Ar}), 4.56 (approximately t, 2H, CH_2), 4.29 (approximately t, 2H, CH_2), 2.12 (s, 3H, CH_3). ^{13}C NMR (400 MHz, $CDCl_3$): δ 168.88, 158.99, 139.27, 131.68, 128.94, 126.17, 114.79, 66.29, 63.27, 14.24.

Synthesis of poly-isopropyl cinnamate (PiPC):

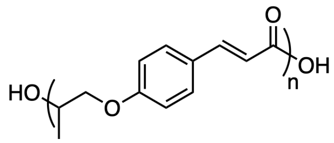

PiPC

Following the addition of 60 g of monomer, 1 mol % Sb_2O_3, and 1000 ppm MEHQ to a 200 mL 3-neck flask, the previously described heating ramp from 160-220°C at 10°C/h. was followed. After completion of the heating ramp, vacuum was maintained for 12 h until reaction completion and the resultant dark brown, brittle polymer was collected in quantitative yield on silicon release paper. Spectral data was extremely poor quality due to the high gel content and low solubility of the crosslinked polyester. Several solvent systems were attempted ($CDCl_3$, TFA-*d*, DMSO, and cosolvents) with equally poor spectral resolution.

Thermal Characterization of Polymers

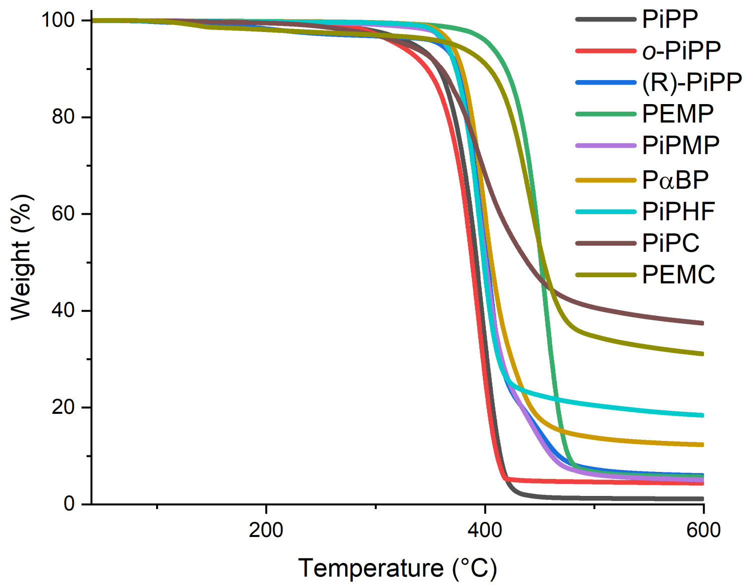

Figure S1. TGA overlay of polyesters synthesized in this study.

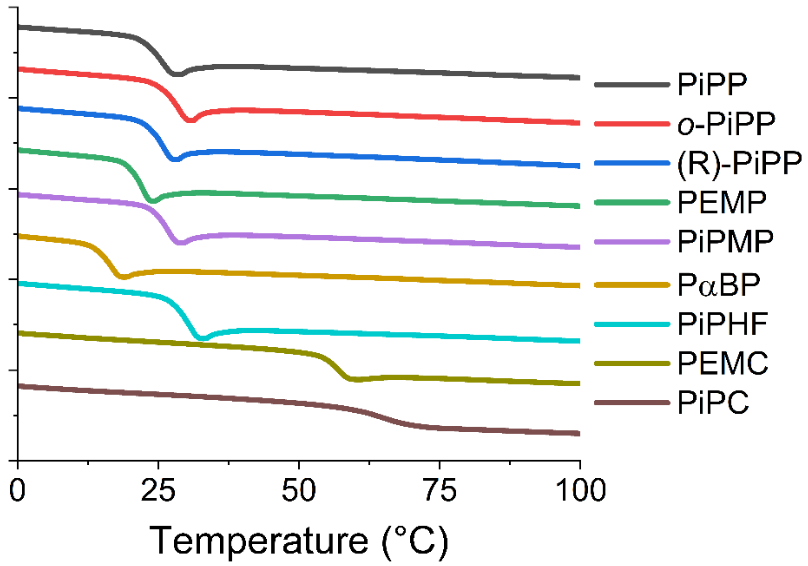

Figure S2. DSC overlay of polyesters synthesized in this study.

Injection Molding of Polymers

Table S1. Summary of extrusion and injection molding conditions

Polymer	Extrusion Temp (°C)	Mold Temp (°C)	Pressure (bar)
PαBP	100	RT then Ice Bath	350
(R)-PiPP	120	23 (RT)	350
PiPMP	120	25 (RT)	350
PiPP	120	25 (RT)	350
PiPHF	140	24 (RT)	350
o-PiPP	140	28	350
PEMC	160	58	350
PiPC	170	90	350
PEMP	120	24 (RT)	350

Rheological Characterization of Polyesters:

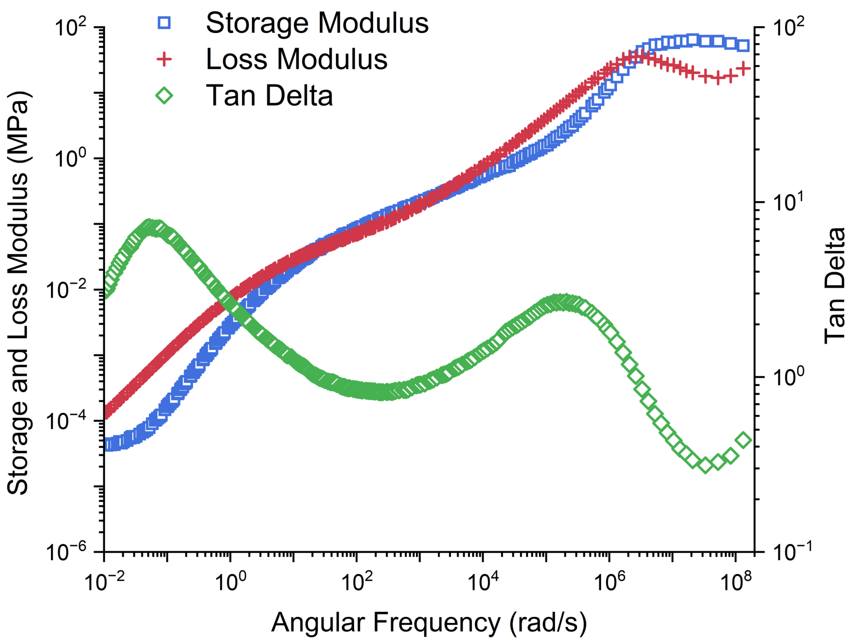

Figure S3. TTS master curve of *o*-PiPP.

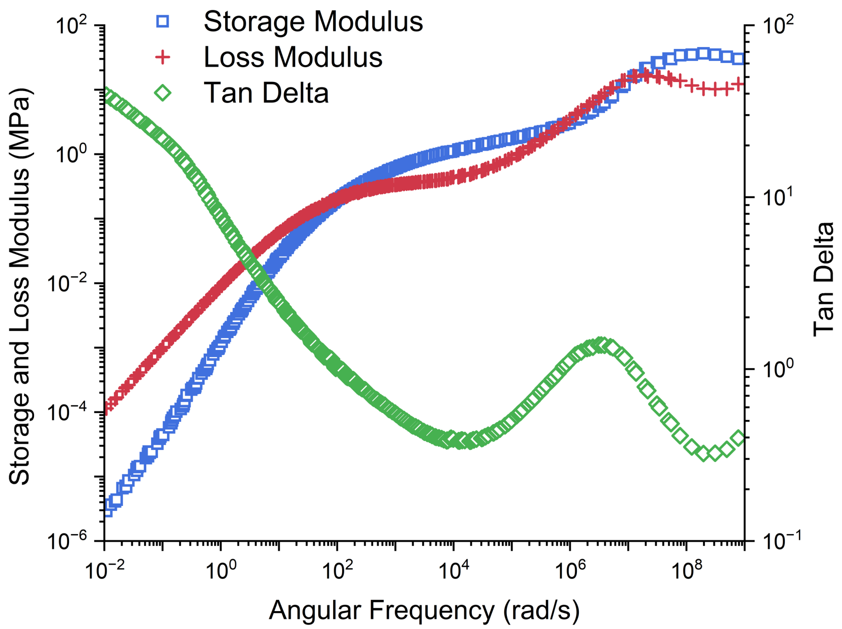

Figure S4. TTS master curve of (*R*)-PiPP.

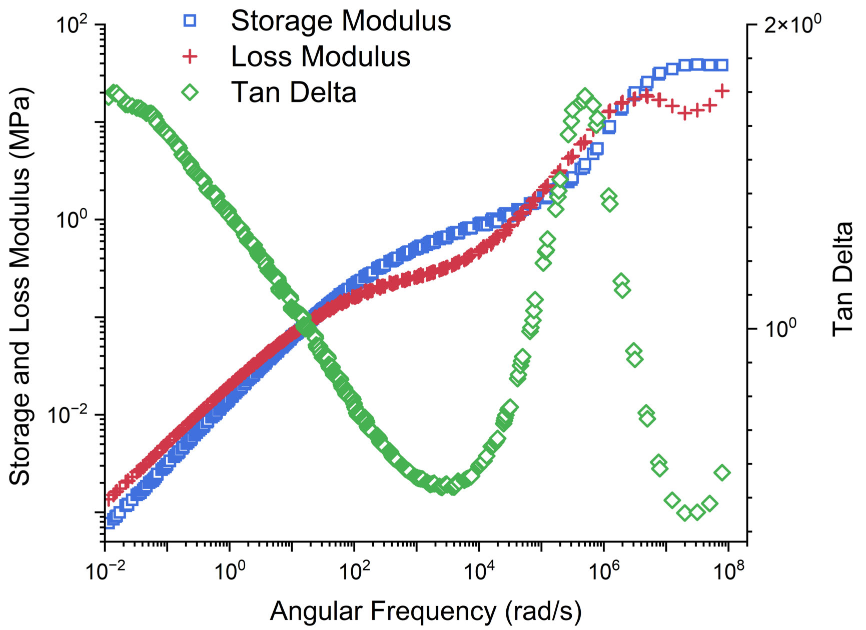

Figure S5. TTS master curve of PEMP.

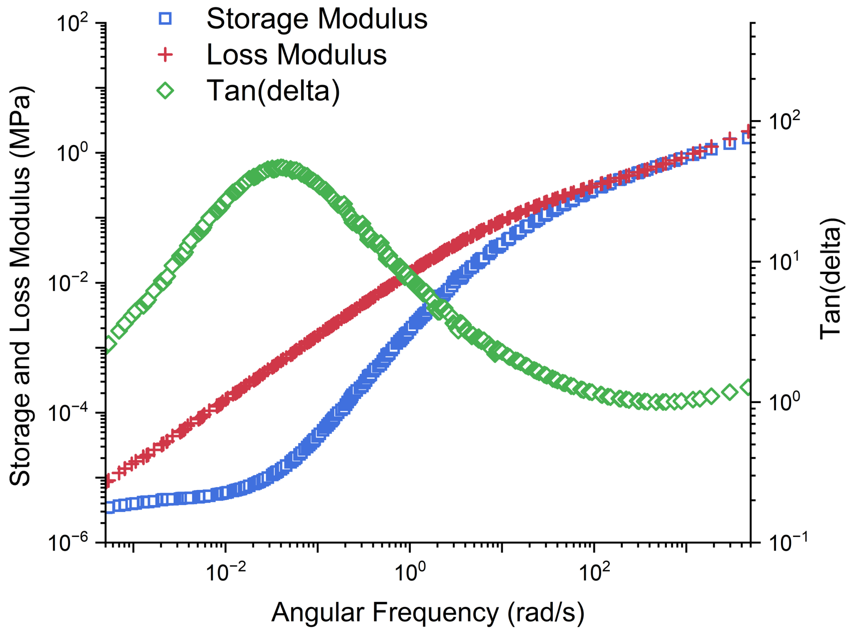

Figure S6. TTS master curve of PiPMP.

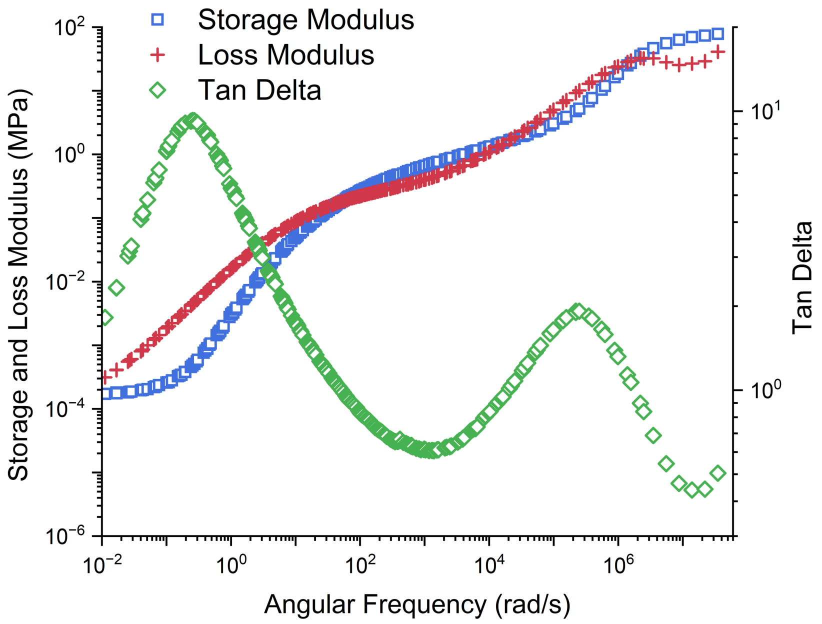

Figure S7. TTS master curve of PαBP.

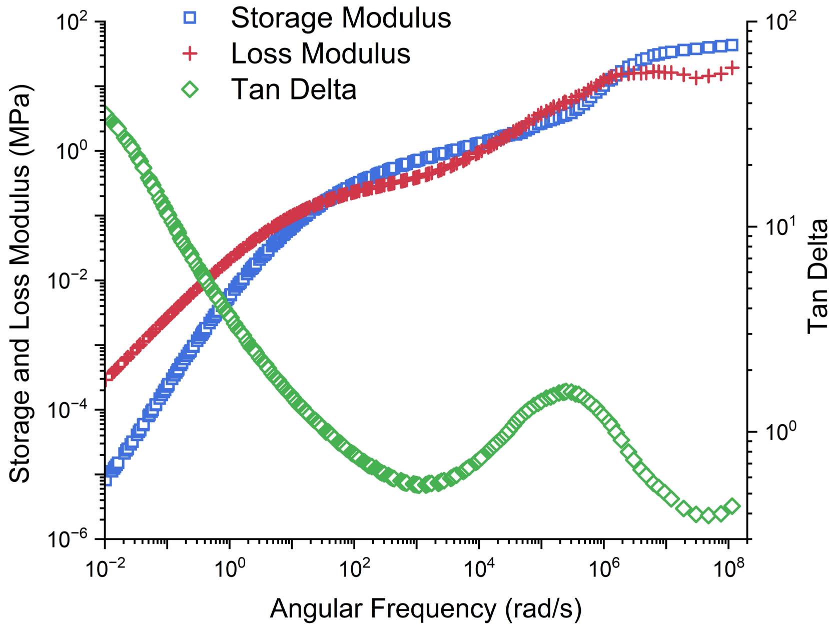

Figure S8. TTS master curve of PiPHF.

^{1}H NMR Spectra of Synthesized Precursors, Monomers, and Polymers

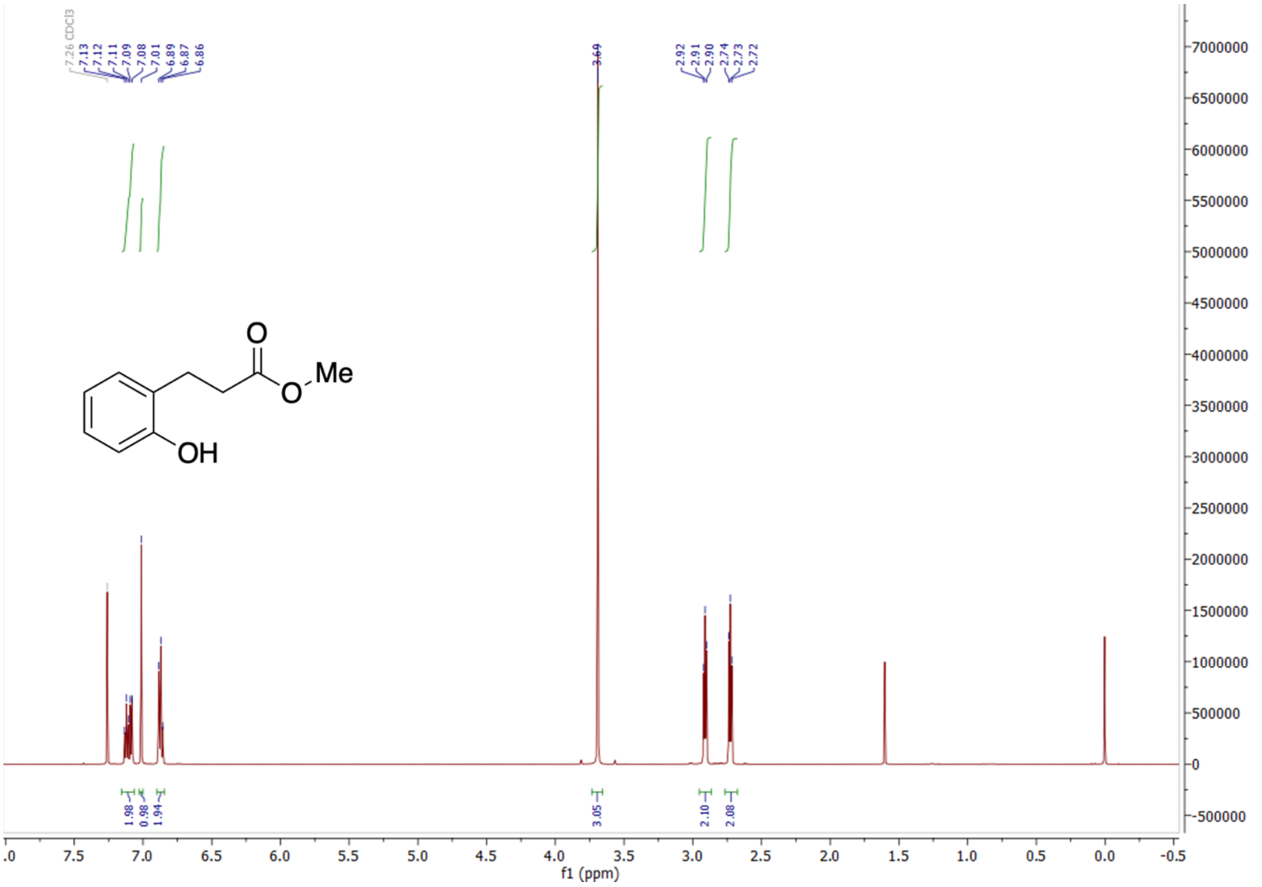

Figure S9. ^{1}H NMR spectrum of methyl 3-(2-hydroxyphenyl)propanoate in $CDCl_3$.

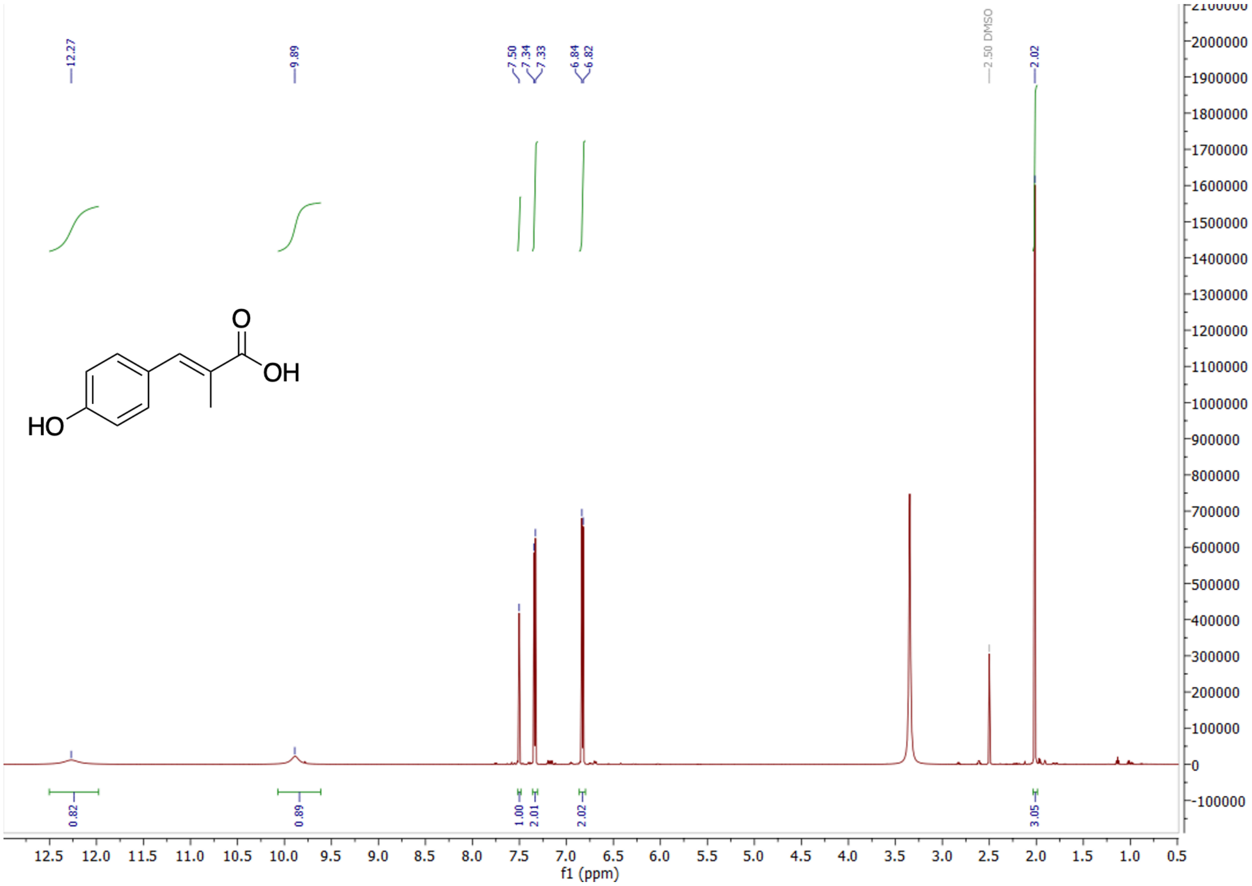

Figure S10. ^{1}H NMR spectrum of 3-(4-hydroxyphenyl)-2-methylacrylic acid in DMSO-*d6*.

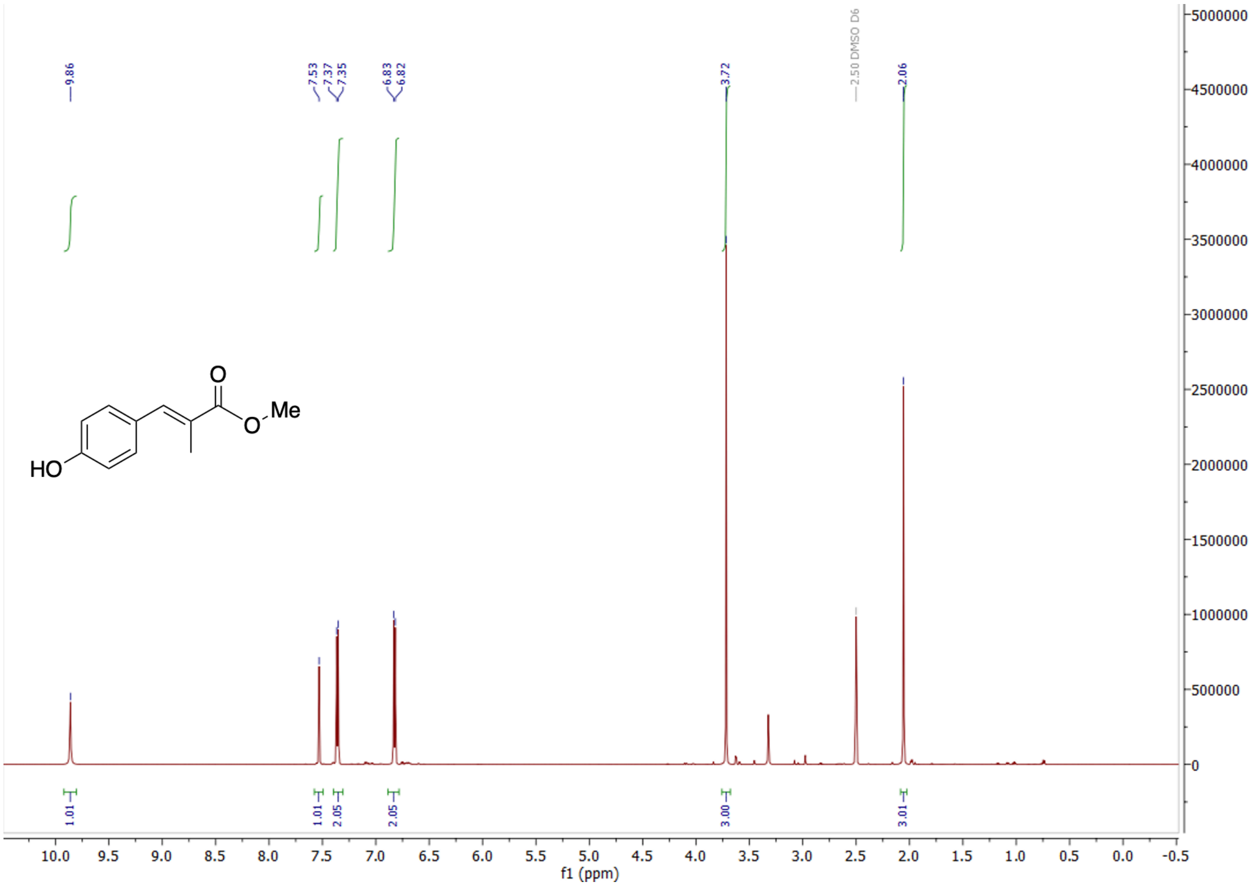

Figure S11. ^{1}H NMR spectrum of methyl 3-(4-hydroxyphenyl)-2-methylacrylate in DMSO-*d6*.

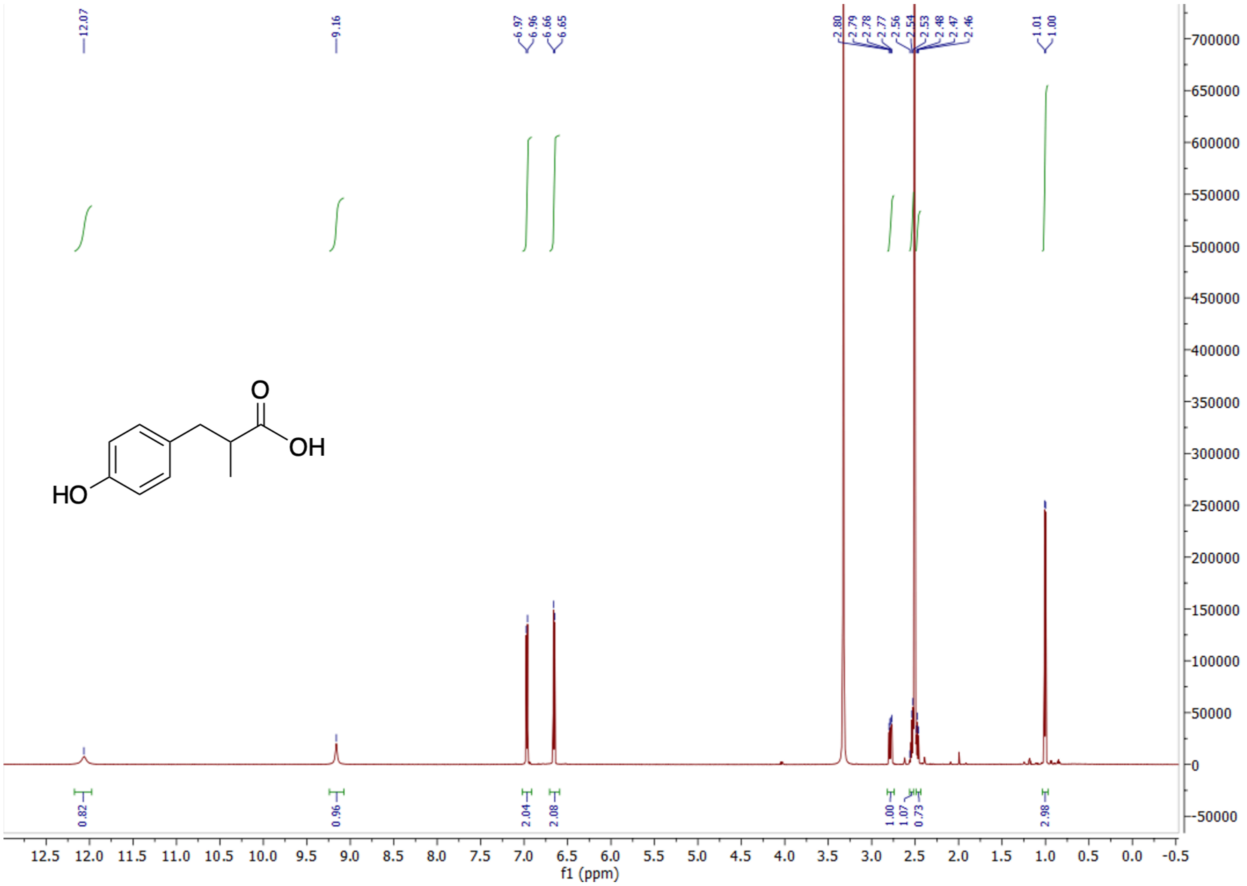

Figure S12. ^{1}H NMR spectrum of 3-(4-hydroxyphenyl)-2-methylpropanoic acid in DMSO-*d6*.

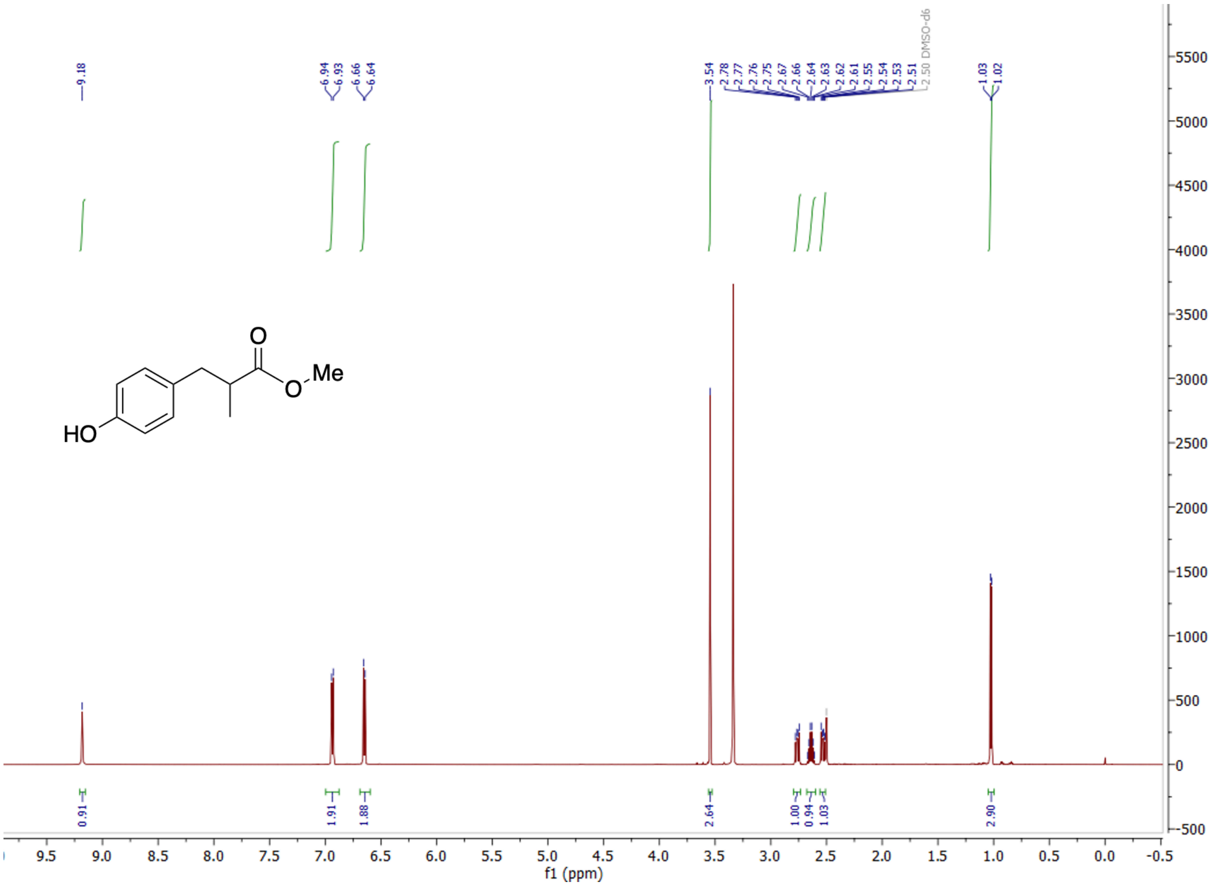

Figure S13. ^{1}H NMR spectrum of methyl 3-(4-hydroxyphenyl)-2-methylpropanoate in DMSO-*d6*.

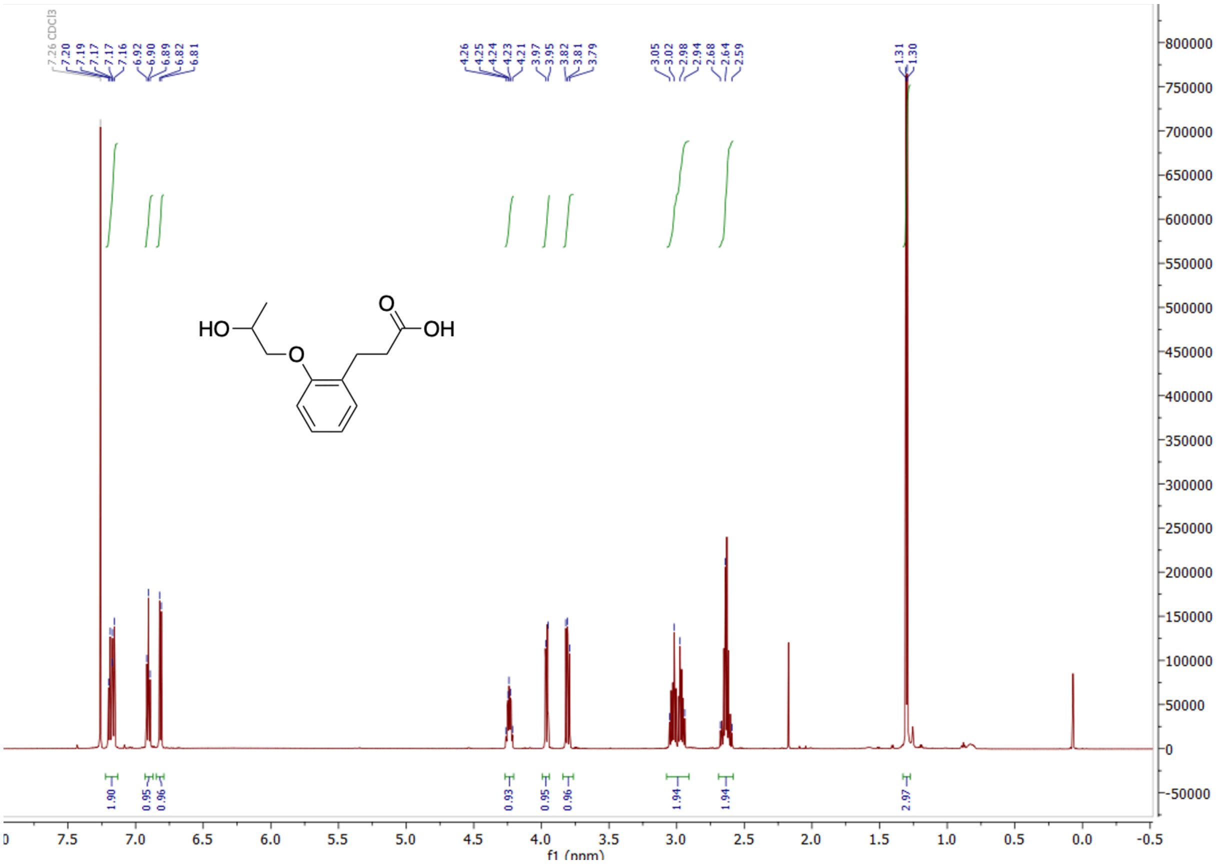

Figure S14. ^{1}H NMR spectrum of 3-(2-(2-hydroxypropoxy)phenyl)propanoic acid in $CDCl_3$.

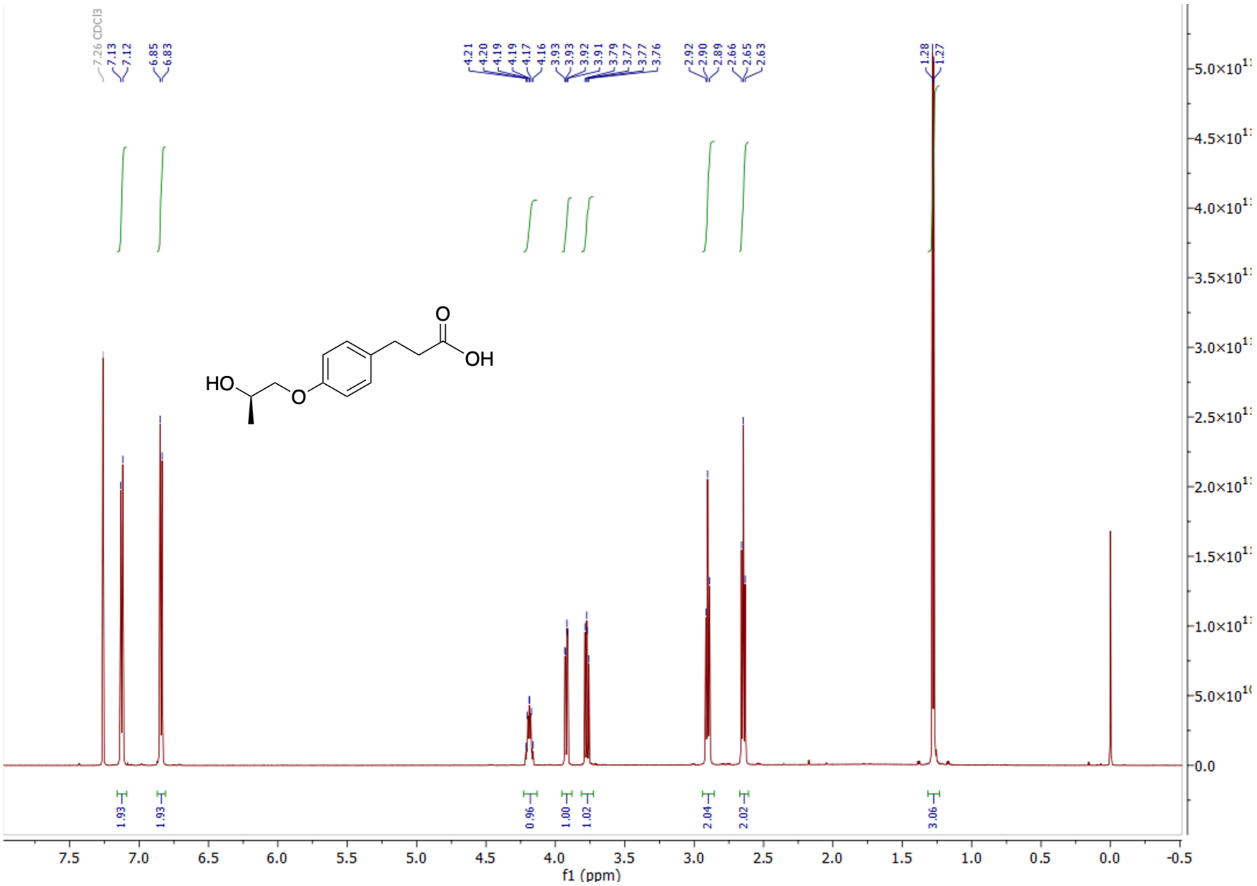

Figure S15. ^{1}H NMR spectrum of (*R*)-3-(4-(2-hydroxypropoxy)phenyl)propanoic acid in $CDCl_3$.

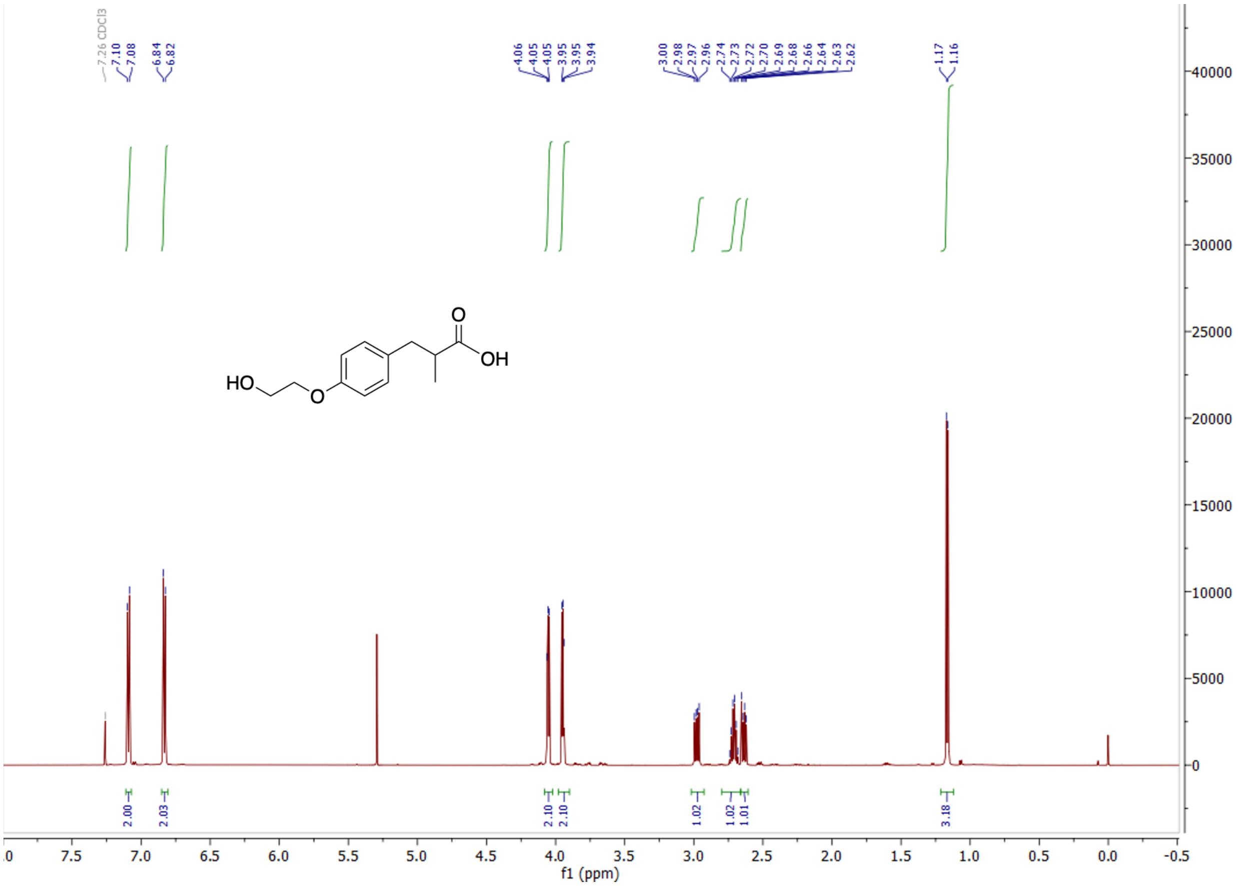

Figure S16. ^{1}H NMR spectrum of 3-(4-(2-hydroxyethoxy)phenyl)-2-methylpropanoic acid in $CDCl_3$.

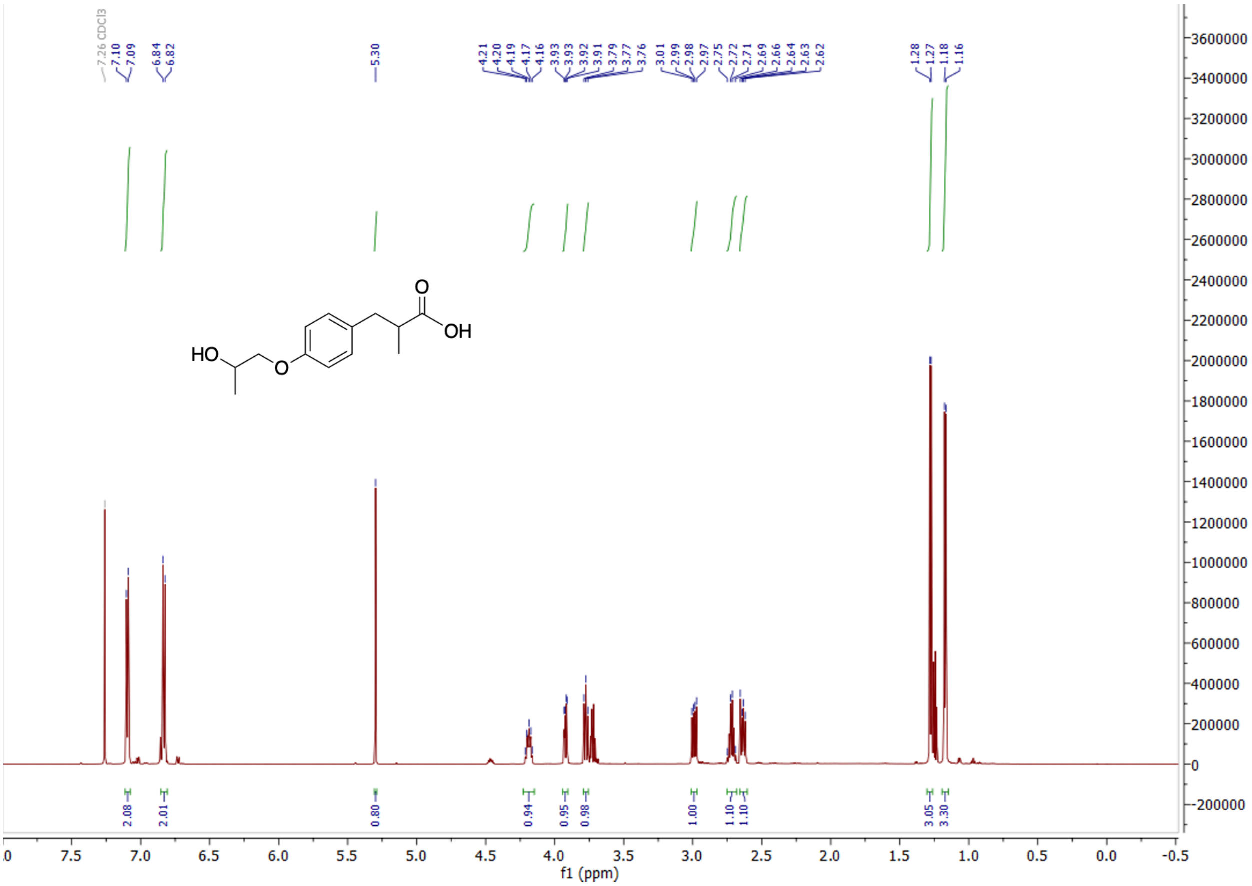

Figure S17. ^{1}H NMR spectrum of 3-(4-(2-hydroxypropoxy)phenyl)-2-methylpropanoic acid in $CDCl_3$.

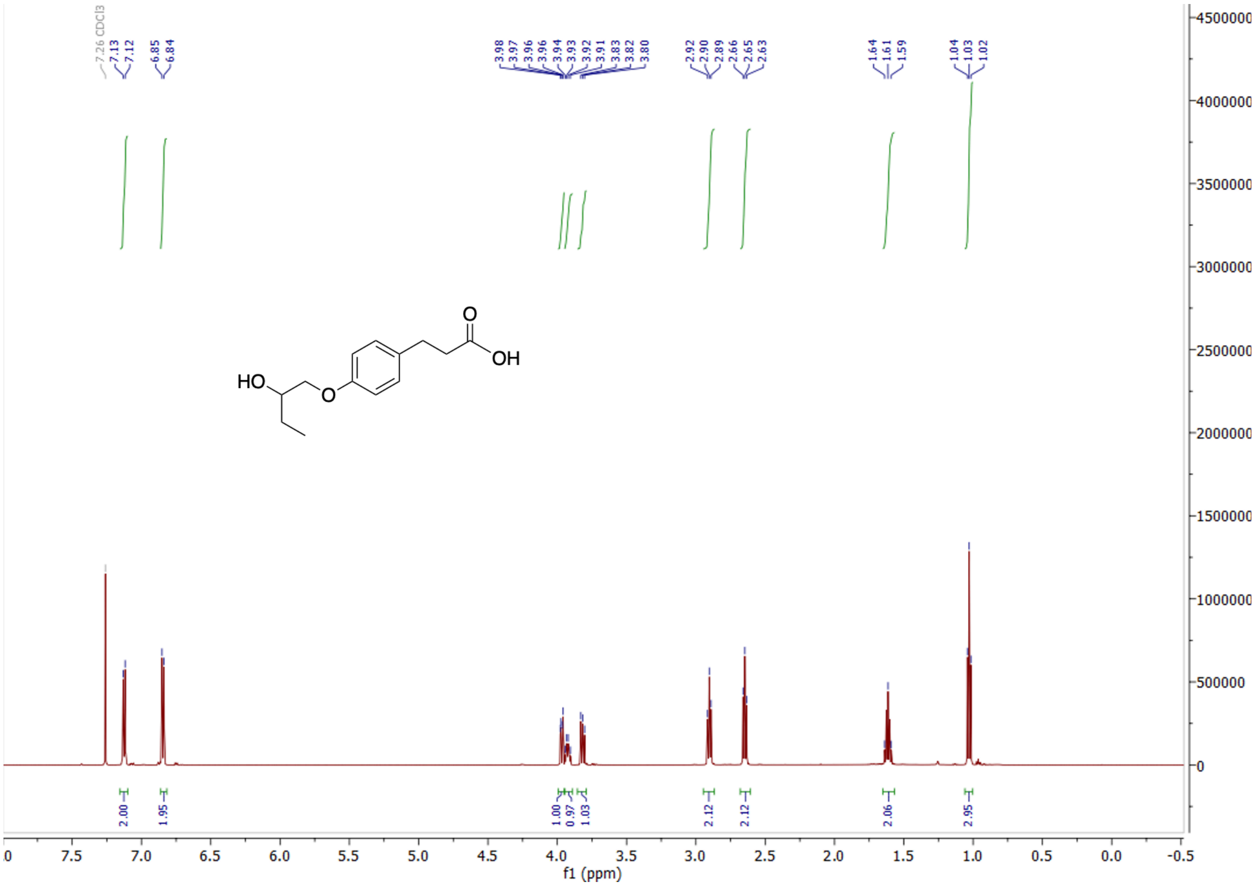

Figure S18. ^{1}H NMR spectrum of 3-(4-(2-hydroxybutoxy)phenyl)propanoic acid in $CDCl_3$.

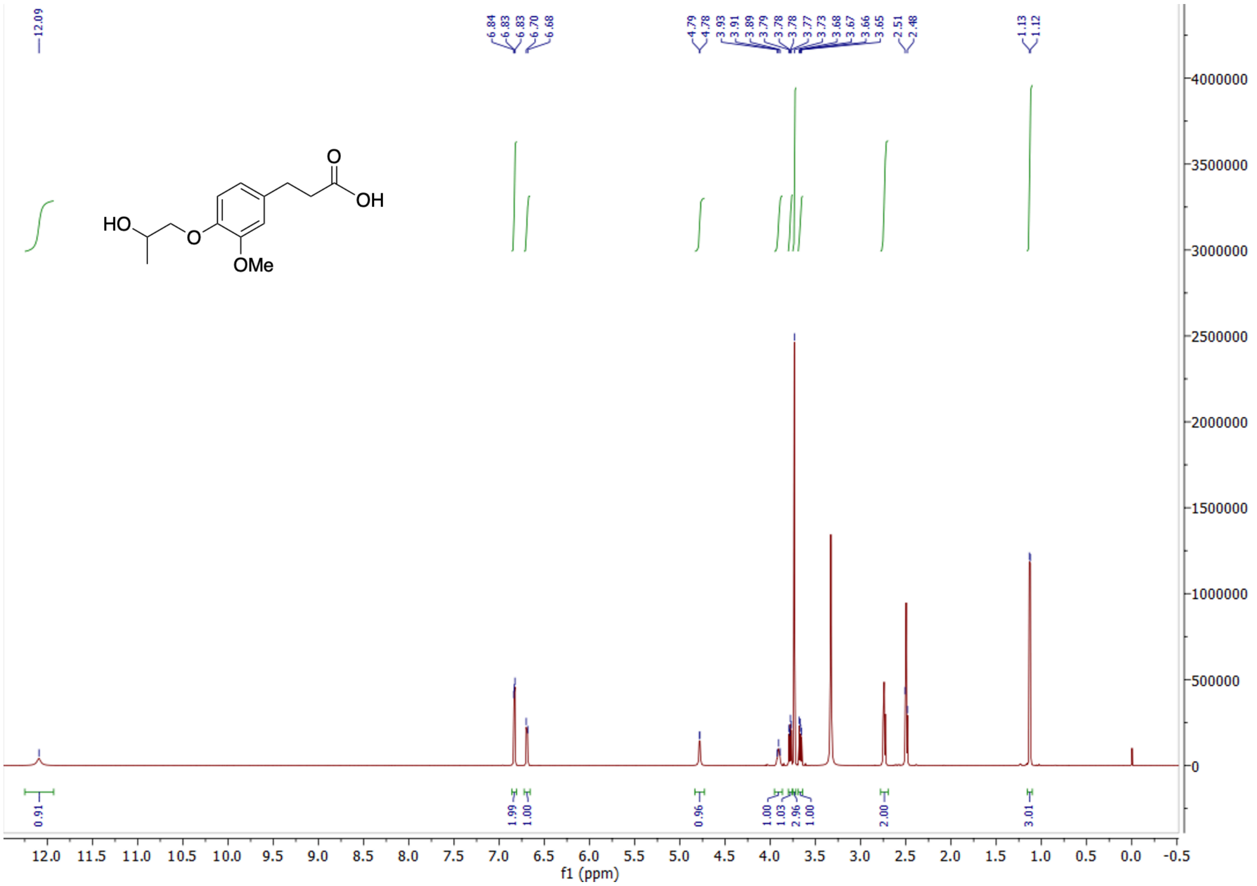

Figure S19. ^{1}H NMR spectrum of 3-(4-(2-hydroxypropoxy)-3-methoxyphenyl)propanoic acid in DMSO-*d6*.

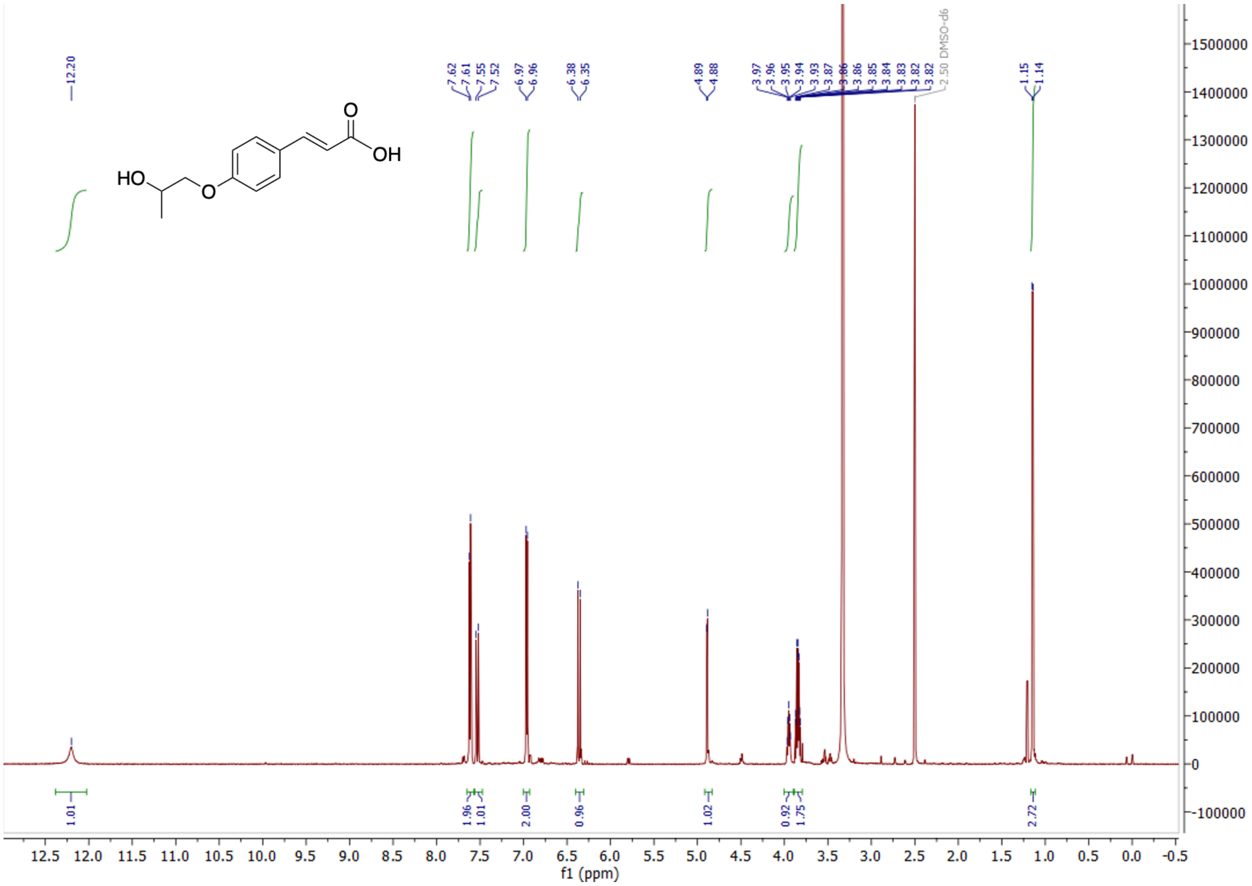

Figure S20. ^{1}H NMR spectrum of 3-(4-(2-hydroxypropoxy)phenyl)acrylic acid in DMSO-*d6*.

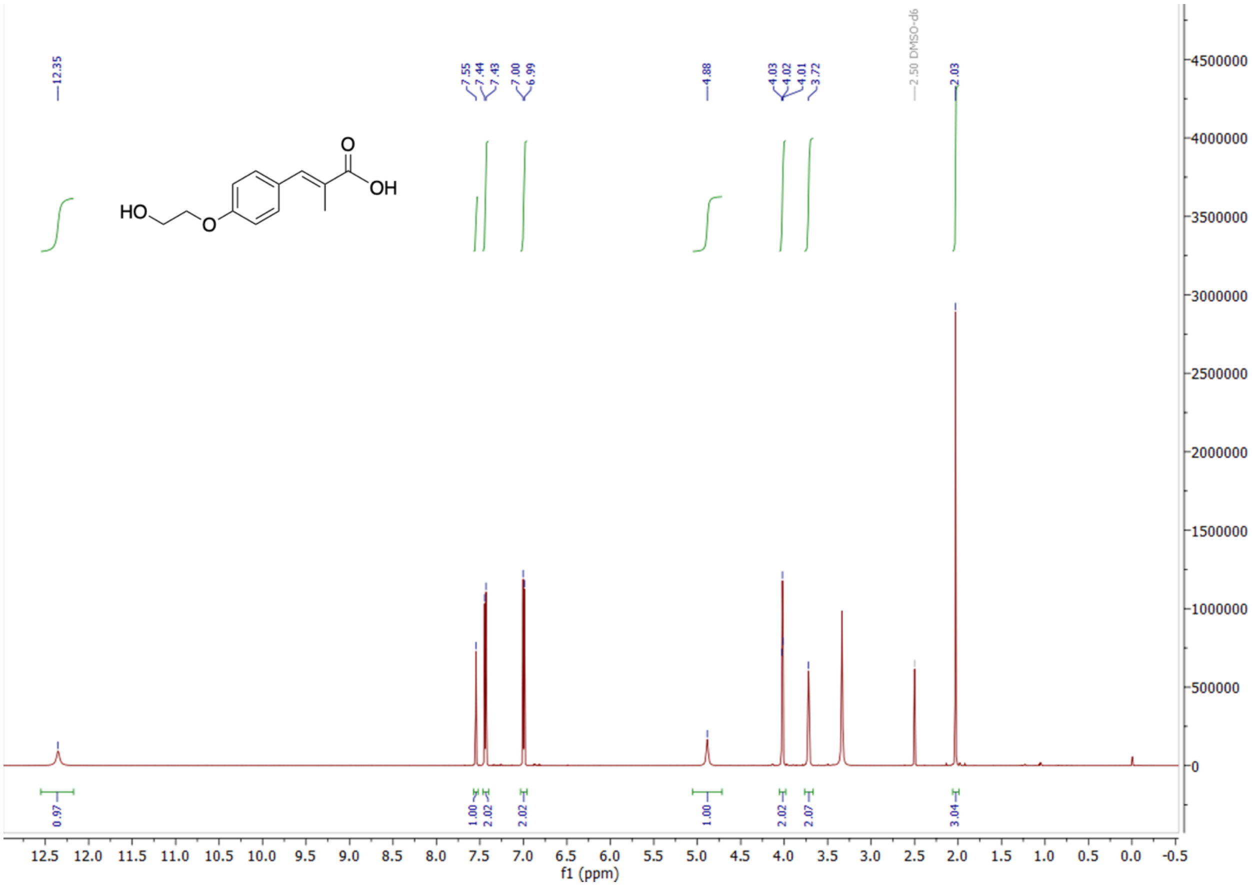

Figure S21. ^{1}H NMR spectrum of (E)-3-(4-(2-hydroxyethoxy)phenyl)-2-methylacrylic acid in DMSO-*d6*.

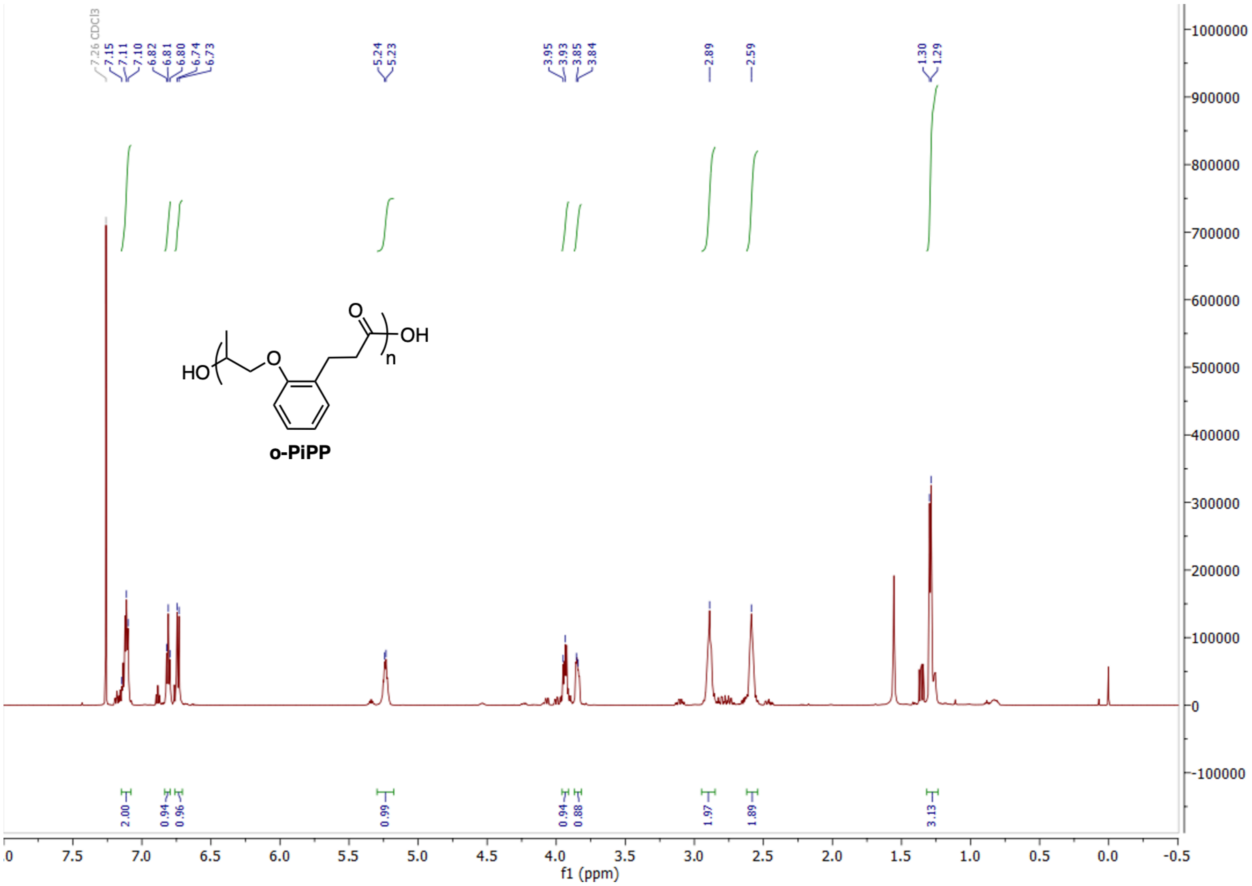

Figure S22. ^{1}H NMR spectrum of poly-*ortho*-isopropyl phloretate (*o*-PiPP) in $CDCl_3$.

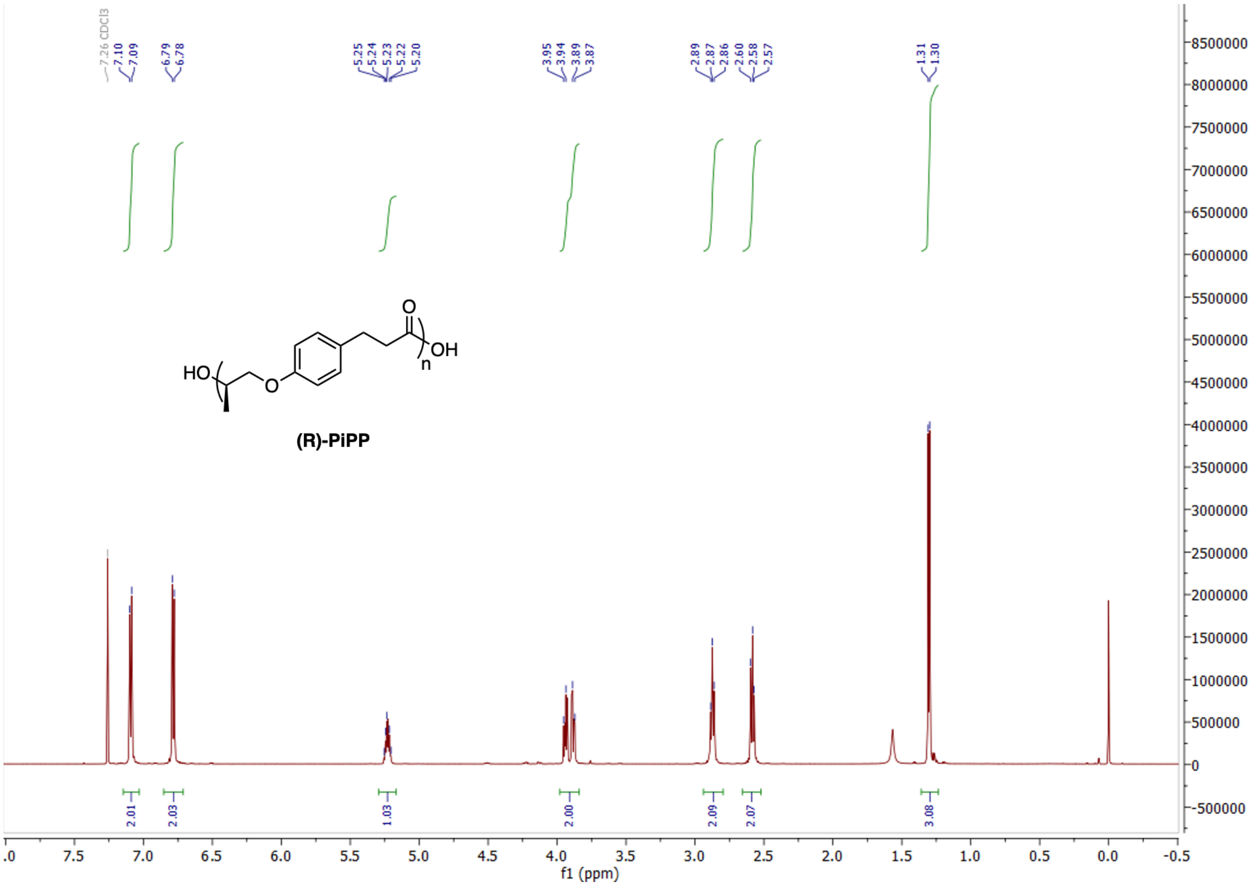

Figure S23. ^{1}H NMR spectrum of (*R*)-poly-isopropyl phloretate ((*R*)-PiPP) in $CDCl_3$.

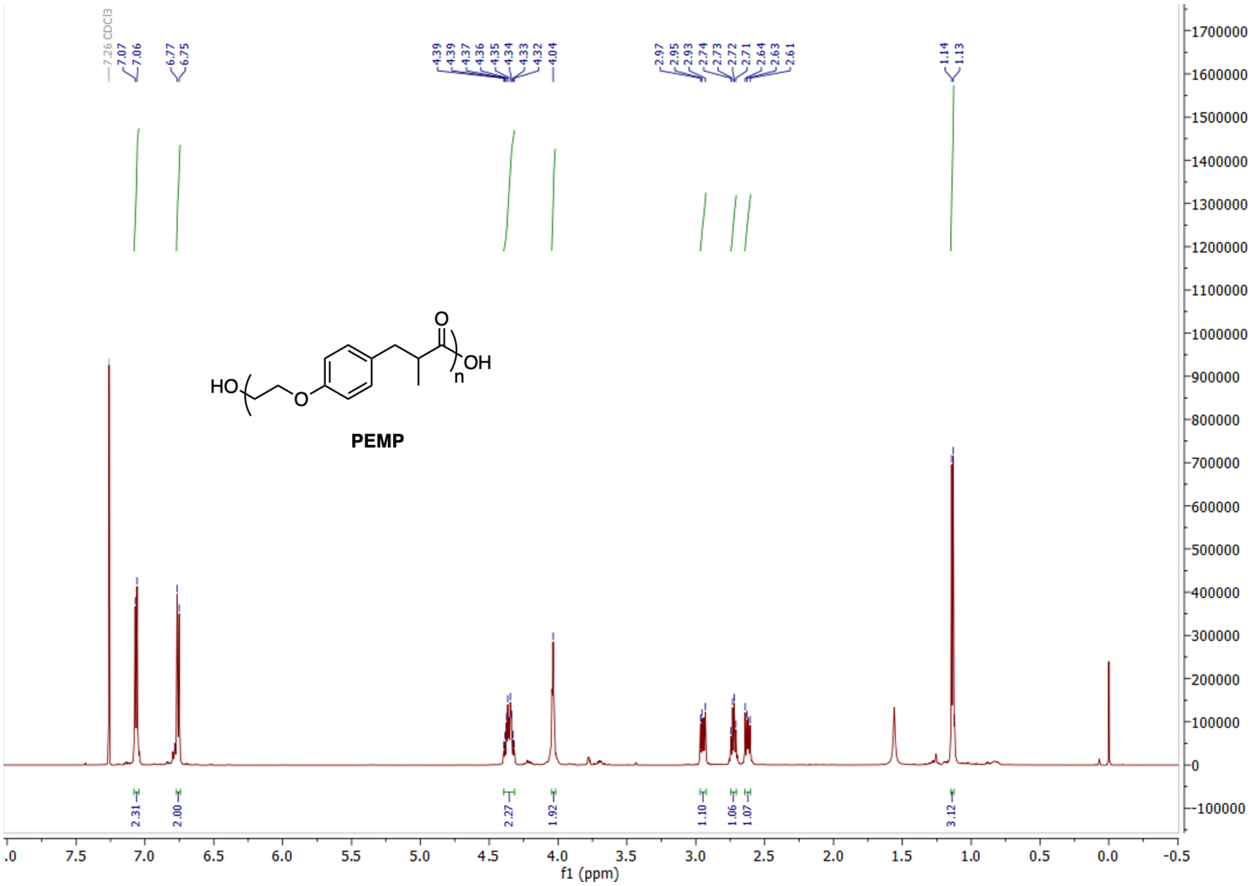

Figure S24. ^{1}H NMR spectrum of poly-ethylene methyl phloretate (PEMP) in $CDCl_3$.

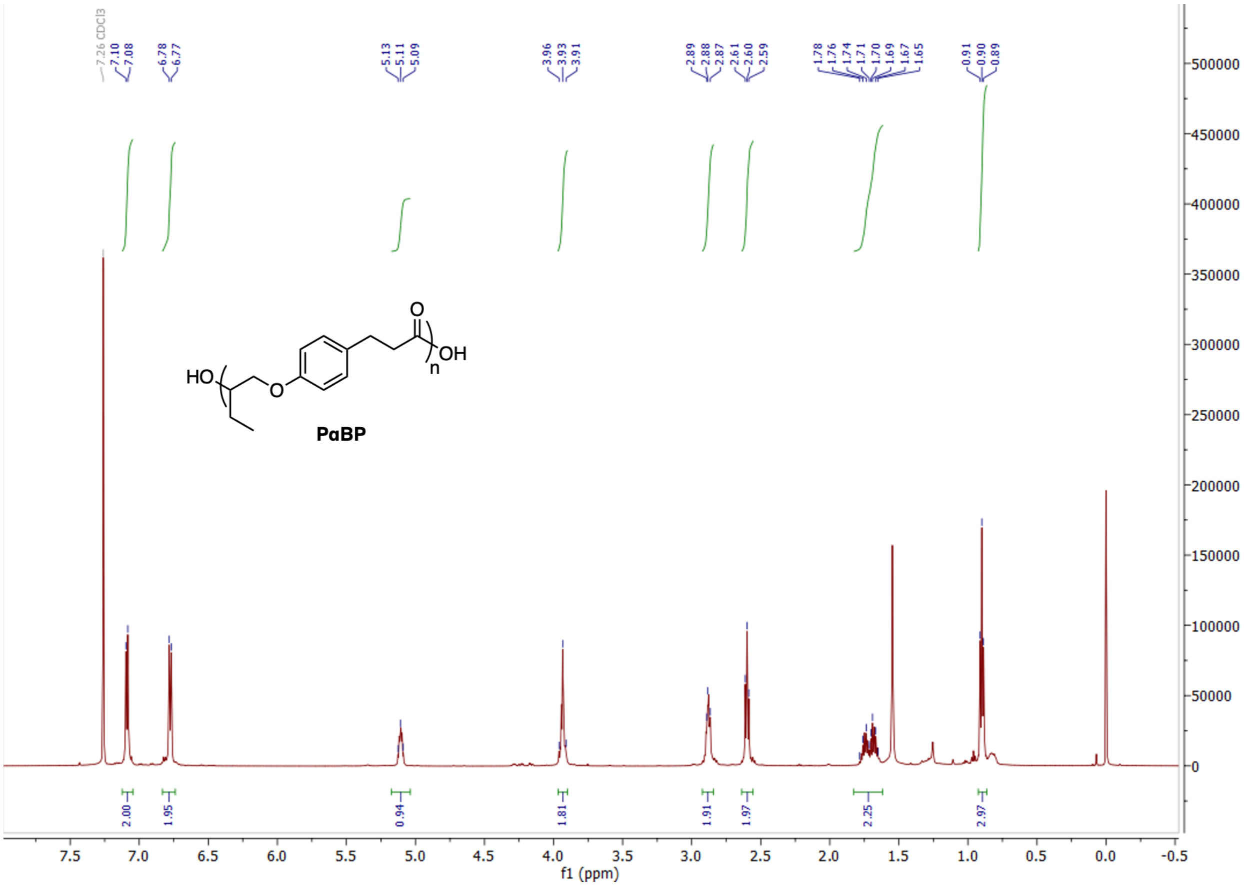

Figure S25. ^{1}H NMR spectrum of poly-α-butylene phloretate (PαBP) in $CDCl_3$.

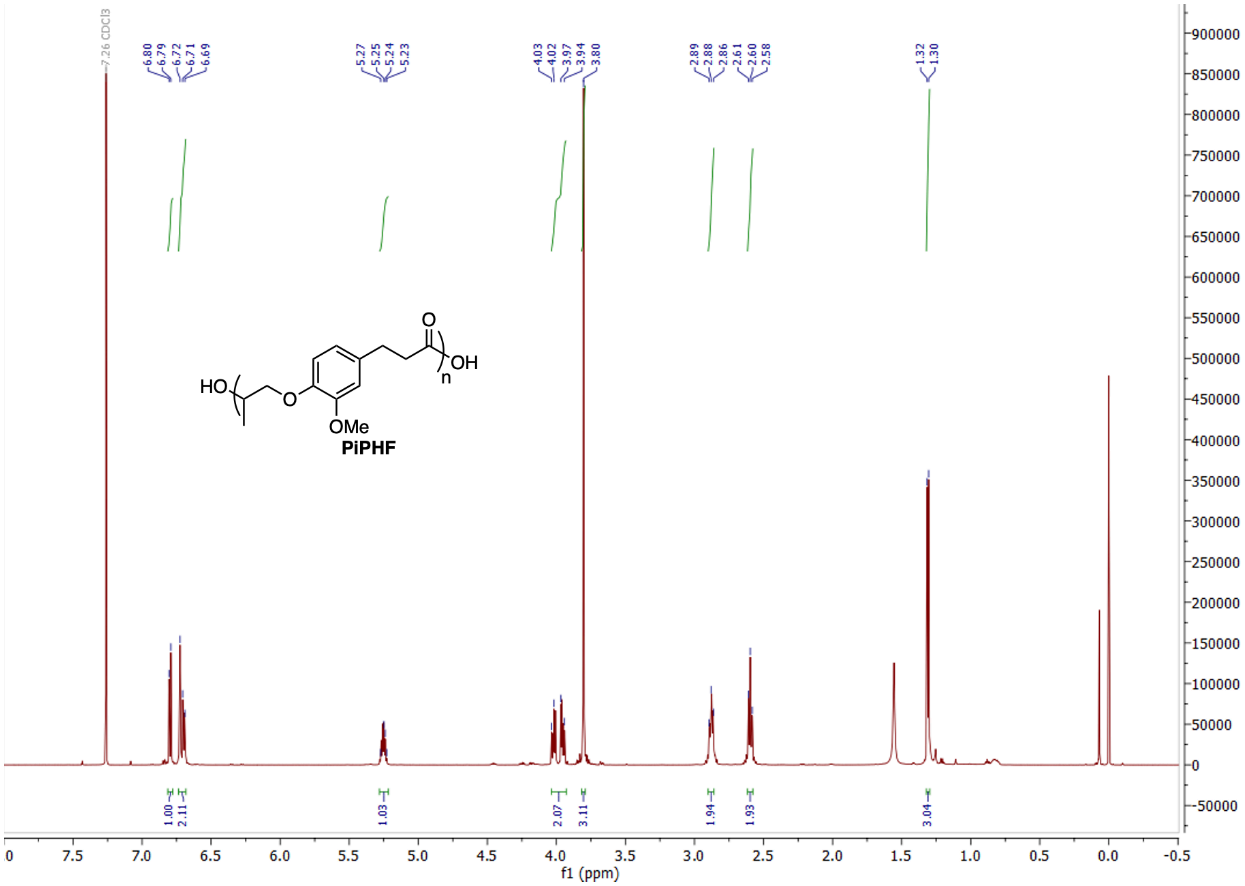

Figure S25. ^{1}H NMR spectrum of poly-isopropyl dihydroferulate (PiPHF) in $CDCl_3$.

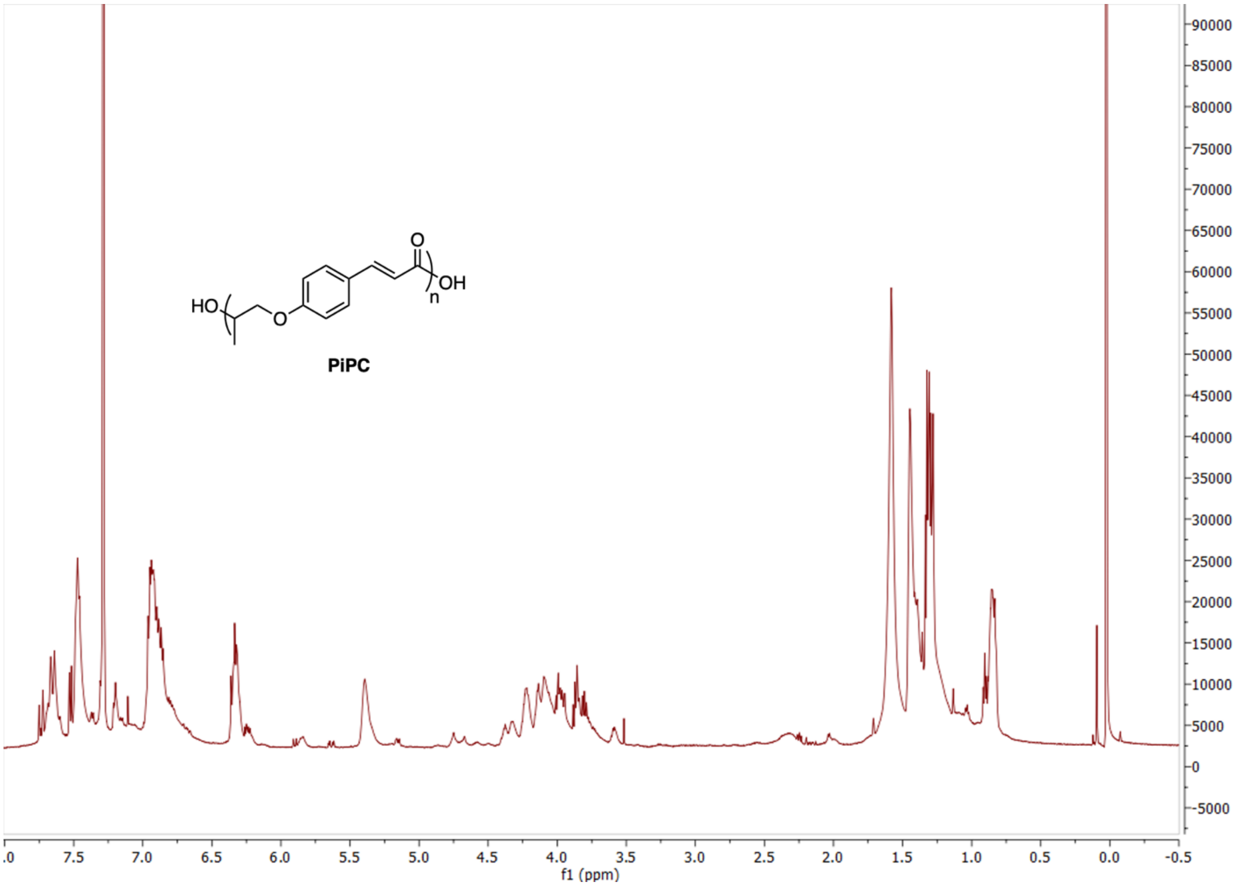

Figure S26. ^{1}H NMR spectrum of poly-isopropyl cinnamate (PiPC) in $CDCl_3$.

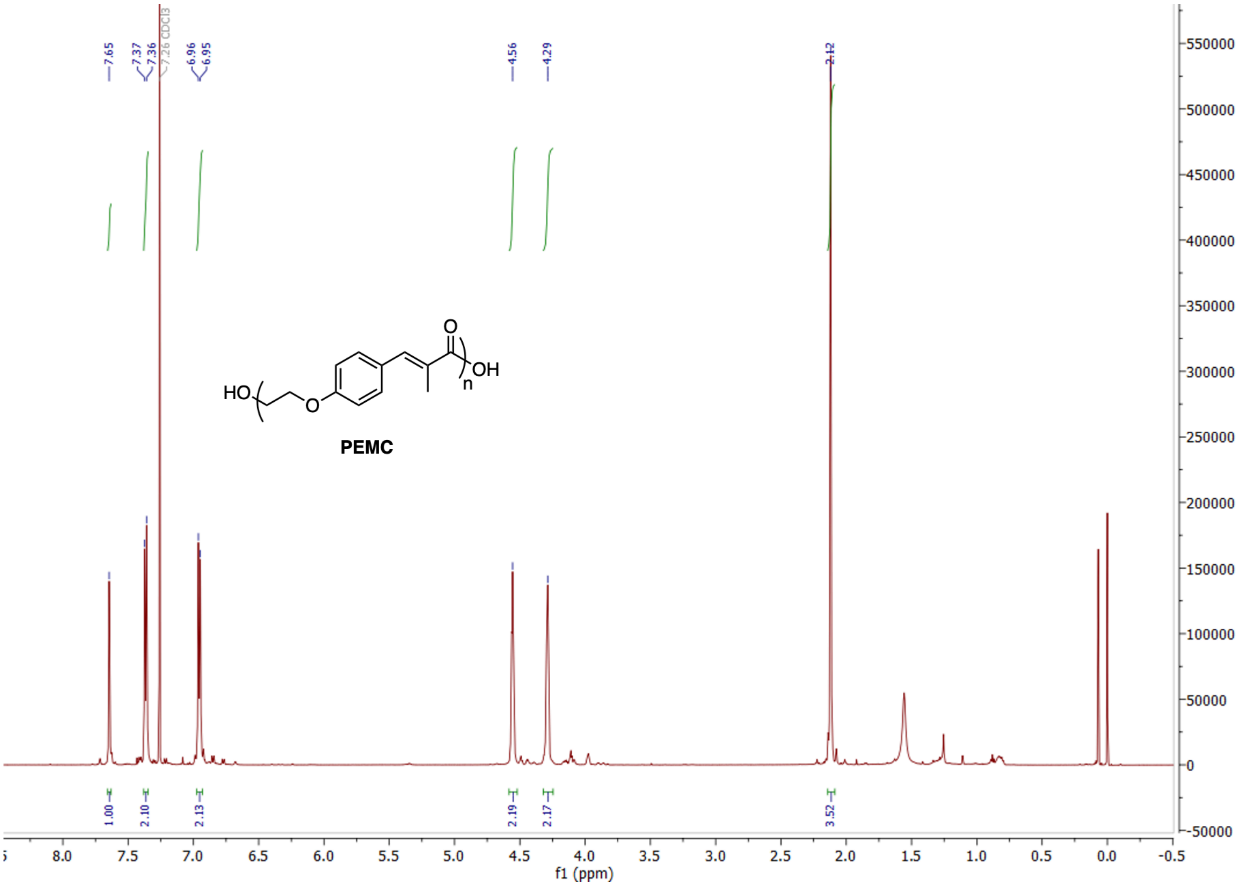

Figure S27. ^{1}H NMR spectrum of poly-ethylene methyl cinnamate (PEMC) in $CDCl_3$.

^{13}C NMR Spectra of Monomers and Polymers

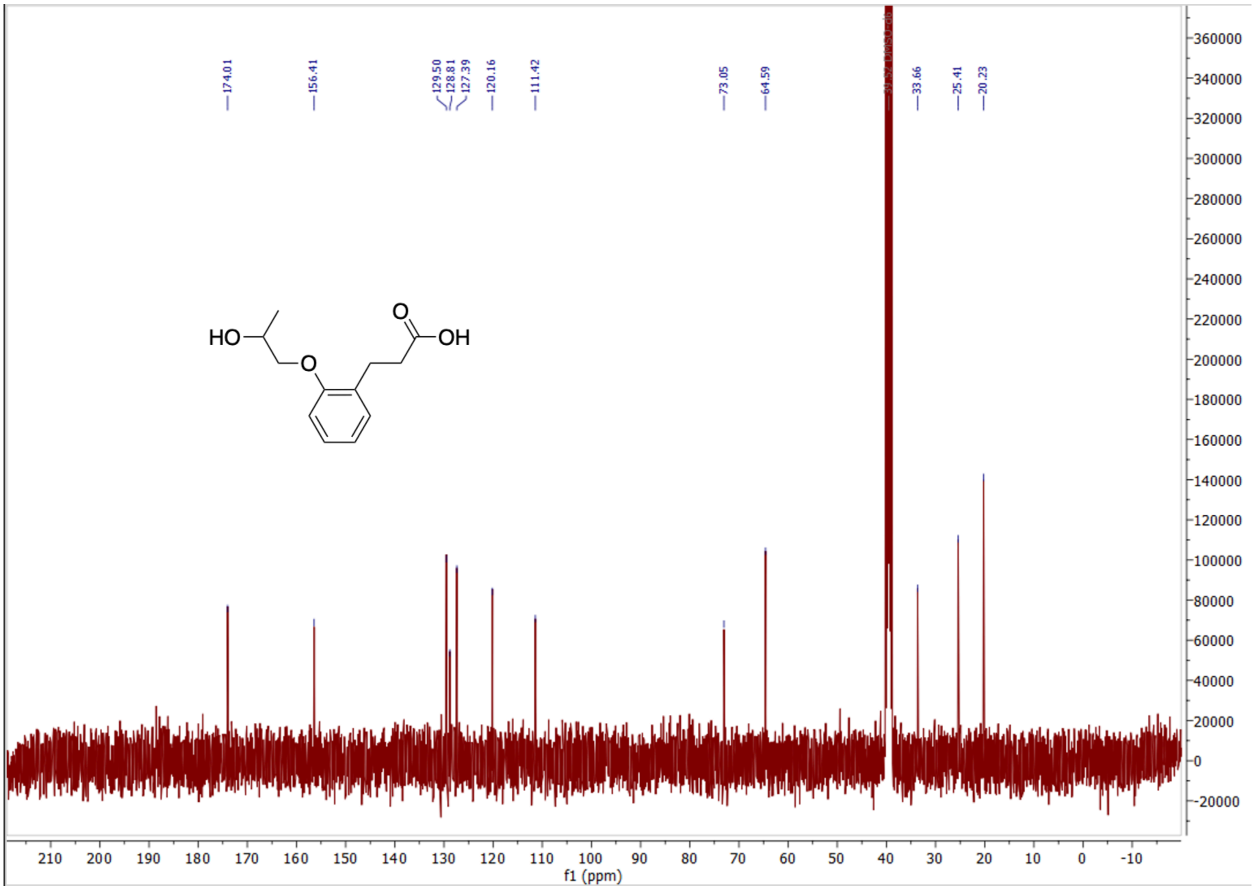

Figure S28. ^{13}C NMR spectrum of 3-(2-(2-hydroxypropoxy)phenyl)propanoic acid in DMSO-*d6*.

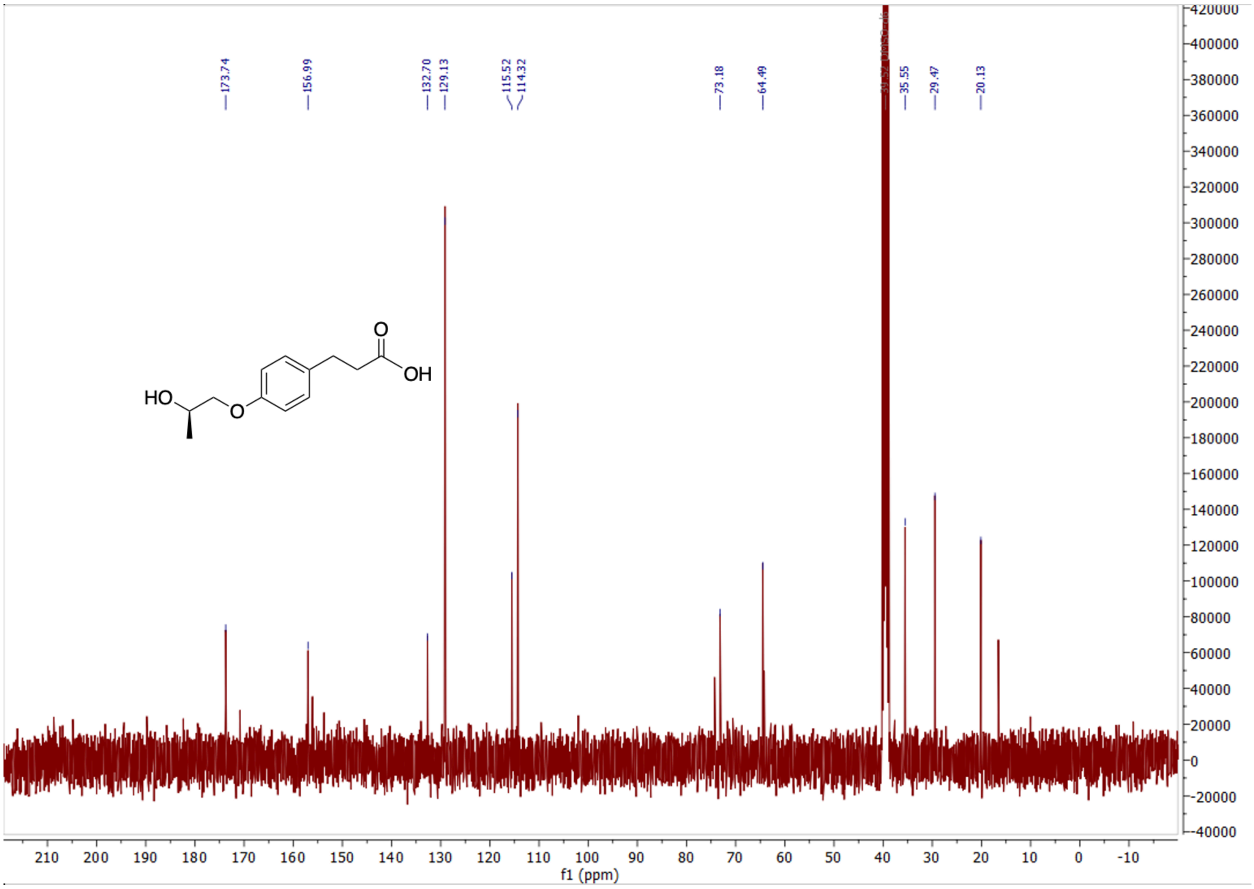

Figure S29. ^{13}C NMR spectrum of (*R*)-3-(4-(2-hydroxypropoxy)phenyl)propanoic acid in DMSO-*d6*.

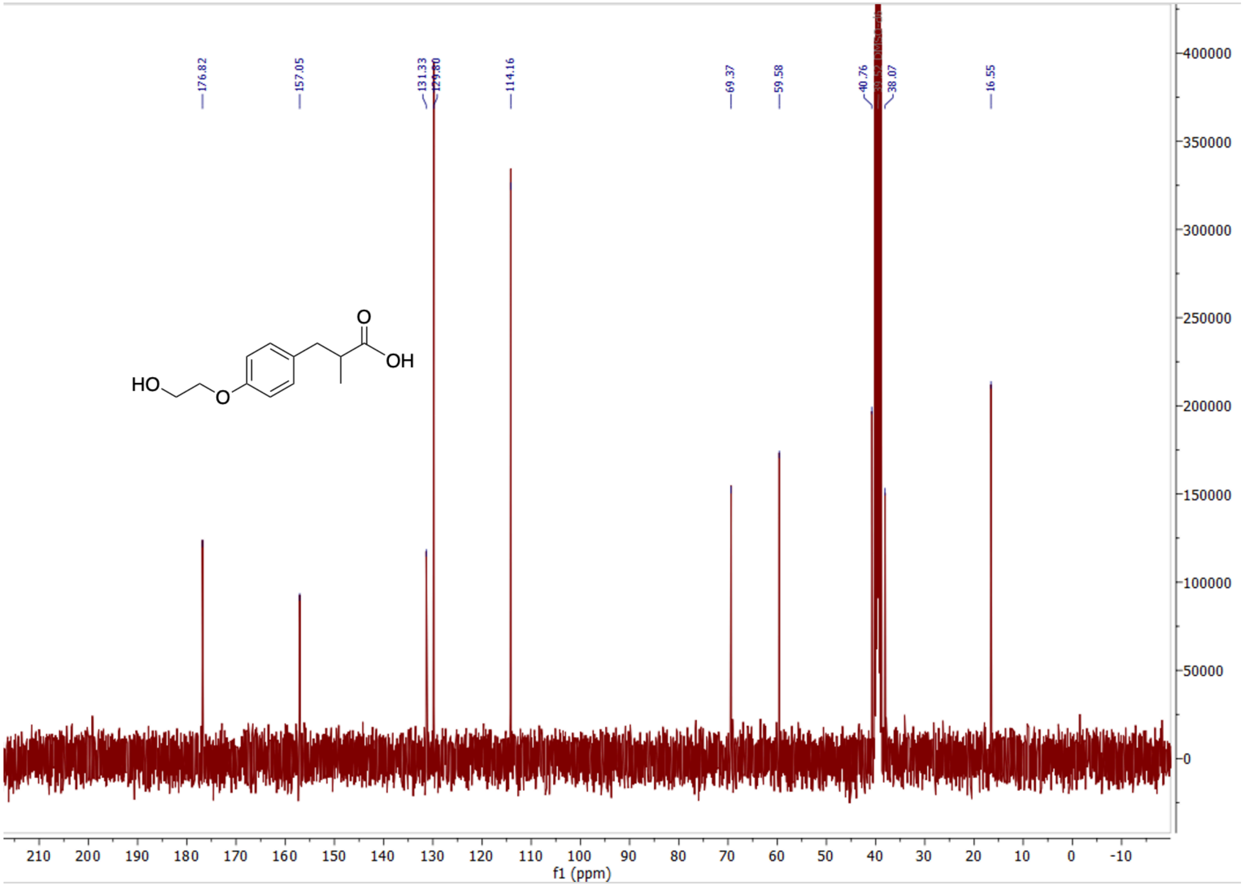

Figure S30. ^{13}C NMR spectrum of 3-(4-(2-hydroxyethoxy)phenyl)-2-methylpropanoic acid in DMSO-*d6*.

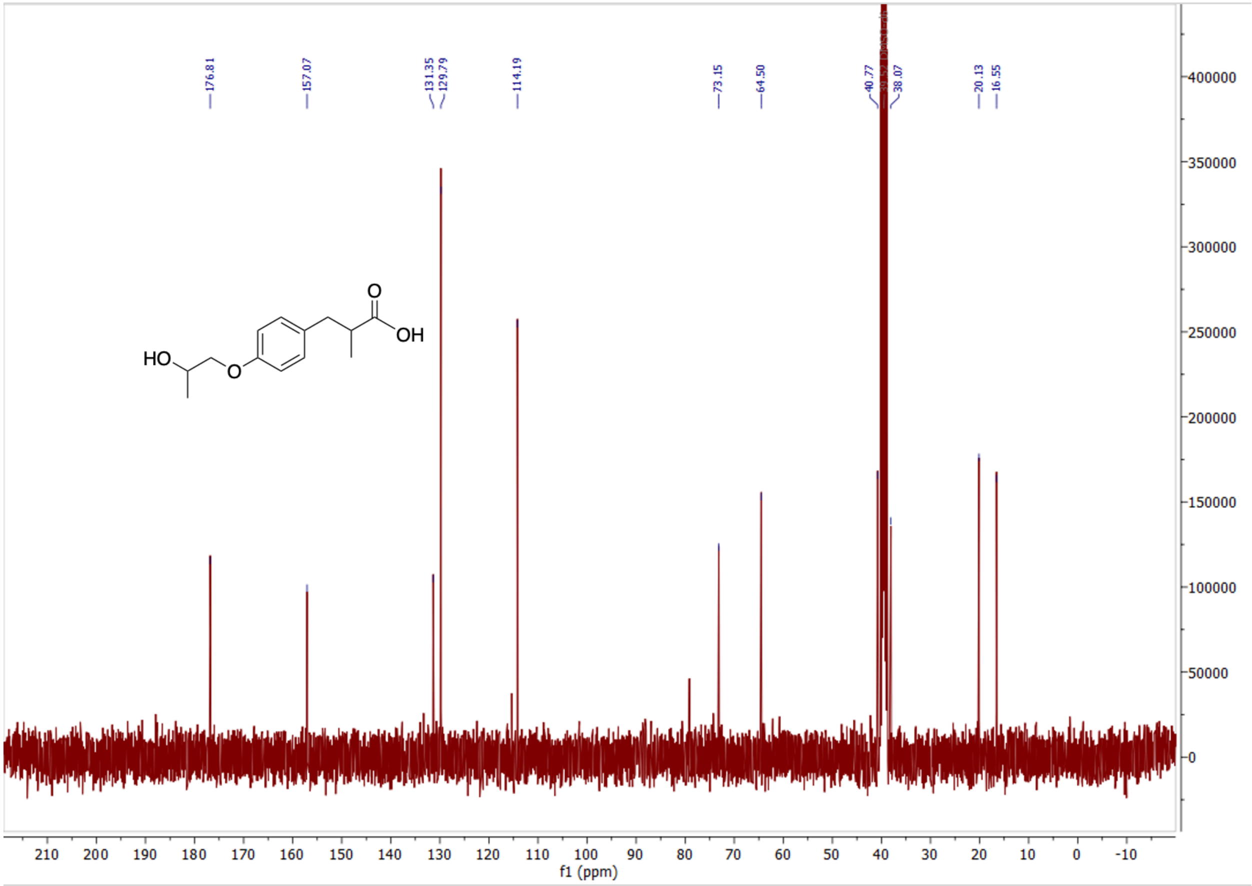

Figure S31. ^{13}C NMR spectrum of 3-(4-(2-hydroxypropoxy)phenyl)-2-methylpropanoic acid in DMSO-*d6*.

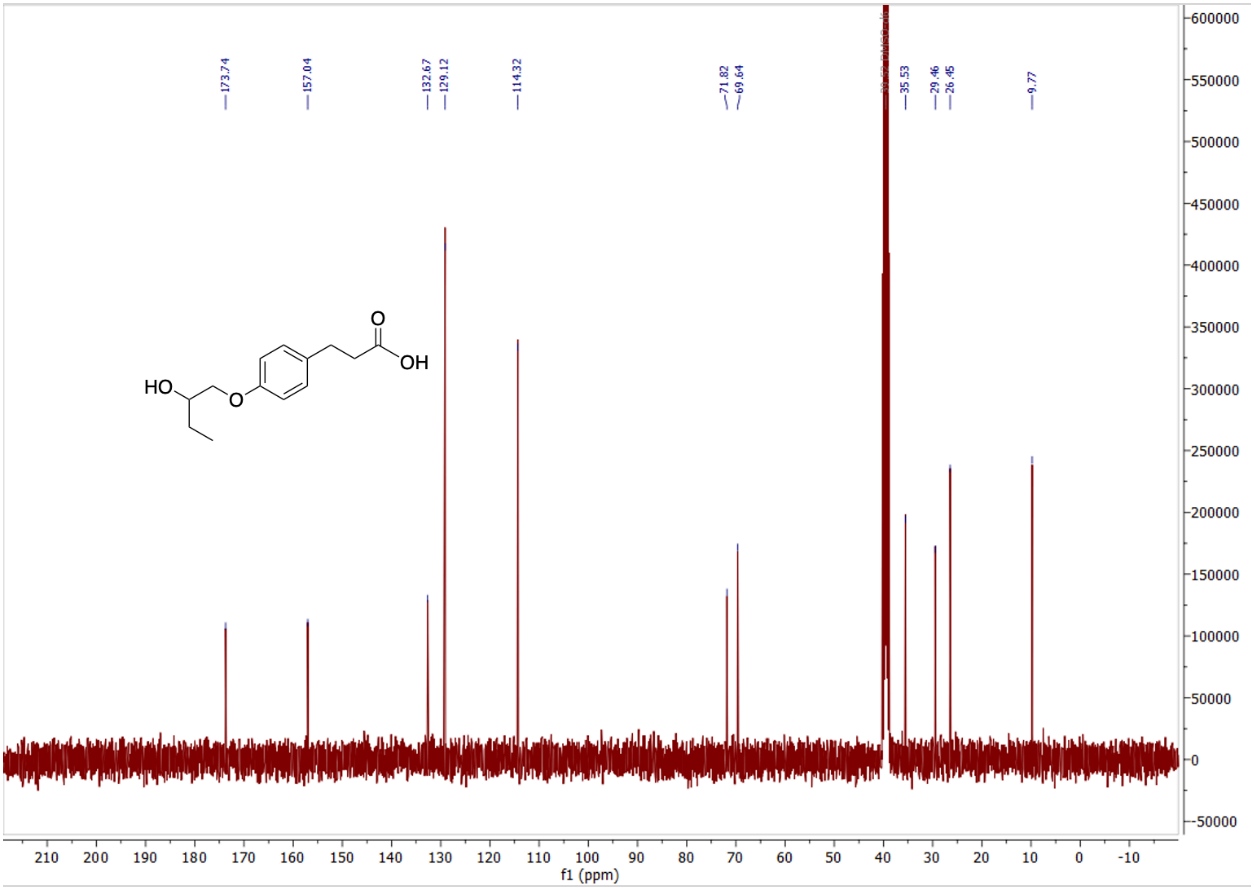

Figure S32. ^{13}C NMR spectrum of 3-(4-(2-hydroxybutoxy)phenyl)propanoic acid in DMSO-*d6*.

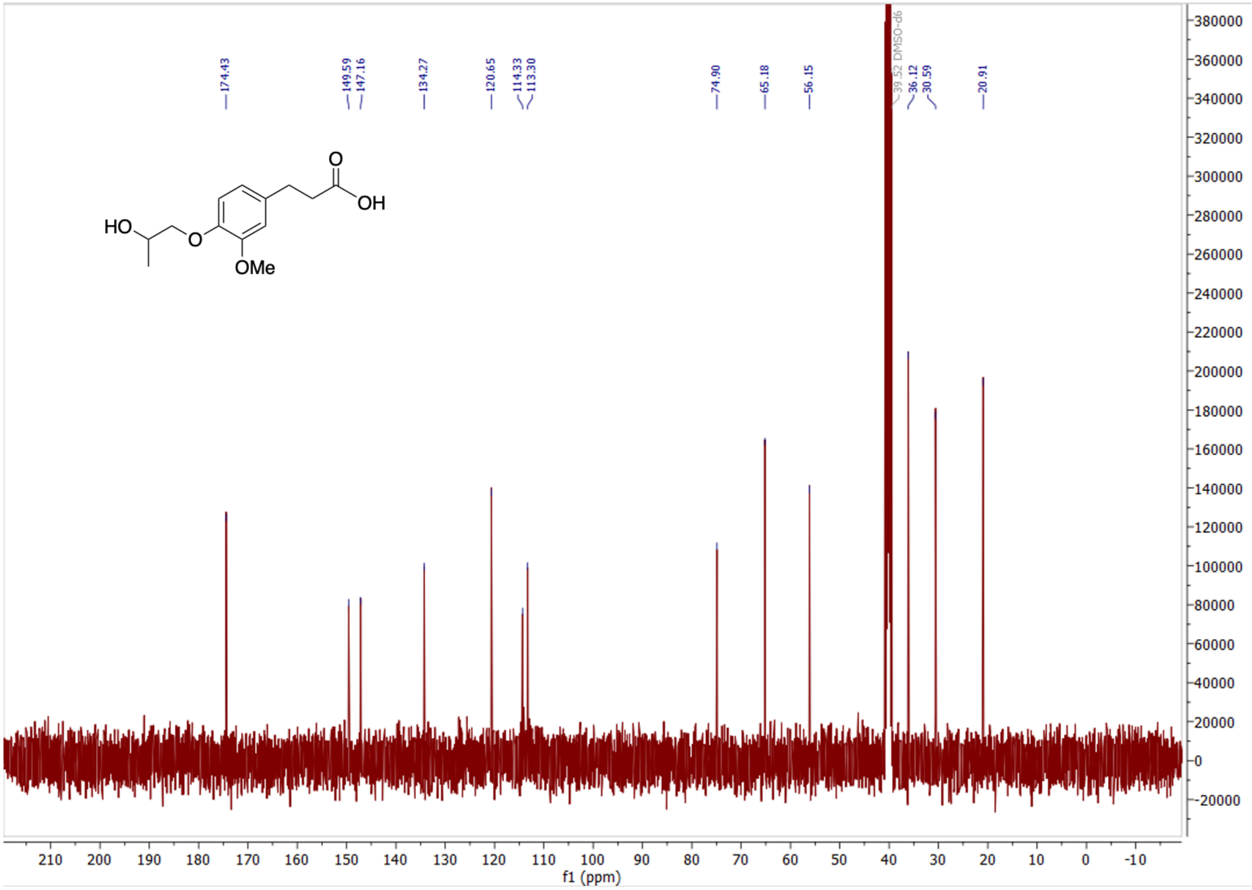

Figure S33. ^{13}C NMR spectrum of 3-(4-(2-hydroxypropoxy)-3-methoxyphenyl)propanoic acid in DMSO-*d6*.

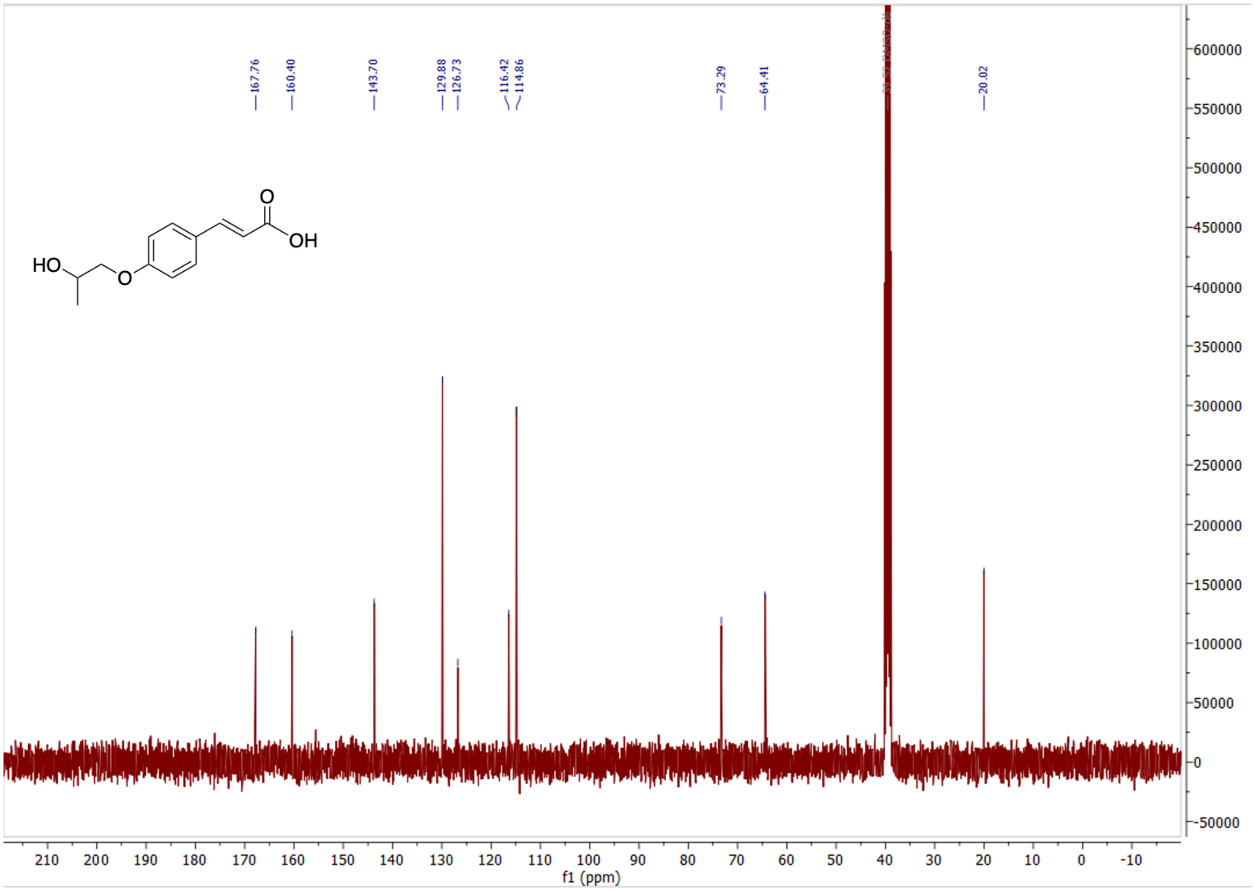

Figure S34. ^{13}C NMR spectrum of 3-(4-(2-hydroxypropoxy)phenyl)acrylic acid in DMSO-*d6*.

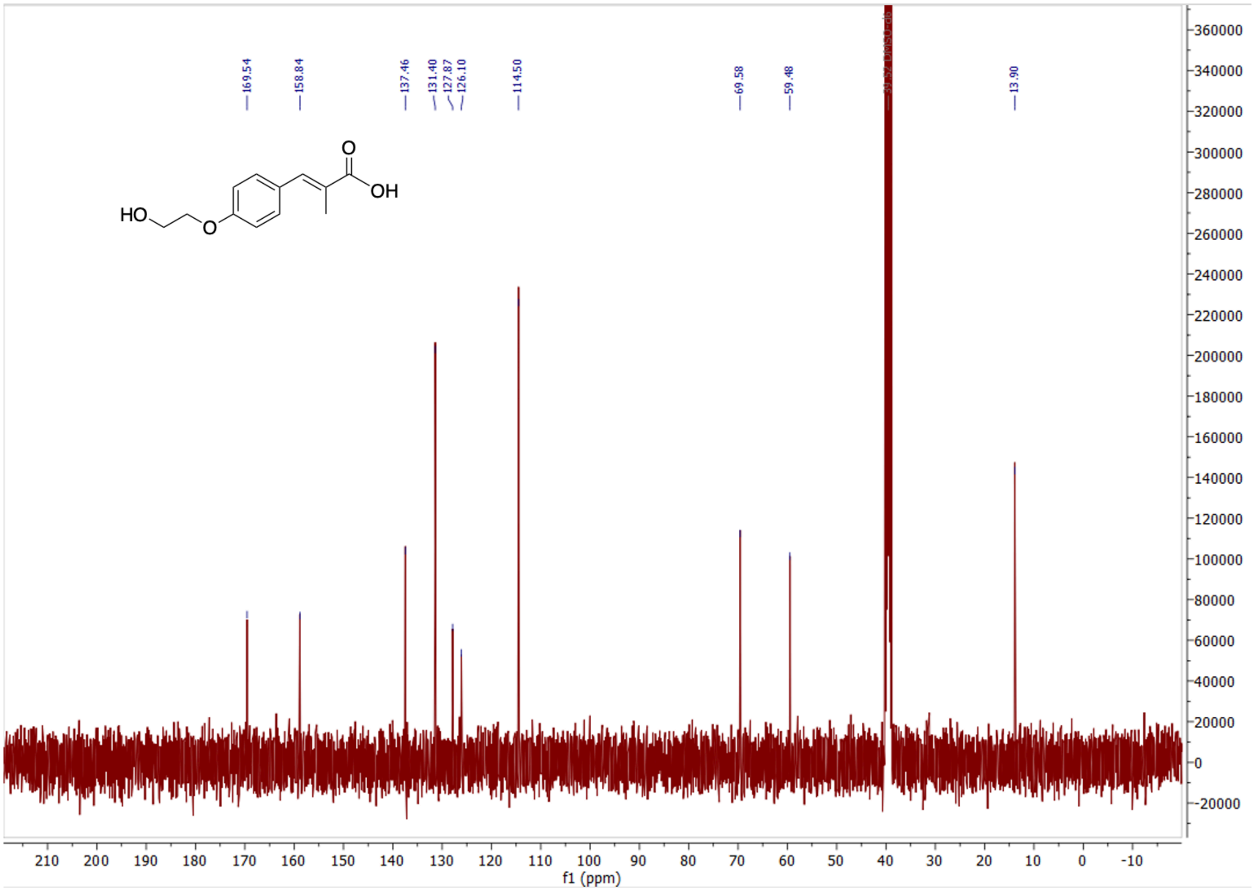

Figure S35. ^{13}C NMR spectrum of (E)-3-(4-(2-hydroxyethoxy)phenyl)-2-methylacrylic acid in DMSO-*d6*.

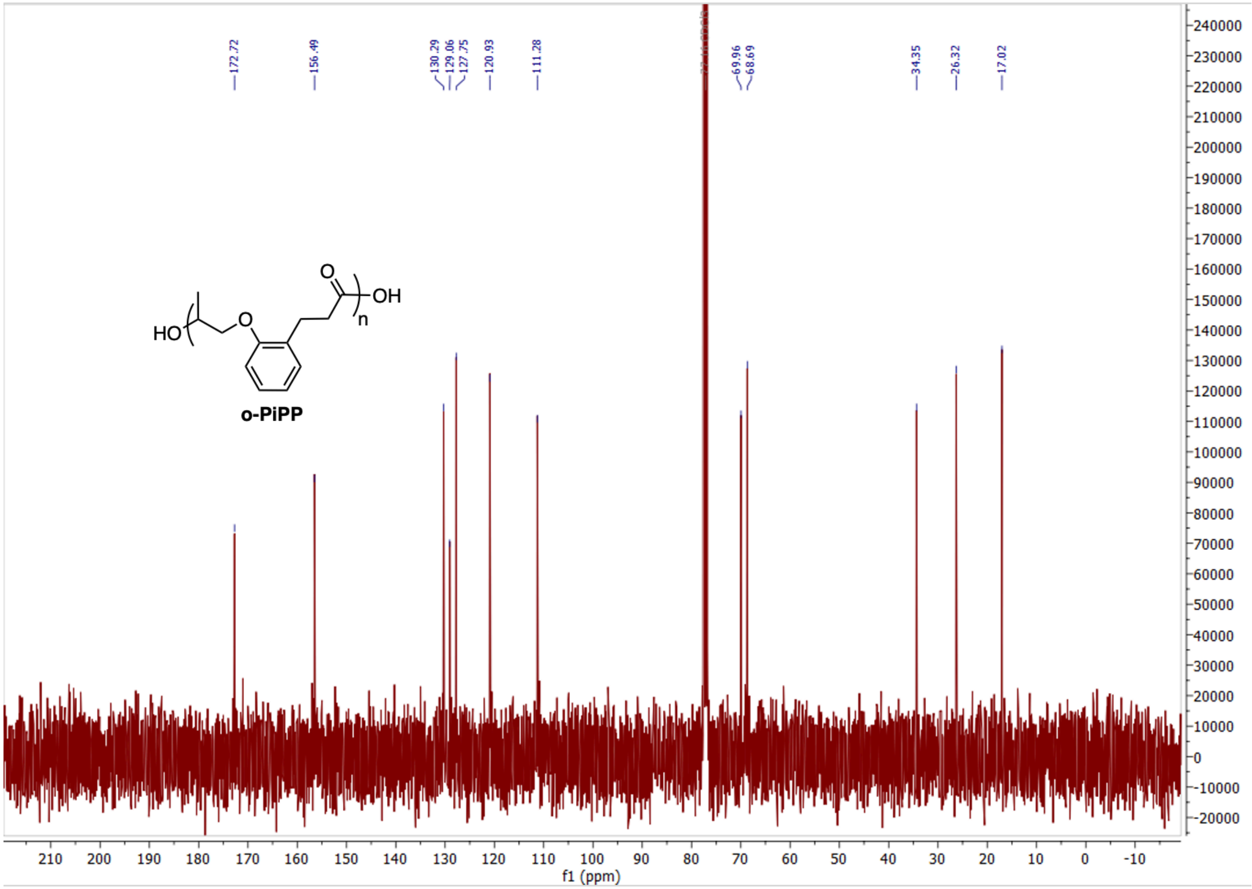

Figure S36. ^{13}C NMR spectrum of poly-ortho-isopropyl phloretate (o-PiPP) in $CDCl_3$.

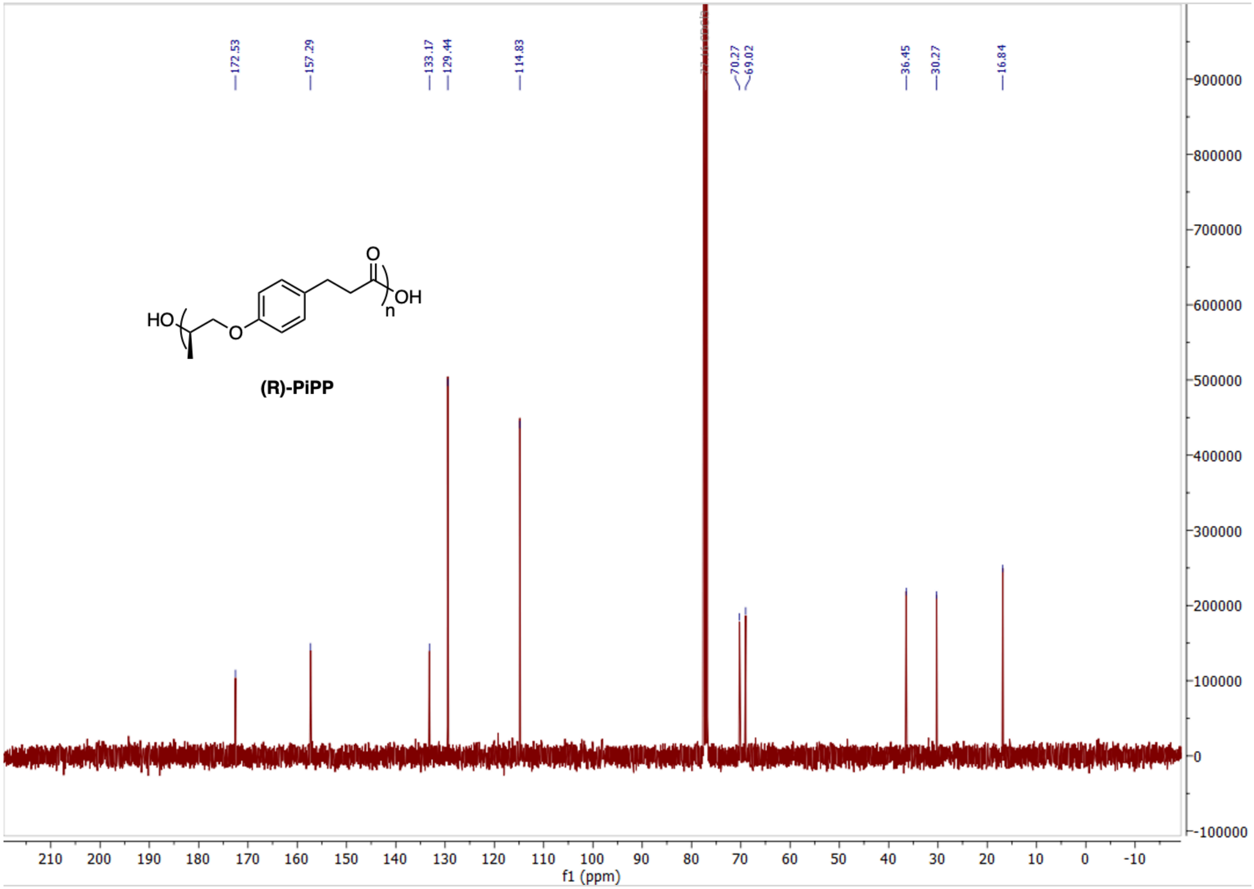

Figure S37. ^{13}C NMR spectrum of (R)-poly-isopropyl phloretate ((*R*)-PiPP) in $CDCl_3$.

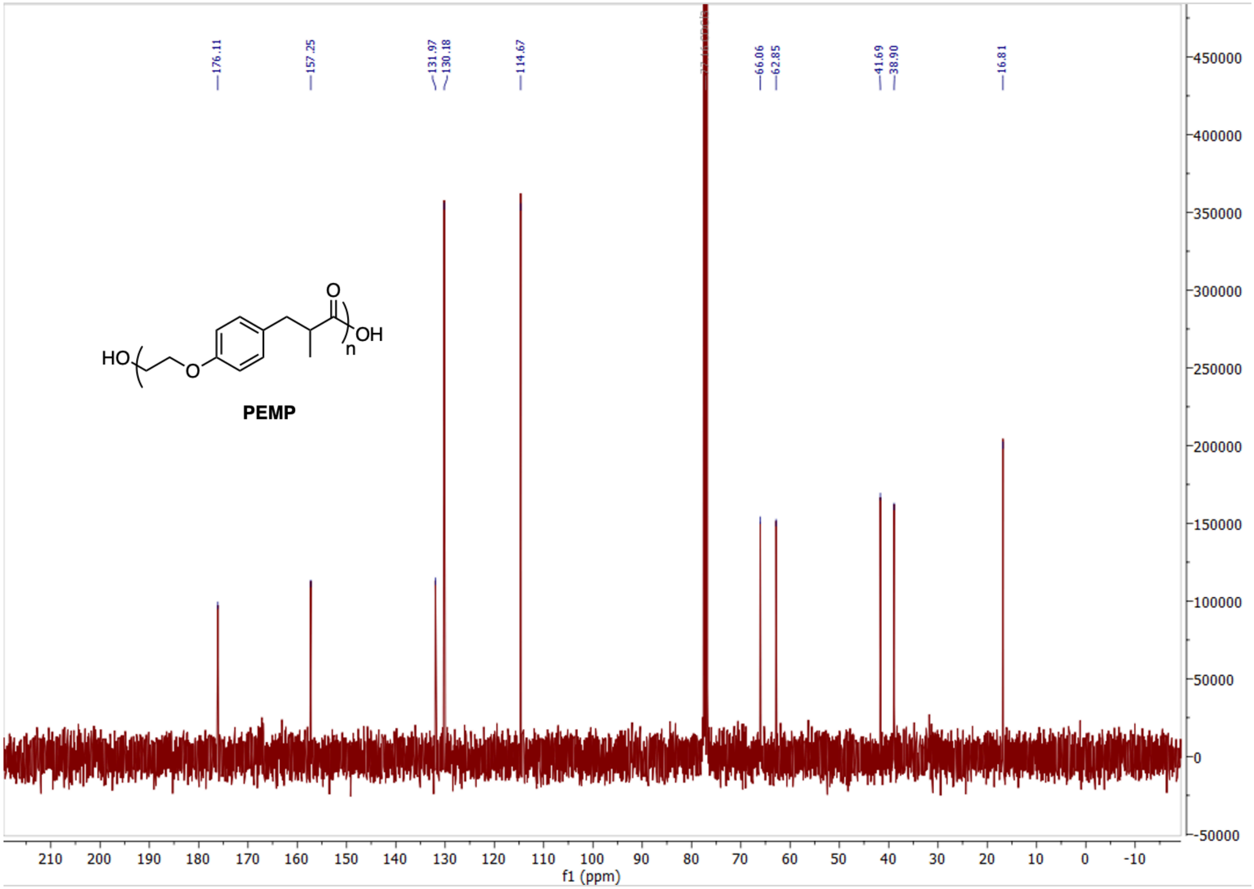

Figure S38. ^{13}C NMR spectrum of poly-ethylene methyl phloretate (PEMP) in $CDCl_3$.

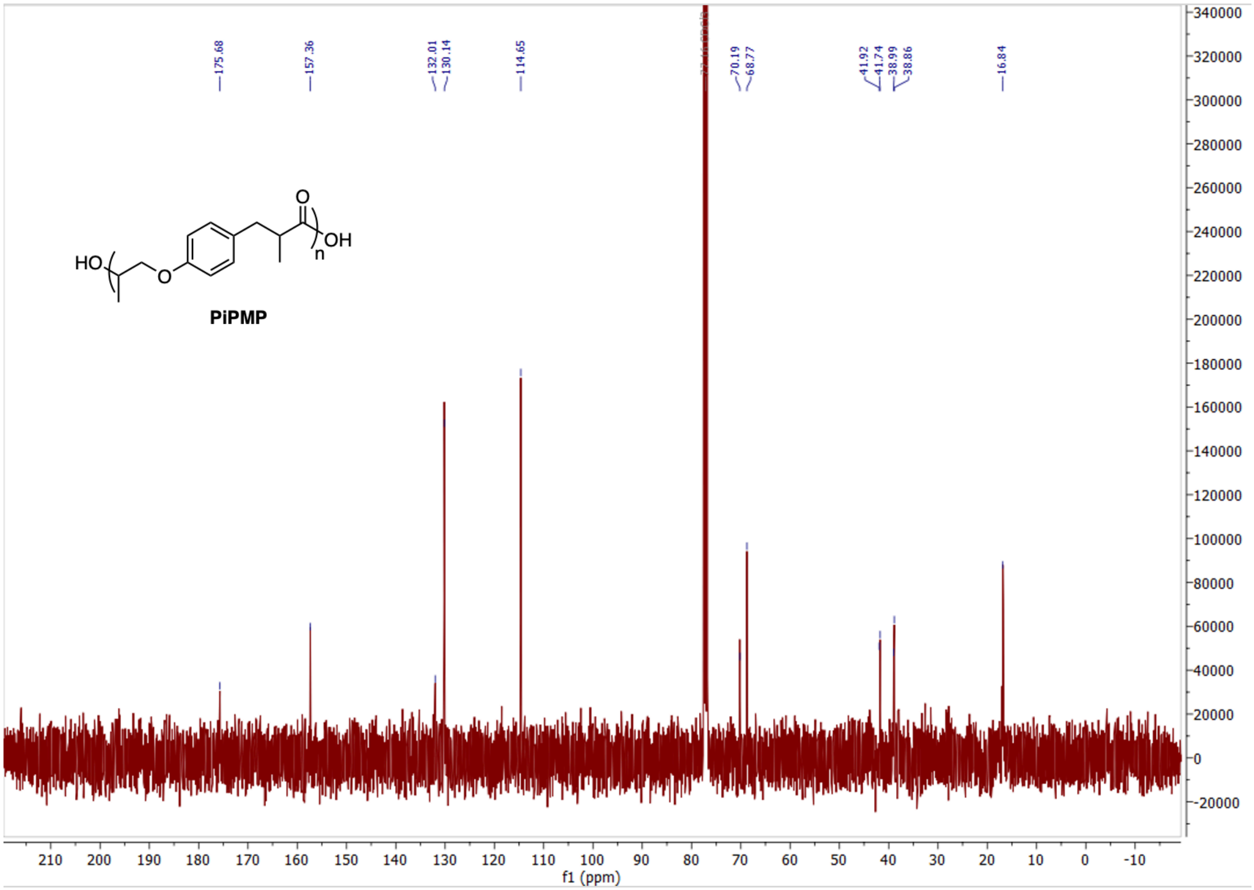

Figure S39. ^{13}C NMR spectrum of poly-isopropyl-methyl-phloretate (PiPMP) in $CDCl_3$.

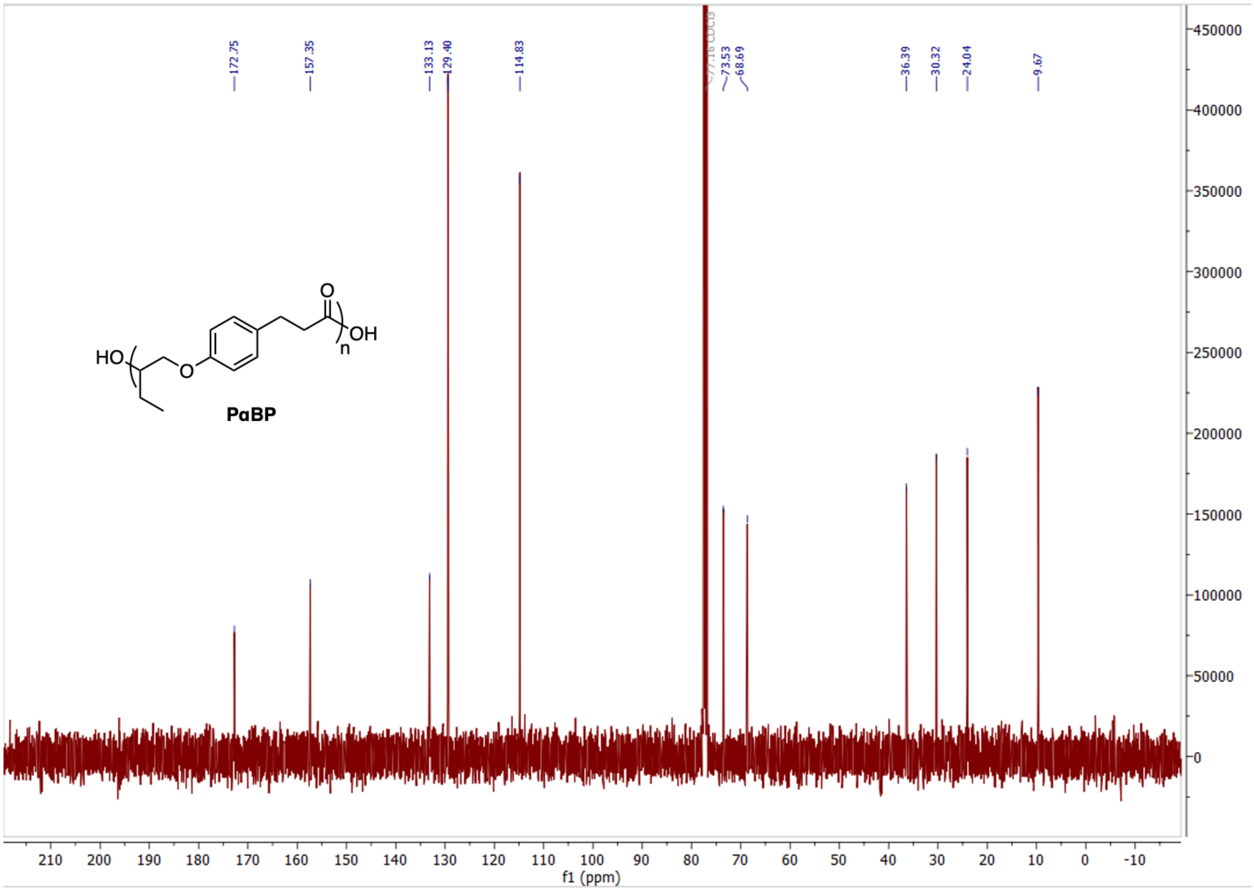

Figure S40. ^{13}C NMR spectrum of poly-α-butylene phloretate (PαBP) in $CDCl_3$.

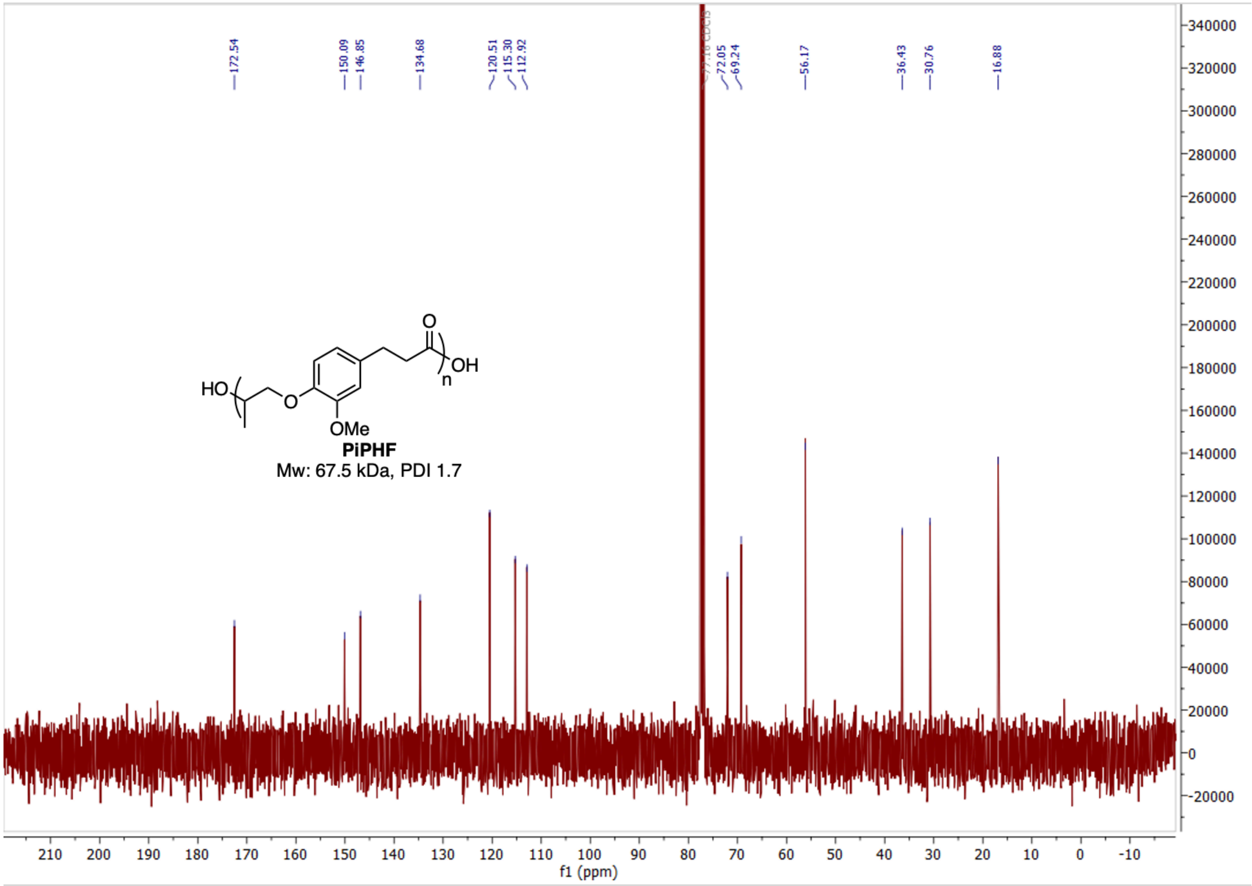

Figure S41. ^{13}C NMR spectrum of poly-isopropyl dihydroferulate (PiPHF) in $CDCl_3$.

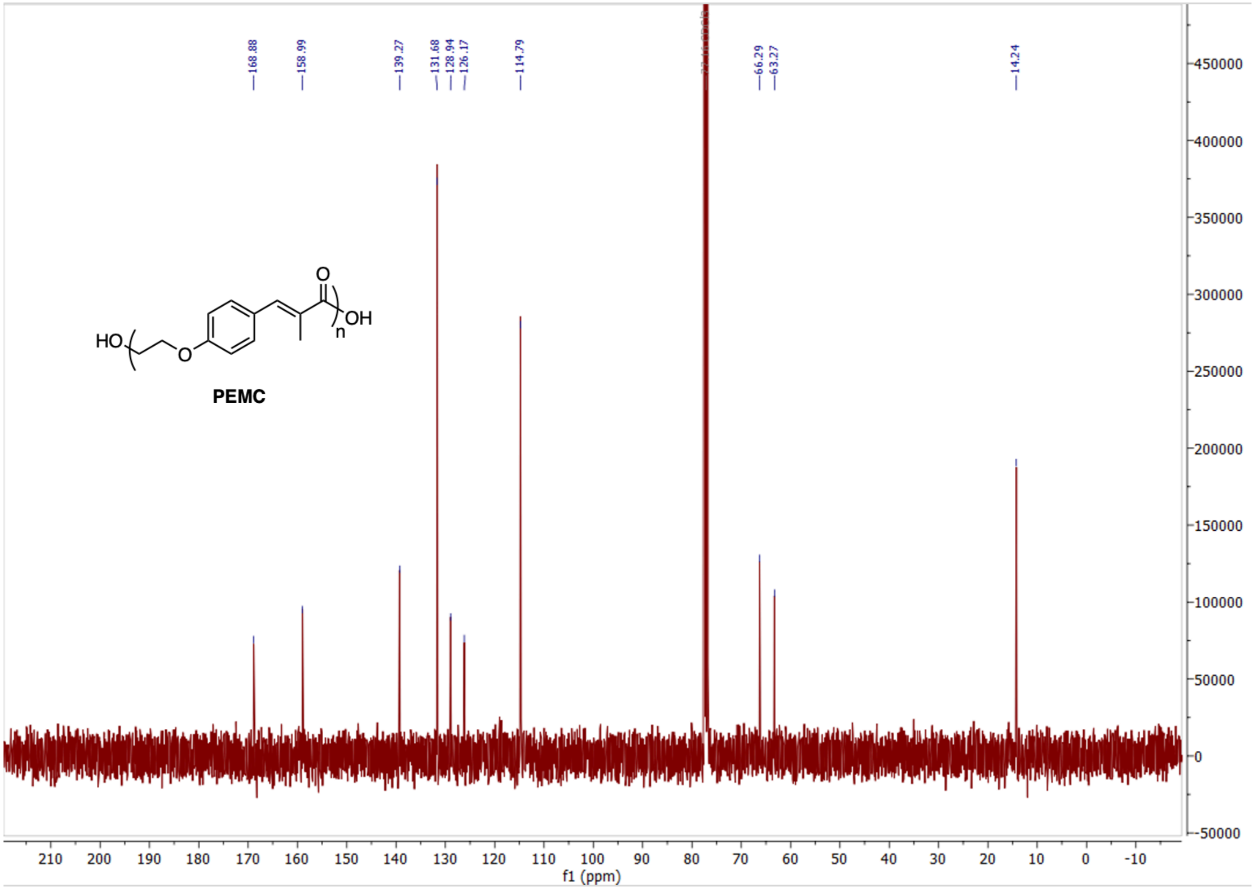

Figure S42. ^{13}C NMR spectrum of poly-ethylene methyl cinnamate (PEMC) in $CDCl_3$.

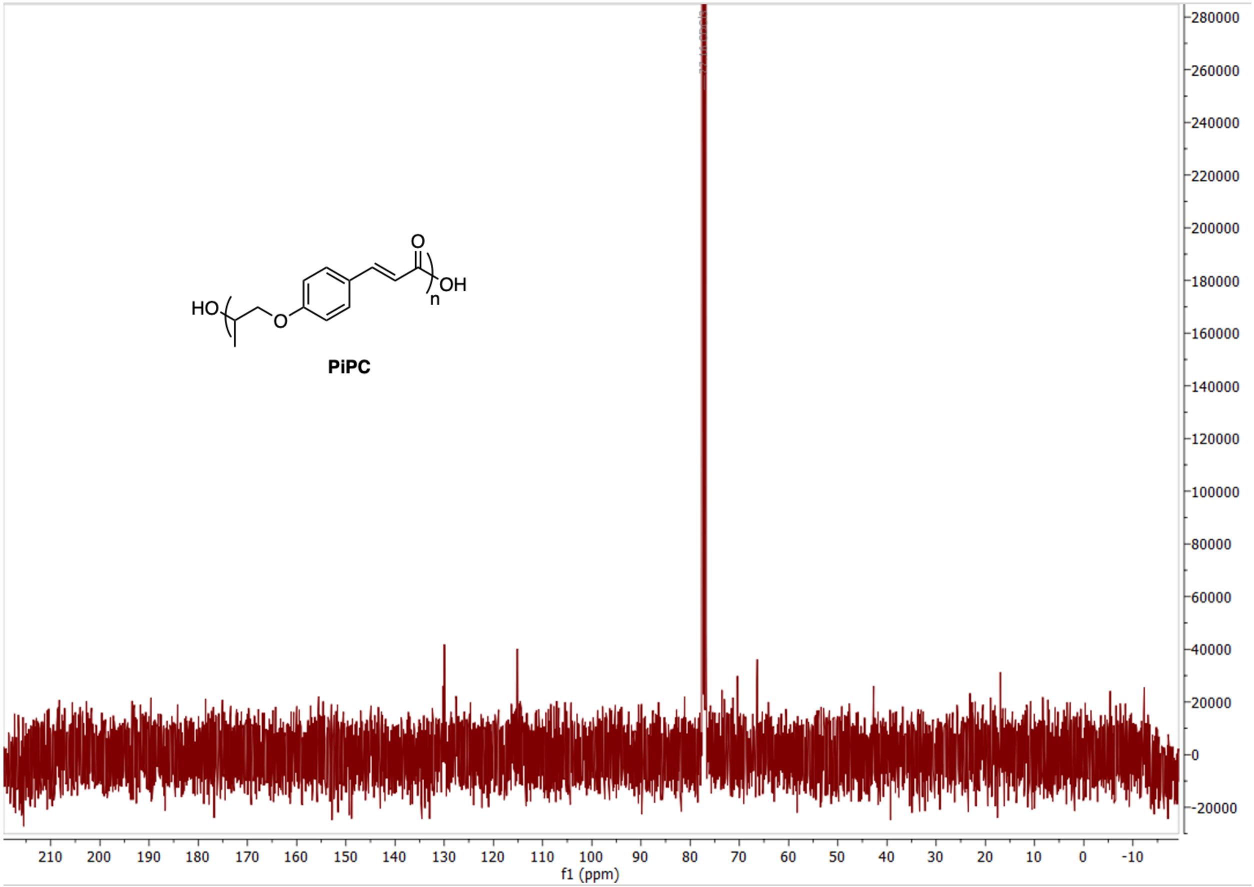

Figure S43. ^{13}C NMR spectrum of poly-isopropyl cinnamate (PiPC) in $CDCl_3$.

GPC Data

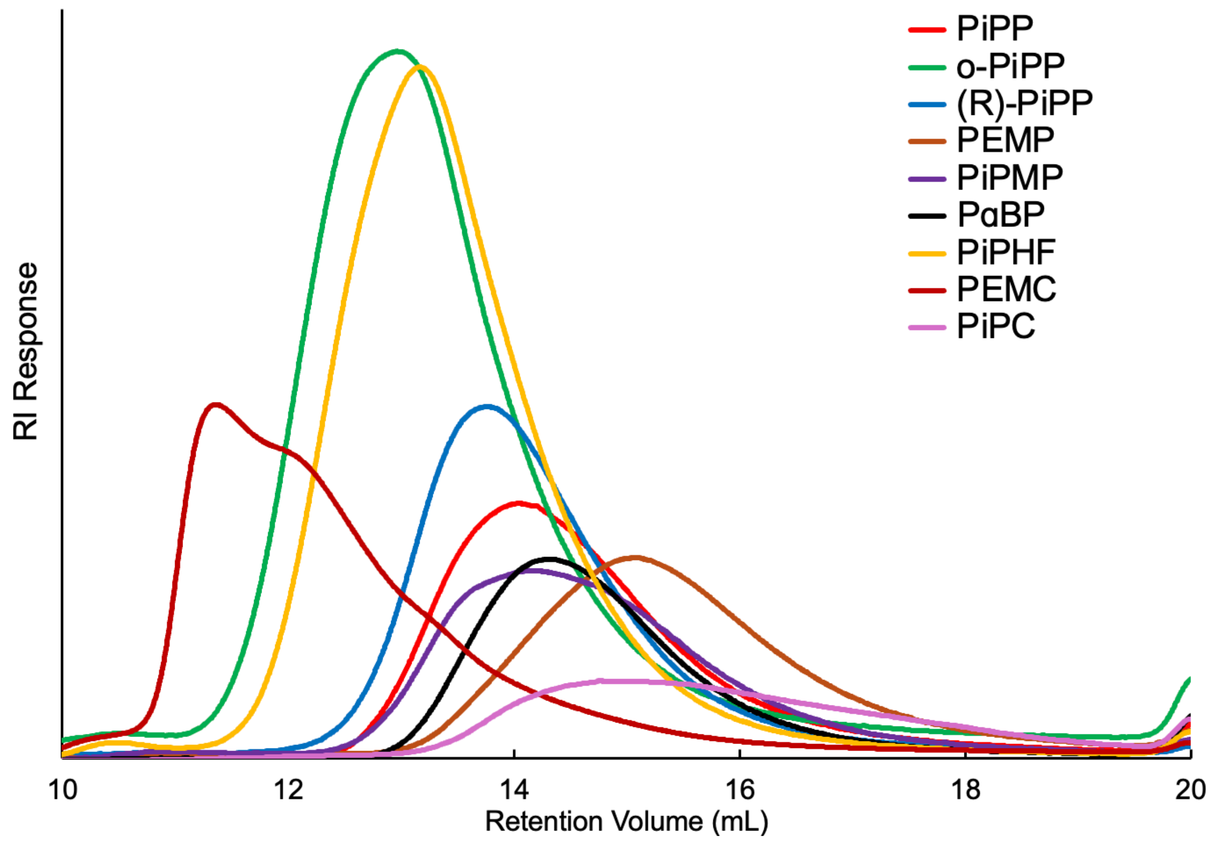

Figure S44. GPC Traces for polyesters synthesized in this study.

Table S2. Summary of molecular weight data obtained in this study in comparison to polystyrene standards.

**Entry**	**Polymer:**	**Mw (kDa):**	***Đ*:**
**1**	PiPP	27.2	1.3
**2**	*o*-PiPP	90.6	1.6
**3**	(*R*)-PiPP	29.0	1.4
**4**	PEMP	22.5	1.4
**5**	PiPMP	22.0	1.7
**6**	PαBP	19.5	1.3
**7**	PiPHF	67.5	1.7
**8**	PEMC	103.0	2.8
**9**	PiPC	41.6	3.7

References:

(1) Marumoto, S.; Miyazawa, M. Microbial reduction of coumarin, psoralen, and xanthyletin by Glomerella cingulata. *Tetrahedron* **2011**, *67* (2), 495-500. DOI: https://doi.org/10.1016/j.tet.2010.10.089.

(2) Fujii, S. K., H.; Wantanabe, S. Phenylcarboxylic Acid Derivatives. USA 1989.

(3) Winfield, D.; Ring, J.; Horn, J.; White, E. M.; Locklin, J. Semi-aromatic biobased polyesters derived from lignin and cyclic carbonates. *Green Chem* **2021**, *23* (23), 9658-9668. DOI: 10.1039/d1gc03135j.
